# An integrated mutation-based immunoinformatic approach incorporating variability in epitopes: a study based on HIV subtype C

**DOI:** 10.3389/fimmu.2025.1540253

**Published:** 2025-05-20

**Authors:** Saurav Kumar Mishra, Neeraj Kumar, Md. Harun Or Rashid, Sharifa Sultana, Turki M. Dawoud, Mohammed Bourhia, John J. Georrge

**Affiliations:** ^1^ Department of Bioinformatics, University of North Bengal, Darjeeling, West Bengal, India; ^2^ Department of Pharmaceutical Chemistry, Bhupal Nobles’ College of Pharmacy, Udaipur, Rajasthan, India; ^3^ School of Engineering, Macquarie University, Sydney, NSW, Australia; ^4^ Computational Biology Research Laboratory, Department of Pharmacy, Faculty of Health and Life Sciences, Daffodil International University, Dhaka, Bangladesh; ^5^ Department of Botany and Microbiology, College of Science, King Saud University, Riyadh, Saudi Arabia; ^6^ Department of Chemistry and Biochemistry, Faculty of Medicine and Pharmacy, Ibn Zohr University, Laayoune, Morocco

**Keywords:** HIV, epitope, mutation, vaccine, TLR3, simulation

## Abstract

Currently, HIV (human immunodeficiency virus) infection is one of the leading complications in public health and causes acquired immunodeficiency syndrome (AIDS), especially in the African region. No specific vaccine is available to combat this, with multi-strain variability being one of the hurdles. In this investigation, we employed variability in the epitope of the HIV subtype C targets to introduce mutations and construct an epitope-based vaccine. Four targets were examined to predict the B and T cells (major histocompatibility complex class I and II). Among the predicted epitopes, immunodominant epitopes were selected and were mapped with the identified variable amino acid to incorporate mutation. These selected and mutated epitopes were used for the non-mutated and mutated vaccine construction, considering linker for fusion and adjuvant to improve the activity. The vaccine’s structure was modeled and examined to validate its structural quality, and a high population coverage was also found. The docking investigation of the non-mutated and mutated vaccine with Toll-like receptor 3 shows remarkable activity followed by strong binding affinity, and the simulation of over 100 ns revealed the constancy of the complex system. The immune response revealed its strong effectiveness by generating multiple immunoglobulins followed by the time step of infection, and further, *in silico* cloning demonstrated a high expression in *Escherichia coli* based on their favorable Codon Adaptation Index and GC value. The integrated approach in this investigation will help to plan a potent immunodominant vaccine that can work for multiple strains of HIV infection.

## Introduction

Acquired immunodeficiency syndrome (AIDS) is an ongoing public health concern caused by HIV (1, 2). According to a recent World Health Organization (WHO) report, nearly 39.9 million are living with HIV; at the end of 2023, approximately 1.3 million people acquired the infection, while 63,000 died due to HIV-related complications (https://www.who.int/news-room/fact-sheets/detail/hiv-aids) (3). Moreover, the WHO African region remains to have the highest HIV burden (https://www.who.int/data/gho/data/themes/hiv-aids). The AIDS pandemic is led by the two types of HIV, i.e., HIV-1 and HIV-2, with the former being more prevalent than the latter (4, 5). Moreover, several antiretroviral therapies (ARTs) were designed and used, but none of them will lead to combatting this infection completely (6, 7). A few vaccines were developed to combat this, but they did not prove efficient due to a lack of appropriate immune response and effectiveness. Apart from that, one of the hurdles behind the efficiency is the variability and the mutation within the strains. Despite the various hurdles, the most effective vaccine, RV144, was developed, providing only 31.2% protection against this infection (7–9). This emphasizes the need to create a potent vaccine to address the challenges of strain variability due to mutations in controlling HIV infection. HIV-1 is classified into four (M, N, O, and P) groups; among them, only group M causes 95% to be classified into various subtypes (A, B, C, D, F, G, H, J, and K) (10–12). However, subtype B is prevalent in Australia, America, and Western Europe, whereas subtype C is prevalent in Africa and India (10, 11). Moreover, subtype C is the most prevalent strain worldwide (46.6%) and dominates in Asia and Africa, followed by subtypes A and B (13–15). Furthermore, a recent systematic review reported that subtype C accounted for 50.4% of worldwide HIV based on data (from 2016 to 2021) and found a significant increase in the cases compared to the previous dataset (from 2010 to 2015) (16). HIV employs various strategies to evade immune surveillance, including antigenic variation, MHC downregulation, and immune cell dysfunction (17). Subsequently, several key mutations are mainly responsible for escaping immune mechanisms, such as N332 glycan shift (escape broadly neutralizing antibodies by altering glycan shielding) and T242N (reduces recognition by CTL), among others (18, 19).

The HIV genome comprises several effective structural, regulator, and accessory genes. However, structural genes, i.e., the envelope glycoprotein, protease, reverse transcriptase, and integrase, are crucial for host–pathogen interaction and its replication (5, 11, 20, 21). Their role in viral mechanisms makes them an ideal candidate for therapeutic development. At present, using immunological data, immunoinformatics-assisted vaccine design has been identified as a suitable strategy, along with reverse vaccinology and advanced computational approaches (11, 22–25) targeting several other pathogens, because time efficiency, cost-effectiveness, and high accuracy are essential for a successful vaccine design. Immunoinformatics-assisted studies on HIV have successfully targeted various components, including gp120 (21), the whole HIV genome (5), and Gag polyprotein (7), among others (11, 20), highlighting the reliability of this approach without incorporating the variability of epitopes. In addition, no such study was reported on subtype C that contributed to higher HIV infection than the other strains in group M. The main hurdle in combating HIV is the strain variability caused by mutations, which has yet to be fully addressed (26–28) and also remains a major obstacle behind the successful vaccine formulation and the effective potent activity of the available vaccine. Moreover, a few studies were designed to incorporate mutation within epitopes against COVID-19 successfully (22, 29). Compared to conventional vaccine design methods, the advantage of employing immunoinformatics enables the screening of potential epitopes that are effective for multiple strains, the assessment of their immune activity, and other key factors to enhance vaccine development, which is vital for the effective vaccine design (5, 7).

Therefore, this study examined subtype C to formulate a potent vaccine considering variability. The B cells and MHC class I and class II (MHC I and MHC II) epitopes were identified and selected based on their high antigenicity score in this investigation. These epitopes were further mapped considering the variability of amino acids identified via multiple strains. The non-mutated and mutated vaccines were formulated, and their molecular activity and stability toward the TRL were analyzed via docking and dynamics. The immune activity based on the vaccination steps and the expression of the formulated vaccine were performed and analyzed.

## Materials and methods

**Figure 1:**
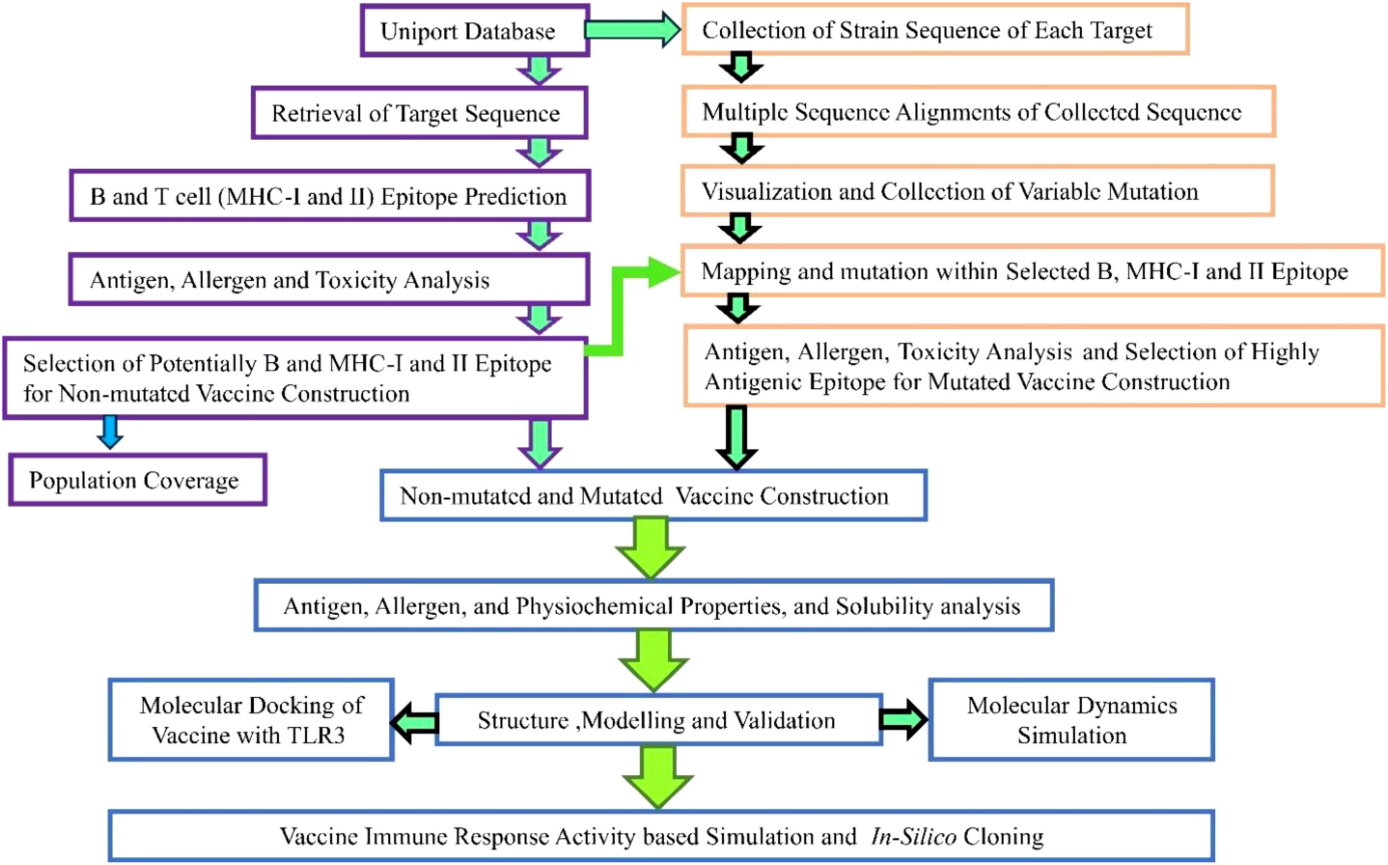
Overview of employed steps in the designed study.

The employed steps corresponding to the methodology are illustrated in **Figure 1**.

### Collection of the target sequence and their immune assessment

The vital target (essential for host–pathogen interaction, replication, and pathogenesis) sequences within the HIV mechanism were retrieved from UniProt (https://www.uniprot.org/). The vaccine protein must have strong immunological properties and be non-allergenic to confirm a potent immune response (29). These retrieved sequences were further subjected to the antigen and allergen assessment via VaxiJen v2.0 (https://www.ddg-pharmfac.net/vaxijen/VaxiJen/VaxiJen.html) (30) considering virus as a target and a threshold value of 0.4 and the AllerTOP v.2.0 (https://www.ddg-pharmfac.net/allertop_test/) (31) server. The VaxiJen server is mainly based on alignment-based prediction methods, while the AllerTOP server is alignment-free and grounded on the target’s physicochemical properties.

### Identification of B-cell epitope

Two subsequent servers—ABCpred (http://crdd.osdd.net/raghava/abcpred/) (32), which utilized the artificial neural network, and BepiPred 2.0 (http://tools.iedb.org/bcell/) grounded on the sequence features of the antigen (33), available at IEDB—have different algorithms to detect more potential linear B-lymphocyte (LBL) epitopes considering collected sequences as input with default parameters. For peptide vaccines, recognizing B cells is crucial, as their receptors recognize peptides to trigger an effective immune response (29). However, the epitopes were further considered based on their presence in both servers and examined via VaxiJen v2.0 (30), AllerTOP v.2.0 (31), and ToxinPred (https://webs.iiitd.edu.in/raghava/toxinpred/) along with default parameter (34) servers.

### Identification of T-cell (MHC I and MHC II) epitopes and their immune assessment

CD8^+^ T lymphocytes recognize MHC I epitopes. When a cell is infected or has aberrant proteins (such as in viral infections or cancer), MHC I molecules present these peptides on the cell surface, prompting CD8^+^ T cells to kill the infected or abnormal cells (35–37). On the other hand, CD4^+^ helper T cells recognize MHC II epitopes. Antigen-presenting cells (APCs) internalize and process foreign antigens, presenting peptides on MHC II molecules to activate CD4^+^ T cells, which then help coordinate the broader immune response (38, 39). The MHC I and MHC II within the targets were identified using Tepitool (http://tools.iedb.org/tepitool/), which computes the epitopes based on seven prediction methods (IEDB recommended, consensus, NetMHCIIpan, NN-align, SMM-align, Sturniolo, and the combinatorial library method) (40). For MHC I, 27 and MHC II, 7, the most frequent alleles with the restricted 9- and 15-mer length were selected, and all other IEDB-recommended parameters were selected (24, 29, 40). Furthermore, the immune assessment was done similarly to the abovementioned one to screen out the potential epitopes.

### Epitope mapping of B and T cells with the variable amino acid

To formulate a mutation-proof vaccine, the designed vaccine should be highly effective in both mutated and non-mutated forms (29, 41). The available sequence concerning each target was collected from UniProt (https://www.uniprot.org/). These sequences were subjected for multiple sequence alignments via Clustal Omega (https://www.ebi.ac.uk/jdispatcher/msa/clustalo), which is based on the seeded guide trees and the HMM technique (42), and the variable amino acid was visualized and collected using the JalView (43) software. These variable amino acids were further mapped with the final selected B- and T-cell epitopes to incorporate mutation.

### Vaccine formulation and immune and physiological assessments

The highly antigenic score followed by non-allergenic and non-toxic-based LBL, MHC I, and MHC II epitopes were selected from each target for the vaccine formulation, leading to a robust immune response against the infection. These epitopes were joined via different subsequent linkers (EAAAK, AAY, KK, and GPGPG) (21, 41). Furthermore, to enhance, activate, and purify, the adjuvant, PADRE, and His-tag were also attached at the N and C terminals of the vaccine construct. In contrast, His-tag was attached using the RVRR linkers (5, 7, 11, 21). Moreover, considering combination, six different vaccines were constructed to identify additional potential combinations with high antigenic properties (score). However, the adjuvant (beta-defensin), PADRE at the N, and His-tag at the C terminal were kept in different distinct vaccine constructs (44).

Moreover, the EAAAK offers an extended, uncharged spacer that can reduce steric hindrance in the region, AAY enhances the immunogenicity and improves pathogen-specific immunity while reducing junctional immunogenicity, KK linkers enhance solubility and are crucial proteases required for antigen processing, GPGPG linkers will aid to avoid aggregation and sustain flexibility, and His-tag is vital for the recognition and separation and facilitates efficient purification (7, 29, 44–46). The antigen and allergen predictions were used similarly to those mentioned above to identify vaccine candidate combinations with optimal immunological and antigenic properties. The combination with the highest antigen score was analyzed for its physicochemical activity via the ProtParam server (https://web.expasy.org/protparam/) (47), considering default parameters. The selected vaccine combination also underwent solubility analysis via Protein-sol (https://protein-sol.manchester.ac.uk/) (48), which is based on weighted scores considering default parameters.

### Population coverage analysis of the selected MHC I and MHC II epitopes

The selection of potential must be validated based on its population coverage, which can be crucial for vaccine development and helpful for most of the world’s population (29). The final MHC I and MHC II epitopes with their restricted alleles were utilized for the analysis via population coverage (http://tools.iedb.org/population/) (49), which estimates the fraction of responders to epitopes with known MHC restrictions.

### Mutated vaccine formulation and immune and physiological assessments

The variable positions identified through multiple sequence alignment were mapped onto the selected epitope to introduce variability and design a mutated epitope to formulate a mutated vaccine that can be helpful in combating multi-strain. The mutated vaccine was constructed, and its immune and physiological assessments were performed similarly to those of the non-mutated vaccine.

### Structure modeling and quality assessment

The SOPMA (based on the homology modeling) (https://npsa-prabi.ibcp.fr/cgi-bin/npsa_automat.pl?page=/NPSA/npsa_sopma.html) (50) and PSIPRED [based on machine learning (ML)] (http://bioinf.cs.ucl.ac.uk/psipred/) (51) were employed to examine the secondary structure of non-mutated and mutated vaccine construct following the default parameters. However, structure was modeled via the Robetta (https://robetta.bakerlab.org/) (52) server based on deep learning methods using RoseTTAFold. These models were enhanced via the GalaxyRefine (https://galaxy.seoklab.org/cgi-bin/submit.cgi?type=REFINE) (53) server, and the most promising enhanced models were further examined for their structure quality validation via PROCHCK (https://saves.mbi.ucla.edu/) (54) and ProSA-web (https://prosa.services.came.sbg.ac.at/prosa.php), which is grounded on the statistical analysis following the available structure (55).

### Identification of discontinuous epitopes

Discontinuous epitopes are crucial for encoding the immune system’s specificity and complexity in responding to infectious agents, leading to more robust and protective immune responses (36). Therefore, the presence of these epitopes within the non-mutated and mutated vaccine was examined via Ellipro (http://tools.iedb.org/ellipro/) (56), which is grounded on geometrical properties of structure, considering the vaccine model structure.

### Docking analysis of vaccine with TLR

Potent vaccines must be able to bind with the receptor to activate an immune activity. Therefore, the formulated vaccine (non-mutated and mutated) was docked with the TLR via the ClusPro (https://cluspro.org/login.php) (57) webserver, which utilized the PIPER docking algorithm following the default parameters, whereas the TLR3 structure was collected via the Protein Data Bank (PDB) (ID: 1ZIW) (https://www.rcsb.org/) database. The obtained docked complexes were examined, and the most potent complexes were selected based on their lowest negative energy, demonstrating strong binding. The binding affinities of complex chosen were computed via the PRODIGY (https://rascar.science.uu.nl/prodigy/) (58) sever, and their interaction was visualized through the PDBsum (https://www.ebi.ac.uk/thornton-srv/databases/pdbsum/) (59) and PyMOL.

### Molecular dynamics simulation

To examine the docked complex’s stability (vaccine with TLR), the Desmond software on an Acer workstation with Ubuntu 20.04 was used (60). The OPLS-2005 Force field was employed to generate the coordinates and topology file of the vaccine and TLR complex to define bonded and non-bonded interactions. The system was prepared, solvated (in the TIP3P model), and further neutralized to mimic the physiological condition via Na+ and Cl− counter ions with 0.15 M salt concentration. Furthermore, the simulations were carried out at 300 K temperature and 1.0325 bar pressure for 100 ns, and the system was minimized and relaxed using the default protocol considering all other criteria that were earlier described (23, 60–63). Furthermore, the trajectory file was examined by root mean square deviation (RMSD) and root mean square fluctuation (RMSF) to evaluate the system’s stability.

### Vaccine-assisted immune activity via immune simulation

The immune activity produced via vaccine (non-mutated and mutated) was analyzed via C-ImmSim (https://kraken.iac.rm.cnr.it/C-IMMSIM/index.php) (64), which employs an ML algorithm. This server assesses the host’s immune activity and the ensuing vaccine administration. Default parameters were used following the adjustment based on previously reported data corresponding to the vaccine construct sequence. Additionally, time steps were modified to reflect the administration of three doses at 1, 84, and 168, with 1,050 set as the simulation step, while all other parameters remained the same (5, 21, 65).

### Optimization and cloning of vaccine

The formulated vaccine (non-mutated and mutated) must have a high expression level for a robust response. Therefore, the constructed sequence was optimized via the VectorBuilder (https://en.vectorbuilder.com/tool/codon-optimization.html) server, considering *E. coli K12* with default parameters. The Codon Adaptation Index (CAI) and GC% should be 0.8–1.0 and 30%–70% for the maximum expression, respectively (25, 66). Furthermore, the optimized sequence was incorporated and cloned in pET-28a (+) via SnapGene (https://www.snapgene.com/) software, considering a specific restriction site as previously reported (5, 7, 11).

## Results

### Collection of the target sequence and their immune assessment

The selected proteins, envelope glycoprotein (Q75008), protease (Q75002), reverse transcriptase (Q75002), and integrase (Q75002), were retrieved from the UniProt database, which is a part of the human immunodeficiency virus type 1 group M subtype C (isolate ETH2220), and are crucial in the infection mechanism (11, 67). The immune assessment of the target sequence demonstrated (**Table 1**) that the required properties can be utilized for vaccine formulation.

**Table 1:** List of selected targets with their immune attributes.

Properties	Envelope	Protease	Reverse T	Integrase
Antigen	0.5425 (Yes)	0.4639 (Yes)	0.5039 (Yes)	0.4628 (Yes)
Allergen	No	No	No	No

### Identification of B-cell epitope and their immune assessment

The crucial B-cell epitope within targets was identified via ABCpred (32) and BepiPred 2.0 (33). Via the ABCpred server, 87 envelope glycoprotein (**Supplementary Table 1**), 9 protease (**Supplementary Table 2**), 56 reverse transcriptase (**Supplementary Table 3**), and 28 integrase (**Supplementary Table 4**) epitopes, and simultaneously via BepiPred, 28 envelope glycoprotein (**Supplementary Table 5**), 4 protease (**Supplementary Table 6**), 20 reverse transcriptase (**Supplementary Table 7**), and 9 integrase (**Supplementary Table 8**) epitopes were predicted. Moreover, 25 envelope glycoprotein, 4 protease, 24 reverse transcriptase, and 13 integrase epitopes were selected to screen out the more precise assessments, which overlapped in both (**Supplementary Table 9**). The immune evaluation of these final epitopes revealed that several epitopes have potential, having antigen, non-allergen, and non-toxic features, and the epitopes with high antigen scores from each target (**Supplementary Table 9**, highlighted in blue) were selected for vaccine formulation as in **Table 2**.

**Table 2:** List of final selected promising LBL epitopes with their immune properties.

Position	Peptide	Antigen	Allergen	Toxic
Envelope glycoprotein				
78–93	PSPQELGLENVTENFN	1.0049 (Yes)	No	No
Protease				
54–69	IKVRQYDQIIIEICGK	0.5430 (Yes)	No	No
Reverse transcriptase				
349–364	LKTGKFAKRGTAHTND	1.1808 (Yes)	No	No
Integrase				
188–203	RGGIGGYSAGERIIDI	0.8048 (Yes)	No	No

### Identification of T-cell (MHC I and MHC II) epitopes and their immune assessment

The MHC I and MHC II epitopes were identified within the targets via Tepitool (40), considering the most frequent alleles (29). The MHC I assessment revealed 238 envelope glycoprotein (**Supplementary Table 10**), 24 protease (**Supplementary Table 11**), 170 reverse transcriptase (**Supplementary Table 12**), and 76 integrase (**Supplementary Table 13**) epitopes. Simultaneously, the MHC II assessment revealed 80 envelope glycoprotein (**Supplementary Table 14**), 12 protease (**Supplementary Table 15**), 61 reverse transcriptase (**Supplementary Table 16**), and 32 integrase (**Supplementary Table 17**) epitopes. The immune assessments of the epitope in MHC I and MHC II revealed several leading immunodominant properties, as shown in **Table 3**. Furthermore, one epitope with many covering alleles and a high antigenic score (**Table 3**) from each respective target was selected for vaccine formulation, as in **Table 4**.

**Table 3:** Immune assessment of MHC I and MHC II epitopes of the targets.

Targets	Total epitopes	Antigen	Non-antigen	Allergen	Non-allergen	Toxic	Non-toxic
MHC I							
Envelope glycoprotein	238	128	110	103	135	1	237
Protease	24	16	8	14	10	0	24
Reverse transcriptase	170	96	74	90	80	0	176
Integrase	76	41	35	44	32	1	75
MHC II							
Envelope glycoprotein	80	48	32	37	43	0	80
Protease	12	6	6	10	2	0	12
Reverse transcriptase	61	38	23	25	36	0	61
Integrase	32	22	10	14	18	0	32

**Table 4:** Selected highly antigenic MHC I and MHC II epitopes within all targets and their immune properties.

Position	Peptide	Alleles	Antigen	Allergen	Toxic
MHC I					
Envelope glycoprotein					
206–214	SLDPIPIHY	HLA-A*30:02 HLA-A*01:01 HLA-B*15:01 HLA-A*32:01 HLA-B*35:01 HLA-A*26:01 HLA-A*11:01 HLA-A*02:06 HLA-B*53:01 HLA-A*03:01 HLA-A*02:01 HLA-B*58:01 HLA-B*44:02 HLA-B*44:03 HLA-A*23:01 HLA-B*57:01	2.0650 (Yes)	No	No
Protease					
91–99	TQLGRTLNF	HLA-B*15:01 HLA-A*32:01 HLA-A*23:01 HLA-A*24:02 HLA-A*30:02 HLA-A*02:06 HLA-B*08:01 HLA-A*26:01	1.3043 (Yes)	No	No
Reverse transcriptase					
381–389	VIWGKTPKF	HLA-A*32:01 HLA-A*23:01 HLA-A*24:02 HLA-B*15:01 HLA-A*26:01 HLA-A*30:02 HLA-B*58:01 HLA-B*57:01 HLA-B*08:01 HLA-B*53:01 HLA-A*02:06	0.4408 (Yes)	No	No
Integrase					
75–83	VAVHVASGY	HLA-A*30:02 HLA-B*35:01 HLA-A*26:01 HLA-B*15:01 HLA-A*01:01 HLA-B*53:01 HLA-B*58:01	0.5921 (Yes)	No	No
MHC II					
Envelope glycoprotein					
351–365	NKTIEFKPSSGGDLE	HLA-DRB1*07:01 HLA-DRB1*15:01 HLA-DRB3*01:01 HLA-DRB3*02:02 HLA-DRB4*01:01 HLA-DRB5*01:01	1.3159 (Yes)	No	No
Protease					
42–56	WKPKMIGGIGGFIKV	HLA-DRB5*01:01	0.6796 (Yes)	No	No
Reverse transcriptase					
343–357	QEPFKNLKTGKFAKR	HLA-DRB1*07:01 HLA-DRB1*15:01 HLA-DRB3*01:01 HLA-DRB3*02:02 HLA-DRB4*01:01 HLA-DRB5*01:01	0.7494 (Yes)	No	No
Integrase					
253–267	DNSDIKVVPRRKAKI	HLA-DRB1*03:01 HLA-DRB1*15:01 HLA-DRB3*02:02 HLA-DRB4*01:01 HLA-DRB5*01:01	1.2710 (Yes)	No	No

### Epitope mapping of B and T cells (MHC I and MHC II) with the variable amino acid

To compute the variability of amino acids across different variants, the total reviewed sequences concerning each target were retrieved from UniProt, and their MSA was accomplished via Clustal Omega (42). The MSA was visualized via the JalView (43) software, which revealed several variable positions across the variant (**Supplementary Figures 1**–**4**). In the case of the B-cell epitope, a total of 38 amino acids from envelope glycoprotein, 29 from protease, 16 from reverse transcriptase, and 9 from integrase were found and mapped (**Supplementary Table 18**) with the selected final epitope (**Table 2**), whereas 73 amino acids from envelope glycoprotein, 8 from protease, 21 from reverse transcriptase, and 12 from integrase for the combined MHC I and II were found and successfully mapped (**Supplementary Tables 19**–**22**) with the selected epitope (**Table 4**, non-mutated vaccine formulation). These mapped amino acids were further incorporated (highlighted in red), and the variability was introduced in the selected non-mutated B- and T-cell epitope (**Tables 2**, **4**). Furthermore, the mutated epitope (**Supplementary Tables 19**–**22**) concerning to non-mutated epitopes were examined for antigen, allergen, and toxicity assessment, similar to those mentioned for non-mutated epitopes, and several potential epitopes were found to have antigenic, non-allergenic, and non-toxic properties (**Supplementary Tables 19**–**22**). Among the potential epitopes, the epitopes with high antigenic scores (**Supplementary Tables 19**–**22**, highlighted in blue) were further selected for mutated vaccine formulation.

### Vaccine formulation and immune and physiological assessments

Among the predicted epitopes, four LBL (**Table 3**), four MHC I, and four MHC II (**Table 4**) were selected based on their high immunodominant activity for the non-mutated vaccine formulation. In contrast, four LBL, four MHC I, and four MHC II mutated epitopes concerning the non-mutated vaccine, based on the introduced variability having high antigenic scores, were used for mutated vaccine formulation, as in **Table 5**. These selected epitopes were joined via EAAAK, AAY, KK, and GPGPG linkers to attain the most immunodominant combination; six distinct non-mutated vaccines were constructed considering the selected epitope and different linkers, adjuvants, and other essential attributes. Moreover, the adjuvant, PADRE, and His-tag were kept as in the N and C terminal end, and the LBL, MHC I, and MHC II were framed in different positions (11, 44) for the vaccine construction, as shown below, and the final constructed sequence was of 276 amino acids.

1. Adjuvant-PADRE-LBL-MHC I-MHC II-His-tag (V1)
2. Adjuvant-PADRE-LBL-MHC II-MHC I-His-tag (V2)
3. Adjuvant-PADRE-MHC(I)-MHC (II)-LBL-His-tag (V3)
4. Adjuvant-PADRE-MHC(II)-MHC (I)-LBL-His-tag (V4)
5. Adjuvant-PADRE-MHC(II)-LBL-MHC (I)-His-tag (V5)
6. Adjuvant-PADRE-MHC(I)-LBL-MHC (II)-His-tag (V6)

**Table 5:** Selected mutated B- and T-cell (MHC I and MHC II) epitopes were mapped with non-mutated epitopes, whereas the mutation was highlighted in blue.

Position	Epitope	A.Pos	R.Pos	V.Amino acid	M.Epitope	Antigen	Allergen	Toxic
B cell								
Envelope glycoprotein								
78–93	PSPQELGLENVTENFN	E86	96	G	PSPQELGL**G**NVTENFN	1.4187 (Yes)	No	No
Protease								
54–69	IKVRQYDQIIIEICGK	I63	63	C	IKVRQYDQI**C**IEICGK	1.1378 (Yes)	No	No
Reverse transcriptase								
349–364	LKTGKFAKRGTAHTND	F354	354	Y	LKTGK**Y**AKRGTAHTND	1.1961 (Yes)	No	No
Integrase								
188–203	RGGIGGYSAGERIIDI	R197	197	R	RGGIGGYSA**R**ERIIDI	1.5032 (Yes)	No	No
MHC I								
Envelope glycoprotein								
206–214	SLDPIPIHY	S206	237	N	**N**LDPIPIHY	2.4487 (Yes)	No	No
Protease								
91–99	TQLGRTLNF	L93	93	I	TQ**I**GRTLNF	1.3254(Yes)	No	No
Reverse transcriptase								
381–389	VIWGKTPKF	T386	387	S	VIWGK**S**PKF	0.5451 (Yes)	No	No
Integrase								
75–83	VAVHVASGY	Y83	83	F	VAVHVASG**F**	0.5744 (Yes)	No	No
MHC II								
Envelope glycoprotein								
351–365	NKTIEFKPSSGGDLE	S359	401	K	NKTIEFKPK**S**GGDLE	1.6779 (Yes)	No	No
Protease								
42–56	WKPKMIGGIGGFIKV	M46	46	I	WKPK**I**IGGIGGFIKV	0.5336(Yes)	No	No
Reverse transcriptase								
343–357	QEPFKNLKTGKFAKR	P345	345	E	QE**E**FKNLKTGKFAKR	0.9871 (Yes)	No	No
Integrase								
253–267	DNSDIKVVPRRKAKI	S255	255	N	DN**N**DIKVVPRRKAKI	1.2852 (Yes)	No	No

A.Pos, Absolute position; R.Pos, Relative position; V.Amino acid, Variable amino acid; M.Epitope, Mutation incorporated based on mapped variability data.

**Figure 2:**
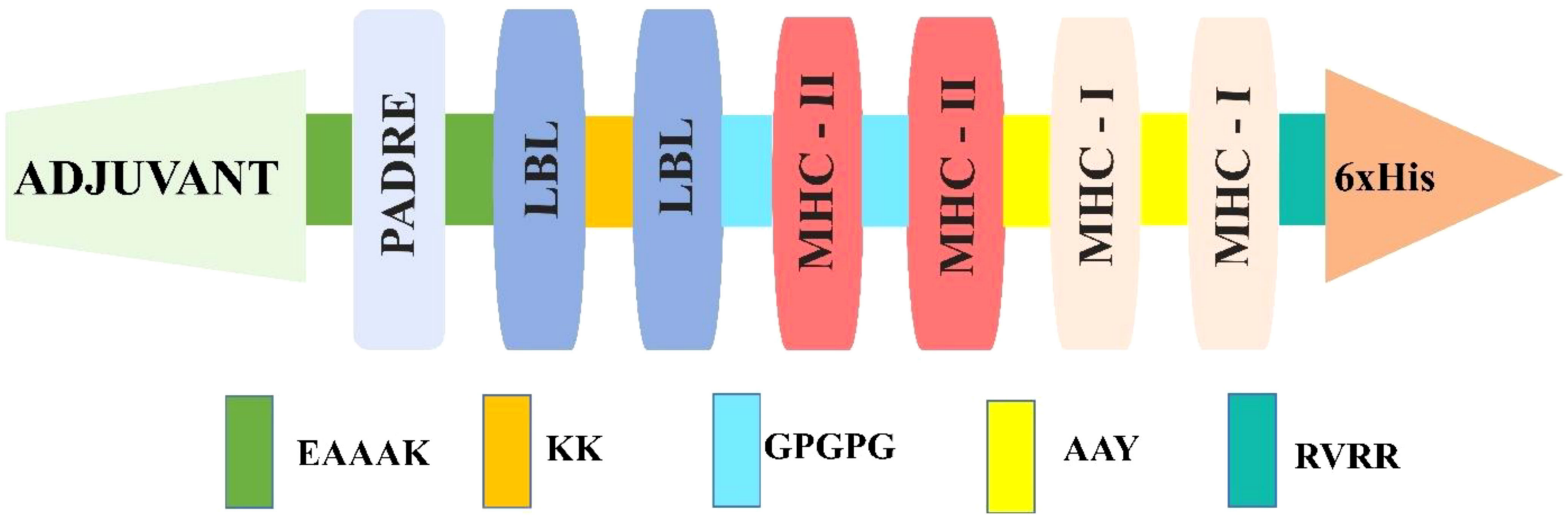
Illustration of vaccine construction followed by different attributes.

Furthermore, antigenicity and allergenicity revealed that the V2 combination was found to have the highest antigenic score among the different combinations, as shown in **Supplementary Table 23**. Moreover, all the constructed vaccines in different forms have an antigenic nature and a non-allergenic feature, which ensures that the selected epitope is highly promising in various forms. These V2 combinations (**Figure 2**) were similarly applied for the mutated vaccine formulation of 276 amino acids, and their antigenicity and allergenicity were analyzed (**Table 6**). Furthermore, the physiochemical properties and solubility analysis revealed suitable properties of non-mutated (**Supplementary Table 24**) and mutated vaccines, as in **Table 6**.

**Table 6:** Computed antigen, allergen, physicochemical, and solubility properties of the mutated vaccine.

Sl. no.	Properties	Mutated vaccine
1.	Antigen	0.8889 (Probable antigen)
2.	Allergen	Non-allergen
3.	Residue count	276
4.	Molecular weight	30,121.02
5.	Theoretical pI	10.16
6.	Formula	C_1348_H_2166_N_408_O_361_S_8_
7.	Estimated half-life	30 h (mammalian reticulocytes, *in vitro*) >20 h (yeast, *in vivo*) >10 h (*Escherichia coli*, *in vivo*)
8.	Instability index	31.76
9.	Aliphatic index	70.43
10.	Grand average of hydropathicity (GRAVY)	−0.593
11.	Solubility	0.674 (Higher than scaled solubility)

### Population coverage analysis of the selected MHC I and MHC II epitopes

**Figure 3:**
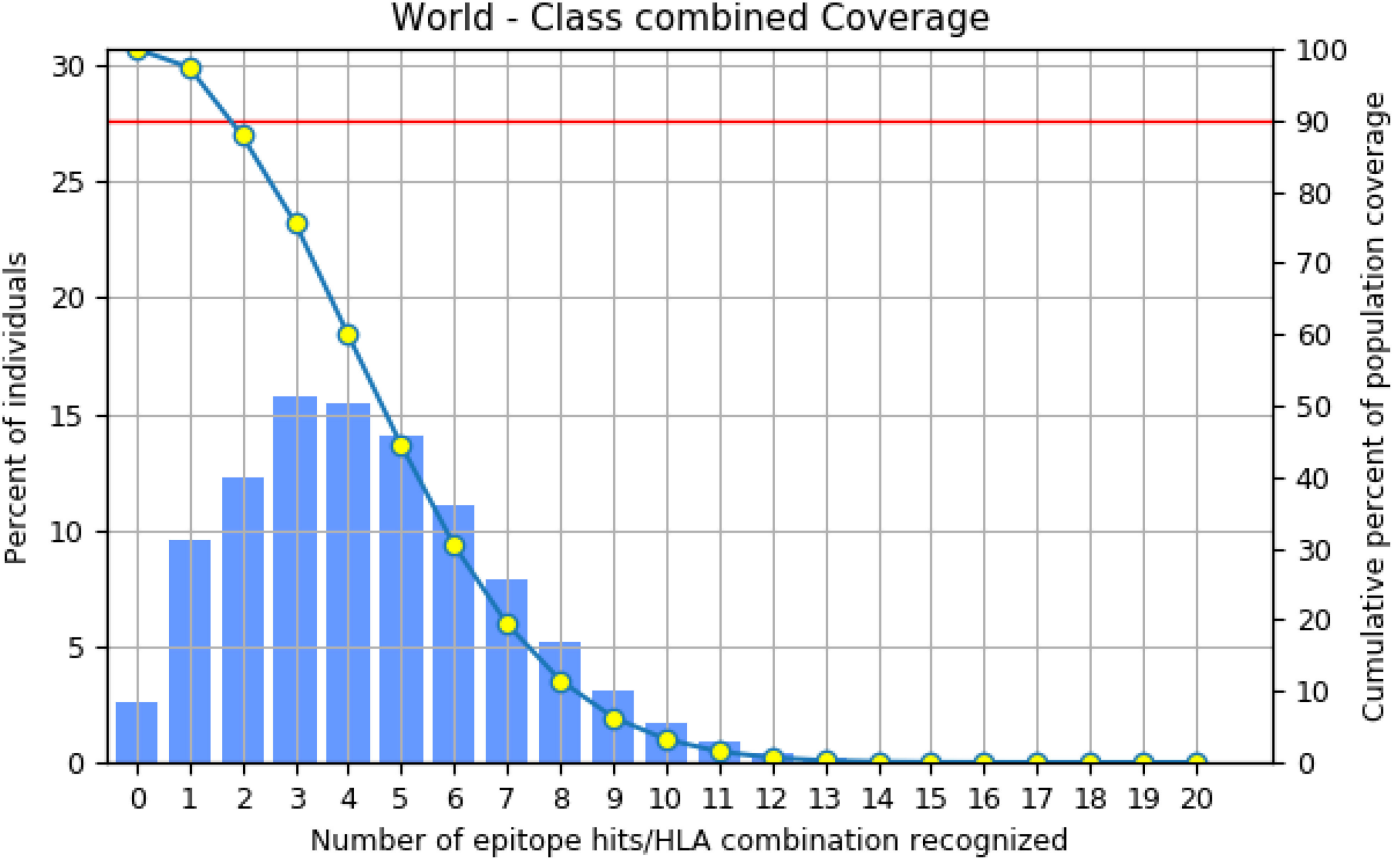
Illustration of selected (MHC I and MHC II) epitope-based population coverage.

For effectiveness, a potent vaccine must have a wide range of coverage (29). These eight epitopes (four MHC I and four MHC II) were examined together, and according to the restricted alleles, there was 97.41% coverage, which shows the broader coverage (**Figure 3**) of the employed epitope in the vaccine formulation.

### Structure modeling and quality assessment

**Figure 4:**
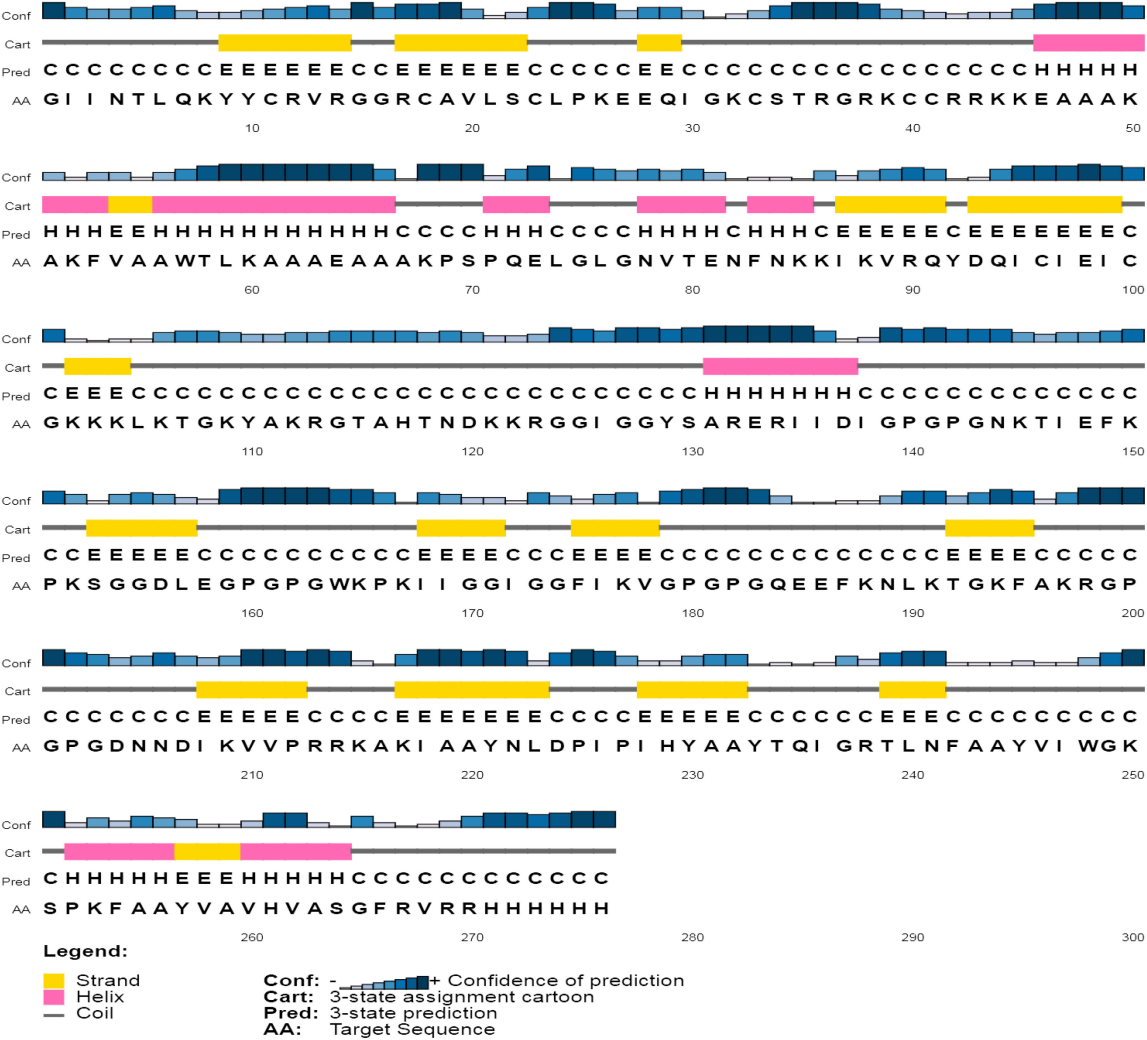
Illustration of secondary composition based on their attributes of the mutated vaccine.

The secondary assessment revealed that the non-mutated vaccine has a helix, 23.91%; strand, 23.91%; and coil, 52.17% (**Supplementary Figure 5**), whereas the mutated has a helix, 20.65%; strand, 25.72%; and coil, 53.62% (**Figure 4**).

**Figure 5:**
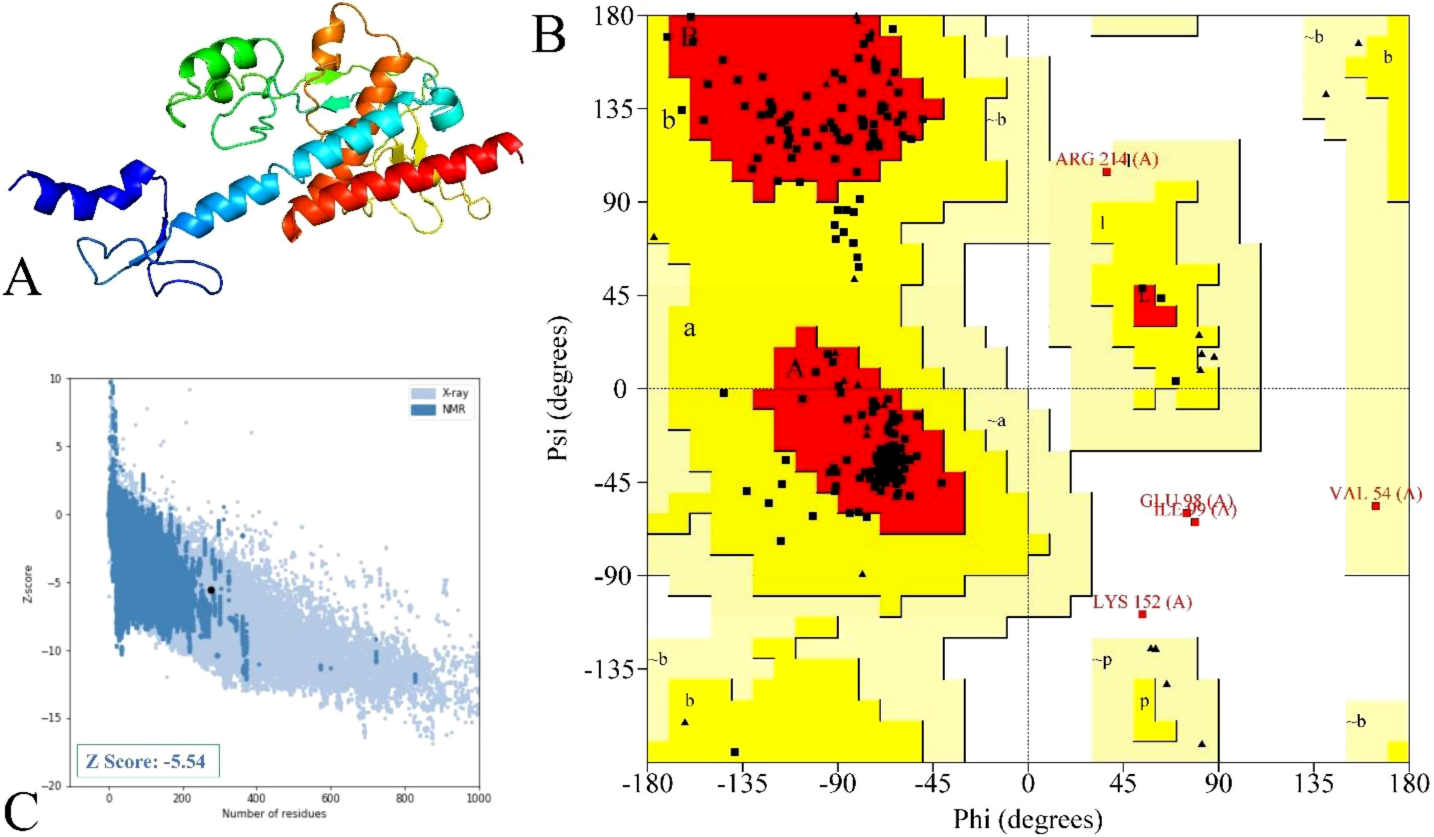
Illustration and modeled mutated vaccine and their quality assessments. **(A)** Designed vaccine model, **(B)** residue representation in various regions, and **(C)** quality evaluation via the *Z*-score value.

The model structure via Robetta (52) servers revealed a confidence score of 0.42 for the non-mutated and 0.41 for the mutated vaccine, which lies within the better-quality range. These models were further refined, and based on their various parameters, model 3 for the non-mutated (**Supplementary Figure 6A**) (**Supplementary Table 25**, highlighted in blue) and model 1 for the mutated vaccine (**Figure 5A**) (**Table 7**, highlighted in blue) were found suitable.

**Table 7:** List of enhanced mutated vaccine models with their attributes.

Model	GDT-HA	RMSD	MolProbity	Clash score	Poor rotamers	Rama favored
Initial	1.0000	0.000	1.466	2.1	0.0	92.0
Model 1	0.9819	0.322	1.805	8.3	0.5	94.9
Model 2	0.9764	0.329	1.884	9.7	0.5	94.5
Model 3	0.9755	0.335	1.897	10.6	0.5	94.9
Model 4	0.9792	0.329	1.912	9.9	0.5	94.2
Model 5	0.9728	0.349	1.918	10.6	0.9	94.5

The structure quality validation via PROCHECK (54) demonstrated that the non-mutated vaccine has 87.3% residue in the most favored region, 8.6% residue in the additional allowed region, 1.8% residue in the generously allowed region, and 2.3% residue in the disallowed region (**Supplementary Figure 6B**), followed by 88.3% residue in the most favored region, 9.5% residue in the additional allowed region, 0.9% residue in the generously allowed region, and 1.4% residue in the disallowed region as in **Figure 5B** for the mutated vaccine. The Ramachandran plot shows that both non-mutated (**Supplementary Figure 6B**) and mutated (**Figure 5B**) vaccine models have only five and three residues in the disallowed regions and are scattered, suggesting less likely to cause significant structural instability. Moreover, most of the residue lies in the favored region, suggesting the overall reliable backbone geometry of the model (22, 68). Furthermore, the *Z*-score assessment done via ProSA-web (55) revealed that the non-mutated vaccine has a −6 score (**Supplementary Figure 6C**) and the mutated vaccine has a −5.54 score (**Figure 5C**); the negative score represents the superior structure model. Based on structural validation, the assessment demonstrated the good quality of the non-mutated and mutated vaccines (22).

### Identification of discontinuous epitopes

The non-mutated and mutated vaccine structure was subjected to the Ellipro (56) server to compute the discontinuous epitope within the vaccine. The subjected non-mutated vaccine revealed that seven epitopes covered 139 amino acids; their range score varied from 0.618 to 0.815 (**Supplementary Table 26**). In contrast, six epitopes were found for the mutated vaccine, covering 147 residues, followed by the score range from 0.588 to 0.967 (**Table 8**). The discontinuous epitopes with both vaccines show that the construct vaccine will lead to a remarkable immune response (69).

**Table 8:** List of obtained discontinuous epitopes within the mutated vaccine.

Sl. No.	Residue	No. of residues	Score
1.	A:F187, A:K188, A:N189, A:L190, A:K191, A:T192, A:G193, A:K194, A:F195	9	0.967
2.	A:G1, A:I2, A:I3, A:N4, A:T5, A:L6, A:Q7, A:K8, A:Y9, A:Y10, A:C11, A:R12, A:V13, A:R14, A:G15, A:G16, A:R17, A:C18, A:A19, A:V20, A:L21, A:S22, A:C23, A:L24, A:P25, A:K26, A:E27, A:E28, A:Q29, A:I30, A:G31, A:K32, A:C33, A:S34, A:T35, A:R36, A:G37, A:R38, A:K39, A:C40, A:C41, A:R42, A:R43	43	0.804
3.	A:P151, A:G154, A:D156, A:L157, A:E158, A:G159, A:P160, A:G161, A:P162, A:G163, A:W164, A:K165, A:P166, A:G179, A:P180, A:G181, A:P182, A:G183, A:Q184, A:E185, A:E186, A:A196, A:K197, A:R198, A:G199, A:P200, A:G201, A:P202, A:G203, A:D204	30	0.689
4.	A:K217, A:A220, A:Y221, A:N222, A:L223, A:D224, A:P225, A:I226, A:P227, A:H229, A:Y230	11	0.648
5.	A:K68, A:P69, A:S70, A:P71, A:Q72, A:E73, A:L74, A:G77, A:N78, A:V79, A:T80, A:E81, A:N82, A:F83, A:K85, A:D137, A:I138, A:G139, A:P140, A:G141, A:P142, A:G143, A:N144, A:K145, A:S264, A:G265, A:R267, A:V268, A:R269, A:R270, A:H271, A:H272, A:H274, A:H275, A:H276	35	0.599
6.	A:C100, A:G101, A:K102, A:K104, A:L105, A:T107, A:G108, A:K109, A:A111, A:K112, A:G114, A:T115, A:A116, A:H117, A:T118, A:N119, A:D120, A:K121, A:K122	19	0.588

### Docking analysis of the non-mutated and mutated vaccine with TLR

**Figure 6:**
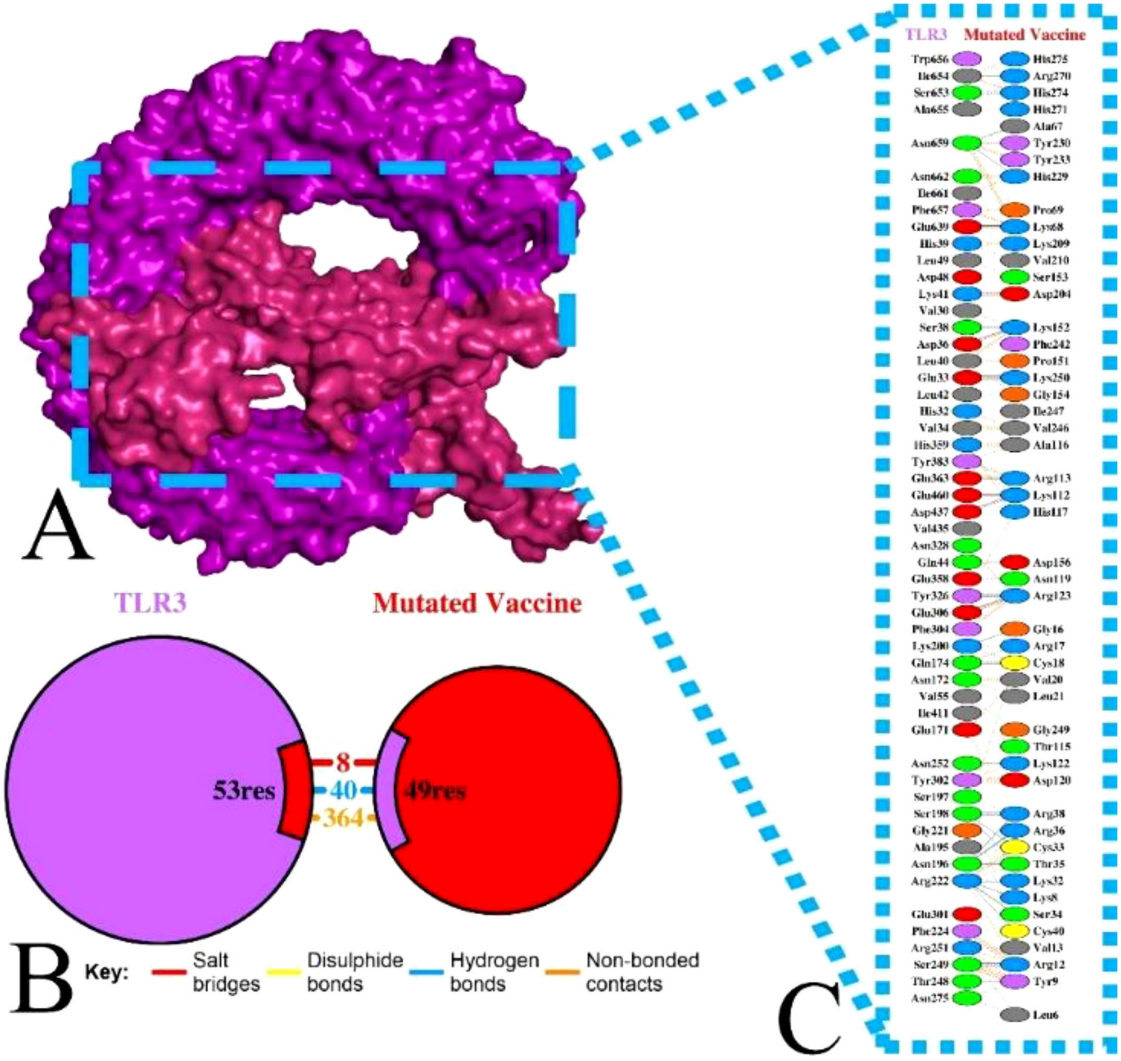
Illustration of TLR3 with mutated vaccine. **(A)** Surface interaction, **(B)** interface residue connection, and **(C)** residual interaction.

The molecular activity of formulated non-mutated and mutated vaccines with the TLR3 was accomplished via ClusPro (7). The TLR3 can recognize double-stranded RNA (dsRNA) and single-stranded RNA (ssRNA) and is also vital in antiviral immune responses. Moreover, its activation stimulates dendritic cell activation mediated by HIV-1, which makes it an ideal target (7, 70). Among the generated multiple docked complexes of subjected TLR3 and vaccine, model 6 for the non-mutated (**Supplementary Table 27**) and model 7 for the mutated vaccine (**Supplementary Table 28**) were found most suitable, having high negative energies of −1,120.2 and −1,275.4 kcal/mol, respectively. The binding affinity of complexes was computed via PRODIGY (58), and the score was obtained at −12.8 kcal/mol (TLR3-Non-mutated) and −24.0 kcal/mol (TLR3-Mutated). These complexes were visualized for their various types of interaction followed by the H bond via PDBSum (59). The TLR3-Non-mutated complex shows 16 H bonds followed by 4 salt bridges and 196 non-bonded contacts as in **Supplementary Figure 7**. In contrast, the TLR3-Mutated vaccine revealed 40 H bonds followed by 8 salt bridges and 364 non-bonded contacts, as in **Figure 6**. Moreover, the interface residue is demonstrated in **Supplementary Figure 7**, **Figure 6**. The docking analysis revealed that the vaccine is strongly bound via molecular connection with TLR3, and the incorporated variability in the epitopes does not affect the interaction; rather, it improves, followed by a high number of hydrogen bonds.

### Molecular dynamics simulation

**Figure 7:**
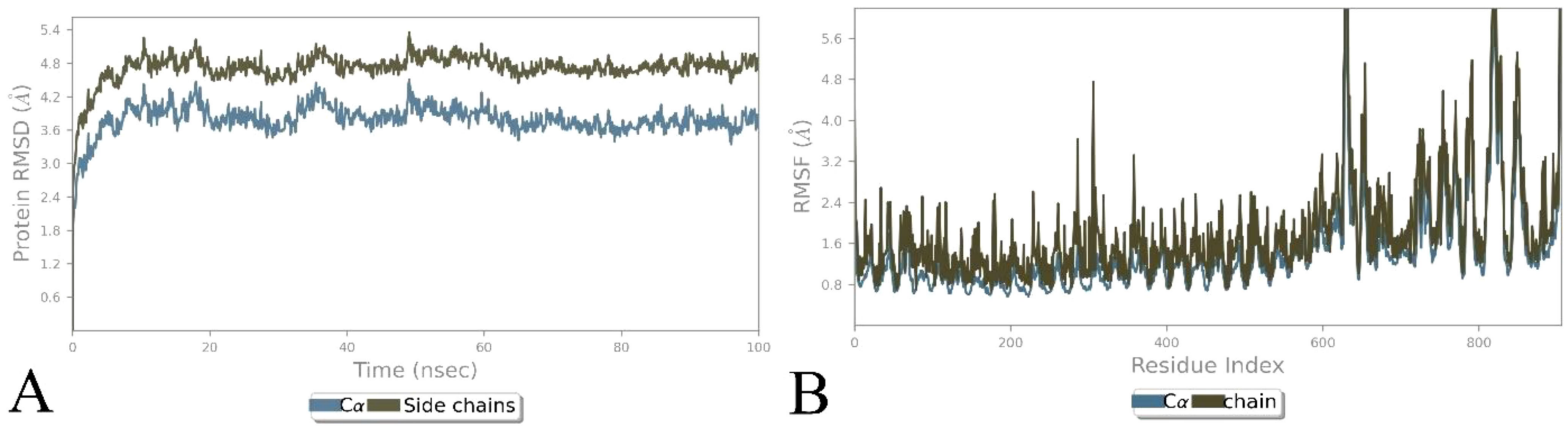
Illustration of simulation-based investigation of the docked complex (mutated vaccine with TLR3). **(A)** The RMSD-based trajectories analysis of the complex, and **(B)** the RMSF-based trajectories analysis of the complex.

The docked TLR3 with the non-mutated and mutated vaccines was analyzed via the Desmond software, followed by considering steps of the parameter (23, 61, 62) to examine their stability. The examination shows that the non-mutated and mutated vaccines remained bound with the TLR3 over the simulation period (**Figure 7**, **Supplementary Figure 8**). The RMSD investigation shows that the Cα of the mutated vaccine–TLR3 complex stabilized after 20 ns, followed by approximately 3.0–3.5 Å deviation, and the side chains were comparably slightly higher at approximately 4.5–5.0 Å, which shows the local conformational adjustments (**Figure 7A**), whereas the non-mutated vaccine–TLR3 complex was gradually stabilized after 20 ns and the Cα atoms rise between 6.0 and 6.5 Å, and the side changes merely followed a similar trend but are slightly higher and stabilized (6.5–7.0 Å) (**Supplementary Figure 8A**). The higher range of RMSD revealed great flexibility, and the complex maintained its structural stability (71, 72). Moreover, the RMSF investigation shows that the alpha of the mutated vaccine–TLR3 complex was less than 2 Å, and the side chain surpassed 4 Å at specific residues, which shows higher fluctuation (**Figure 7B**). In contrast, the alpha of the non-mutated vaccine–TLR3 complex remains below 3 Å, and their side chain was comparably higher with a minor exceeding 6–8 Å at certain regions (**Supplementary Figure 8B**). The minor high peaks in the RMSF of both docked complexes recommend confined rigidity, which is essential for interaction (60, 71, 72).

### Vaccine-assisted immune response activity

**Figure 8:**
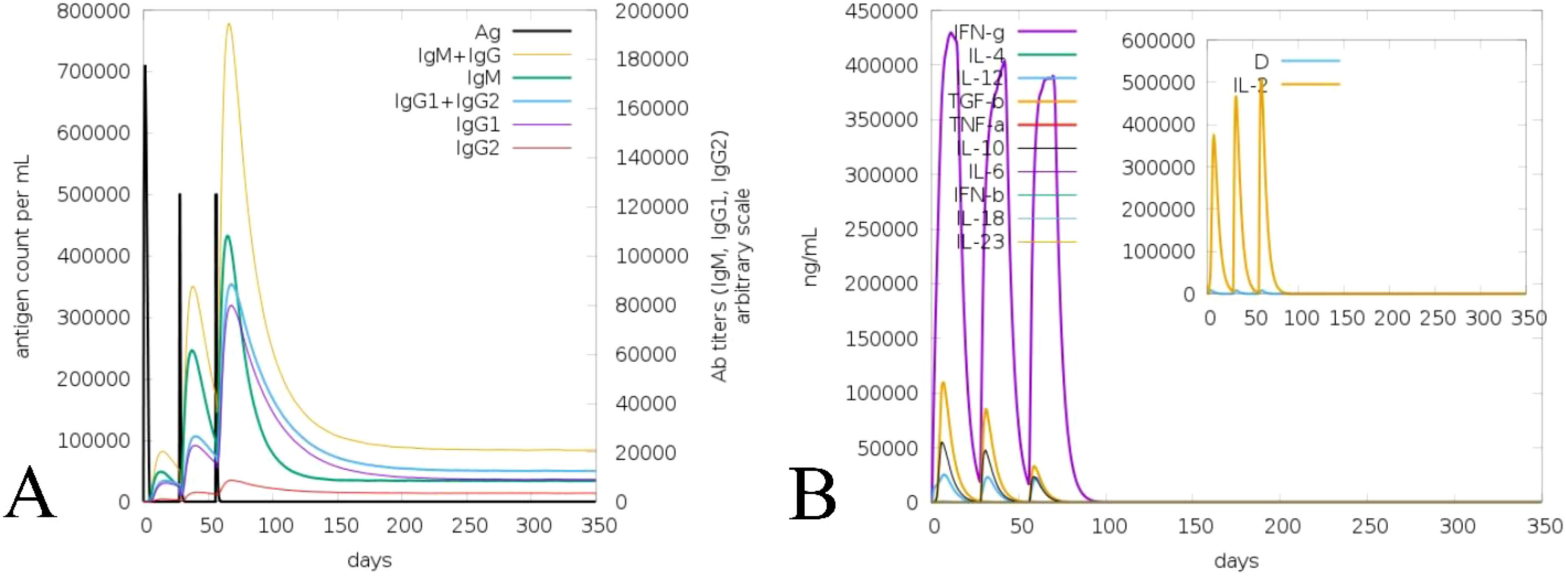
Illustration of immune activity response of the mutated vaccine considering injection steps. **(A)** Vaccine-assisted antigen and antibody level. **(B)** Generated cytokine and interleukin level.

The ML accomplished vaccine immune activity and assisted the C-IMMsim server in considering the time steps of the injection interval, as in **Figure 8** (Mutated) and **Supplementary Figure 9** (Non-mutated). In the case of the non-mutated vaccine, the primary administration shows a high peak of antigen level (700,000 mL) and high generation of immunoglobin, followed by secondary and tertiary administration having an antigen count level of 500,000 each, which further instantly completely reduced, and further, the generated immunoglobins (IgM+IgG, IgM, IgG1+IgG2, IgG1, and IgG2) spiked (650,000) and continued to increase, as shown in **Supplementary Figure 9A**. In contrast, the mutated vaccine shows antigen counts of approximately 700,000, 300,000, and 50,000 per mL at the primary, secondary, and tertiary response levels, respectively. In contrast, the generated immunoglobin level shows a more promising spike (IgM+IgG, IgM, IgG1+IgG2, IgG1, and IgG2) followed by nearly 800,000, which is higher than the non-mutated immunoglobin level as in **Figure 8A**. Moreover, the generated cytokine and interleukins show the highest peaks (IFN-γ, IL-2, IL-4, and TNF-α) at nearly 450,000 ng/mL for non-mutated (**Supplementary Figure 9B**), nearly similar to the mutated vaccine (**Figure 8B**). The repeated exposure of the immunoglobin and cytokine level followed by steps of injection shows that the vaccine is capable of remarkable immune activity in both forms (Mutated, **Figure 8**; and Non-mutated, **Supplementary Figure 9**), and the incorporated variability does not reduce the vaccine’s effectiveness.

### Optimization and cloning of vaccine

**Figure 9:**
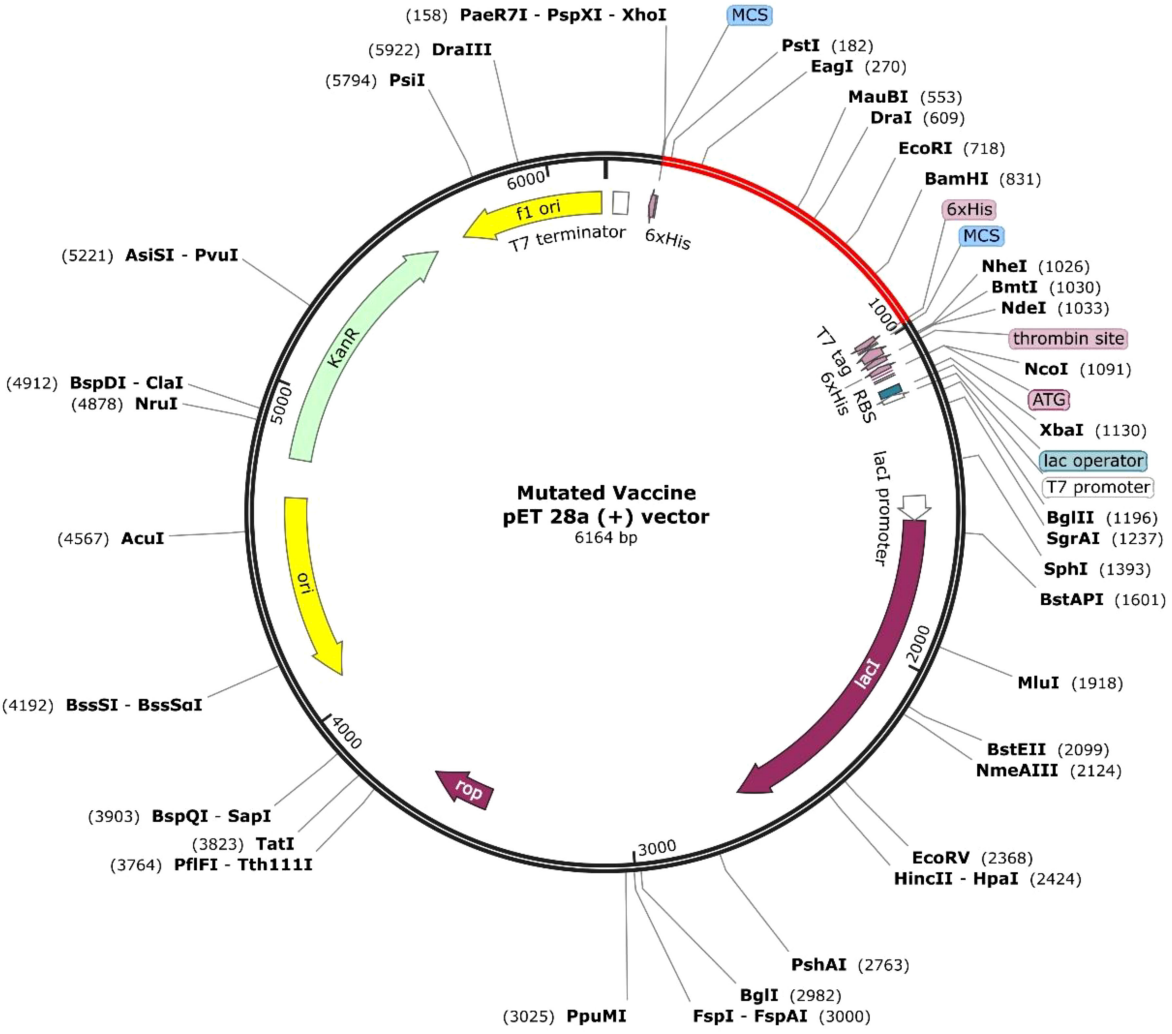
Illustration of the incorporated mutated vaccine in pET28a(+).

The queried non-mutated and mutated vaccine optimized sequence was 831 for each. The CAI value was 0.95 and GC% was 54.27 for the non-mutated vaccine. In contrast, for the mutated vaccine, the CAI was 0.95, and the GC% was 53.43, demonstrating the significant expression in the bacterial system of both vaccines as the obtained value lies in favor of the expression level. Furthermore, the optimized mutated and non-mutated vaccines (red) were cloned in the pET28a (+) vector in **Figure 9**; **Supplementary Figure 10**.

## Discussion

Vaccine formulation for emerging and re-emerging infections presents a promising strategy for effective disease control, offering broad coverage and cost-efficiency. In this context, researchers have leveraged bioinformatics, immunoinformatics, and reverse vaccinology approaches to develop successful multi-epitope vaccines (5, 22, 69, 71). HIV is one of the ongoing endemic concerns due to high infection. No specific vaccine are available to completely eradicate the infection due to its strain variability (26, 28). Previously, researchers applied various approaches for the successful vaccine development towards this infection (5, 11, 21, 70) considering the viral targets mostly from subtype B without incorporating variability in epitopes. In HIV infection, subtype C accounts for the majority of infections, compared to other subtypes, which have not been fully explored yet (13, 14). Therefore, this investigation formulated a potent multi-epitope vaccine by examining subtype C’s four potential targets and incorporating variability (mutation) in epitopes to fight against multiple strains of infection. Based on the antigen, allergen, toxicity, and incorporated variability in the epitopes, four LBL, four MHC I, and four MHC II were found as highly immunodominant epitopes and were selected for the non-mutated and mutated (based on the introduced variability) vaccine formulation. The vaccine’s immune activity was enhanced by including the adjuvant, PADRE, and 6×His-Tag in the construction (5, 21). The antigenicity and allergenicity assessment confirmed that both the mutated and non-mutated vaccines are antigenic, with scores of 0.8889 and 0.7657, and these values are consistent with previous findings (11, 21, 70) and indicate that both vaccines are non-allergenic and the incorporated mutation in the non-mutated vaccine does not compromise its antigenic effectiveness. Furthermore, mutated and non-mutated vaccines’ physiochemical attributes and solubility levels were found suitable and improved (5, 21). The MHC I and MHC II epitopes involved in the vaccine formulation revealed high population coverage, i.e., 97.41%, based on the combined investigation, which is nearly similar to and has improved from the earlier reported study (5, 21, 70). The secondary structural assessment of mutated and non-mutated vaccines showed 20.65% and 23.91% as helix, which is nearly similar to the previous data (5, 11), revealing structural stability. Moreover, the tertiary structure modeling of both mutated and non-mutated vaccines and their validation confirmed that the modeled structures are of favorable quality and closely resemble previously reported data (21, 70). The presence of discontinuous epitopes in vaccines demonstrated their ability to induce protective immunity, as they can produce the antibodies that identify the infection (73, 74). Previously, studies found that the activation of TLR3 can potentially lead to combat HIV infection. Moreover, it can also recognize the dsRNA and ssRNA and initiate the stimulation of dendritic cells facilitated by HIV infection (5, 70). Subsequently, the activation of TLR3 in the viral infection was found to be most suitable, as reported previously by researchers (5, 21, 70, 75). The docking analysis of both non-mutated and mutated vaccine models with TLR3 demonstrated accurate binding, with the incorporated mutation maintaining and enhancing the molecular interaction. This enhancement was reflected in the increased number of interacting residues, with the non-mutated vaccine forming 16 hydrogen bonds with TLR3, while the mutated vaccine formed 40 hydrogen bonds. Furthermore, the binding affinity of both vaccine–TLR3 complexes indicates the favored stability of the system (75). The obtained binding affinity was −12.8 kcal/mol (TLR3-Non-mutated) and −24.0 kcal/mol (TLR3-Mutated). Moreover, nearly similar binding affinities calculated via PRODIGY, i.e., −10.8 kcal/mol (76) and −20.0 kcal/mol (77), were previously reported. Subsequently, a study based on the variability in epitopes reported −20.7 kcal/mol (non-mutated) and −19.5 kcal/mol (mutated) (29). Moreover, Habib et al. found that among the various TLRs (TLR-2, TLR-3, TLR-4, TLR-5, TLR-8, and TLR-9), the designed vaccine exhibited a greater number of interactions towards the TLR-2 followed by 12 H bonds (21). Moreover, exhibited strong interactions specifically with TLR3 and TLR5 among the various TLRs (77). The vaccine-assisted immune simulation activity demonstrated that repeated exposure to formulated vaccines revealed high immunoglobulins and decreased antigen levels. The presence of the IgM in the vaccine will help in the early stage of immune regulation (78), whereas the presence and generation of IgG1 and IgG2 towards the antigens suggest the robust immune response followed by antibody production and neutralization of the viral part (21). Furthermore, the different cytokines and interleukins generated in response to antigens, i.e., IFN-γ (activation of macrophages), IL-2 (stimulates the IFN-γ), IL-4 (B-cell activation), and TNF-α (activation of dendritic cells and T cells), demonstrated the protective immune activity (21, 79, 80) of the formulated non-mutated and mutated vaccine and successfully suppressed and nearly similar to previously reported studies (5, 7, 21), and the introduced mutation does affect and reduce the effectiveness of the production of immune activity. The *in silico* cloning of the non-mutated and mutated vaccine into the pET28a(+) vector within the *E. coli* K12 system demonstrated the maximum expression level, with a CAI value of 0.95 for each and GC% values of 54.27 and 53.43. These values fall within the favored range for optimal expression, aligning closely with previously predicted CAI and GC% values (5, 7, 11, 70). Moreover, the cloning of the designed vaccine into the pET28a(+) vector was deemed suitable for viral infection-based studies owing to its capability to efficiently express viral proteins and the presence of multiple cloning sites, which streamline the cloning process (5, 29, 81).

One of the major hurdles to combating HIV is the low immune response and strain variability. Compared to conventional methods, immunoinformatics-based approaches offer a more precise, rapid, and cost-effective method for vaccine formulation. This study’s major findings demonstrate that the designed vaccine elicits a significant immune response, effectively triggering cellular and humoral activity to combat the infection. Furthermore, based on strain variability, the incorporated mutation does not affect its effectiveness, highlighting its potential to address multi-strain variability. Overall, this study confirms that the formulated vaccines possess immunodominant activity and are capable of effectively fighting HIV infection.

### Limitations and future scope

Strain variability remains a significant challenge in HIV vaccine development. In this study, we successfully designed both non-mutated and mutated vaccine constructs, incorporating epitope variability to address this issue. The vaccines demonstrated remarkable immune activity, highlighting their potential effectiveness. Several steps of investigation and examination were employed via integrating the computational and immunoinformatic approach, which is associated with accuracy and promise. While the formulated vaccine revealed strong immune activity, future steps, including experimental validation, multi-strain efficacy, immune response evaluation, and clinical trials, are essential to ensure its protection and immune activity.

## Conclusion

In this combined mutation-based immunoinformatic investigation, a potent peptide vaccine against HIV infection was successfully formulated by incorporating variability (mutations) in the epitopes utilized in the vaccine. The formulated vaccine effectively evokes a robust immune response based on the fusion of immunodominant epitopes. The docking and dynamics investigation of non-mutated and mutated vaccines with the TLR3 demonstrated strong and stable binding, which ensures the ability of the vaccine activity towards the signaling receptor to trigger the immune response. The vaccine-generated immune response, followed by the injection time step, effectively stimulates immune cells. Additionally, the *in silico*-assisted cloning revealed the high expression levels of non-mutated and mutated vaccines. The strategy employed in this investigation suggests a potent framework for formulating a vaccine capable of addressing strain variability.

## Data Availability

The original contributions presented in the study are included in the article/**Supplementary Material**. Further inquiries can be directed to the corresponding authors.
