# Supplementary material for: An integrated mutation-based immunoinformatic approach incorporating variability in epitopes: a study based on HIV subtype C
Source: Front Immunol. 2025 May 20;16:1540253. doi: 10.3389/fimmu.2025.1540253 (PMC12129944; doi:10.3389/fimmu.2025.1540253)
Supplement: Supplementary file 1 [file DataSheet1.docx]

**Supplementary Table 1.** The B cell epitope within the envelop glycoprotein was identified via ABCPred based on its rank, sequence, position and score.

| Rank | Sequence | Start position | Score |
| --- | --- | --- | --- |
| 1 | TKEIFRPEGGDMRDNW | 453 | 0.98 |
| 2 | ISLWDQGLKPCVKLTP | 108 | 0.94 |
| 3 | KPLGVAPTKPKRRVVE | 481 | 0.91 |
| 3 | TVYYGVPVWKDASPTL | 36 | 0.91 |
| 3 | TFTGTGPCHNVSTVQC | 229 | 0.91 |
| 4 | PEGGDMRDNWRSELYK | 459 | 0.90 |
| 5 | DASPTLFCASDAKAYD | 46 | 0.89 |
| 5 | SIRIGPGQTFYATGDI | 303 | 0.89 |
| 5 | SVEITCTRPSNNTRES | 288 | 0.89 |
| 6 | DAKAYDTEVHNVWGTF | 56 | 0.88 |
| 6 | QIINMWQGVGRAMYAP | 410 | 0.88 |
| 6 | NNSINSANDEMKNCSF | 141 | 0.88 |
| 7 | TAVPWNSSWSNKSQEE | 594 | 0.87 |
| 7 | QMHQDIISLWDQGLKP | 102 | 0.87 |
| 8 | GKLICTTAVPWNSSWS | 588 | 0.86 |
| 8 | GRAMYAPPIEGIIMCR | 419 | 0.86 |
| 8 | CSFNITTELRDKKRKA | 154 | 0.86 |
| 9 | QEEIWDNMTWMQWDRE | 607 | 0.85 |
| 9 | QLLGIWGCSGKLICTT | 579 | 0.85 |
| 9 | IAEGETIIRFENLTNN | 262 | 0.85 |
| 9 | FWMLMICNGMGNLWVT | 21 | 0.85 |
| 10 | NMTWMQWDREISNYTD | 613 | 0.84 |
| 10 | GGEFFYCNTSNLFNST | 374 | 0.84 |
| 11 | PSPQELGLENVTENFN | 78 | 0.83 |
| 11 | DRSIRLVNGFLAIFWD | 731 | 0.83 |
| 11 | DREISNYTDIIYNLLE | 620 | 0.83 |
| 11 | TTHSFNCGGEFFYCNT | 367 | 0.83 |
| 11 | VPLNNGSTDYRLINCN | 179 | 0.83 |
| 12 | HCNISEEKWNKTLQKV | 326 | 0.82 |
| 12 | TGDIIGDIRQAHCNIS | 315 | 0.82 |
| 12 | AIKNNTKVTNNSINSA | 132 | 0.82 |
| 13 | QHMLQLTVWGIKQLQT | 551 | 0.81 |
| 13 | PCVKLTPLCVTLNCNA | 117 | 0.81 |
| 14 | GFLAIFWDDLRSLCLF | 739 | 0.80 |
| 14 | QTLIPHPRGPDRLGGI | 706 | 0.80 |
| 14 | SQNQQDKNEKDLLALD | 637 | 0.80 |
| 14 | EVHNVWGTFACVPTDP | 63 | 0.80 |
| 14 | TDIIYNLLEVSQNQQD | 627 | 0.80 |
| 14 | TSTITQACPKVSLDPI | 195 | 0.80 |
| 15 | EEEGGEQGRDRSIRLV | 722 | 0.79 |
| 15 | SELYKYKVVEIKPLGV | 470 | 0.79 |
| 15 | VMGIQRNCQQWWIWGI | 3 | 0.79 |
| 16 | LLTRDGAKEPHSTKEI | 441 | 0.78 |
| 16 | CTHGIKPVVSTQLLLN | 244 | 0.78 |
| 16 | PCHNVSTVQCTHGIKP | 235 | 0.78 |
| 16 | CPKVSLDPIPIHYCAP | 202 | 0.78 |
| 17 | RFIELIQRIWRAFCNI | 823 | 0.77 |
| 17 | RVRQGYSPLSFQTLIP | 695 | 0.77 |
| 17 | QQWWIWGILGFWMLMI | 11 | 0.77 |
| 18 | DKWENLWNWFNITNWL | 652 | 0.76 |
| 18 | NKTIEFKPSSGGDLEI | 351 | 0.76 |
| 18 | KIIIVQLNESVEITCT | 279 | 0.76 |
| 19 | IVVGEGTDRFIELIQR | 815 | 0.75 |
| 20 | ENFNMWKNDMVEQMHQ | 90 | 0.74 |
| 20 | WFNITNWLWYIKIFIM | 660 | 0.74 |
| 21 | YWGLELKKSAINLLNT | 797 | 0.73 |
| 21 | LKAIEAQQHMLQLTVW | 544 | 0.73 |
| 21 | PIHYCAPAGYAILKCR | 211 | 0.73 |
| 22 | LQTRVLAIERHLRDQQ | 564 | 0.72 |
| 23 | AASITLTVQARQLLSG | 520 | 0.71 |
| 23 | NGMGNLWVTVYYGVPV | 28 | 0.71 |
| 24 | GGVIGLRIIFAVLSIV | 678 | 0.70 |
| 24 | RSNITGLLLTRDGAKE | 434 | 0.70 |
| 25 | GFLGAAGSTMGAASIT | 509 | 0.69 |
| 25 | IEGIIMCRSNITGLLL | 427 | 0.69 |
| 25 | YRLINCNTSTITQACP | 188 | 0.69 |
| 25 | VTLNCNAIKNNTKVTN | 126 | 0.69 |
| 26 | ELLGRSSLKGLQRGWE | 771 | 0.68 |
| 27 | LQRGWETLKYLGSLVQ | 781 | 0.67 |
| 27 | LILIAARTVELLGRSS | 762 | 0.67 |
| 27 | TKPKRRVVEREKRAAL | 488 | 0.67 |
| 28 | NLLNTTAIVVGEGTDR | 808 | 0.66 |
| 28 | TELRDKKRKAYALFYK | 160 | 0.66 |
| 29 | LRSLCLFSYHRLRDLI | 748 | 0.64 |
| 30 | LGALFLGFLGAAGSTM | 503 | 0.63 |
| 31 | NLNITLQCRIKQIINM | 399 | 0.61 |
| 32 | RAFCNIPRRIRQGLEA | 833 | 0.60 |
| 32 | LFNSTKLELFNSSTNL | 385 | 0.60 |
| 33 | LQKVKEKLQKHFPNKT | 338 | 0.59 |
| 33 | ANDEMKNCSFNITTEL | 147 | 0.59 |
| 34 | TVQARQLLSGIVQQQS | 526 | 0.58 |
| 34 | ALFYKLDIVPLNNGST | 171 | 0.58 |
| 35 | GIVQQQSNLLKAIEAQ | 535 | 0.57 |
| 36 | FAVLSIVNRVRQGYSP | 687 | 0.55 |
| 37 | YIKIFIMIVGGVIGLR | 669 | 0.53 |
| 37 | TVWGIKQLQTRVLAIE | 557 | 0.53 |
| 38 | PVVSTQLLLNGSIAEG | 250 | 0.52 |

**Supplementary Table 2.** The B cell epitope within the protease was identified via ABCPred based on its rank, sequence, position and score.

| **Rank** | **Sequence** | **Start position** | **Score** |
| --- | --- | --- | --- |
| 1 | TVLVGPTPVNIIGRNM | 74 | 0.87 |
| 1 | PKMIGGIGGFIKVRQY | 44 | 0.87 |
| 2 | GQLKEALLDTGADDTV | 17 | 0.82 |
| 3 | LEEINLPGKWKPKMIG | 33 | 0.81 |
| 4 | DQIIIEICGKKAIGTV | 60 | 0.80 |
| 5 | VTIKIGGQLKEALLDT | 11 | 0.75 |
| 6 | LDTGADDTVLEEINLP | 24 | 0.72 |
| 7 | IKVRQYDQIIIEICGK | 54 | 0.65 |
| 8 | ITLWQRPLVTIKIGGQ | 3 | 0.61 |

**Supplementary Table 3.** The B cell epitope within the reverse transcriptase was identified via ABCPred based on its rank, sequence, position and score.

| **Rank** | **Sequence** | **Start position** | **Score** |
| --- | --- | --- | --- |
| 1 | EGKISRIGPENPYNTP | 44 | 0.93 |
| 2 | AIKKKDSTKWRKLVDF | 62 | 0.92 |
| 3 | VQPIQLPEKDSWTVND | 241 | 0.91 |
| 4 | HLLKWGFTTPDKKHQK | 208 | 0.90 |
| 5 | LKTGKFAKRGTAHTND | 349 | 0.88 |
| 5 | HQKEPPFLWMGYELHP | 221 | 0.88 |
| 5 | PKVKQWPLTEEKIKAL | 19 | 0.88 |
| 6 | TWEAWWTDYWQATWIP | 397 | 0.87 |
| 7 | NREILKEPVHGVFYDP | 306 | 0.86 |
| 7 | SSMPQILEPFRAPNPE | 162 | 0.86 |
| 8 | HKGIGGNEQVDKLVSS | 539 | 0.85 |
| 8 | QWTFQFYQEPFKNLKT | 336 | 0.85 |
| 9 | FWEVQLGIPHPAGLKK | 87 | 0.84 |
| 9 | QAIQLALQDSGSEVNI | 480 | 0.84 |
| 9 | ESIVIWGKTPKFRLPI | 378 | 0.84 |
| 9 | QKQGNDQWTFQFYQEP | 330 | 0.84 |
| 10 | TVPVKLKPGMDGPKVK | 7 | 0.83 |
| 10 | YWQATWIPEWEFVNTP | 405 | 0.83 |
| 10 | SWTVNDIQKLVGKLNW | 251 | 0.83 |
| 11 | TKIGKAGYVTDRGRQK | 450 | 0.82 |
| 11 | LWMGYELHPDKWTVQP | 228 | 0.82 |
| 11 | PSTNNETPGIRYQYNV | 133 | 0.82 |
| 12 | ASQIYPGIKVRQLCKL | 267 | 0.81 |
| 12 | PEIVIYQYMDDLYVGS | 176 | 0.81 |
| 13 | ENPYNTPVFAIKKKDS | 53 | 0.80 |
| 14 | IILAQPDKSESEIVNQ | 505 | 0.79 |
| 15 | GIPHPAGLKKKKSVTV | 93 | 0.78 |
| 15 | EFVNTPPLVKLWYQLE | 415 | 0.78 |
| 16 | DLEIGQHRAPIEELRE | 192 | 0.77 |
| 16 | GSPPIFQSSMPQILEP | 155 | 0.77 |
| 17 | STKWRKLVDFRELNKR | 68 | 0.74 |
| 17 | YQLEKEPIAGVETFYV | 427 | 0.74 |
| 18 | AVVQKIALESIVIWGK | 370 | 0.73 |
| 18 | KDLIAEIQKQGNDQWT | 323 | 0.73 |
| 18 | GVFYDPSKDLIAEIQK | 316 | 0.73 |
| 19 | DLYVGSDLEIGQHRAP | 186 | 0.72 |
| 19 | VPLDEGFRKYTAFTIP | 118 | 0.72 |
| 20 | EQLISKERVYLSWVPA | 523 | 0.71 |
| 20 | TDRGRQKIVSLTETTN | 459 | 0.71 |
| 20 | GAANRETKIGKAGYVT | 444 | 0.71 |
| 20 | EEAELELAENREILKE | 297 | 0.71 |
| 20 | GIRYQYNVLPQGWKGS | 141 | 0.71 |
| 21 | ALTDIVTLTEEAELEL | 288 | 0.70 |
| 21 | KVRQLCKLLRGAKALT | 275 | 0.70 |
| 22 | IVSLTETTNQKTELQA | 466 | 0.69 |
| 23 | KPGMDGPKVKQWPLTE | 13 | 0.68 |
| 24 | LLRGAKALTDIVTLTE | 282 | 0.67 |
| 24 | PLTEEKIKALTAICEE | 25 | 0.67 |
| 25 | NEQVDKLVSSGIRKVL | 545 | 0.66 |
| 25 | PIAGVETFYVDGAANR | 433 | 0.66 |
| 25 | KKSVTVLDVGDAYFSV | 103 | 0.66 |
| 26 | AKRGTAHTNDVKQLTA | 355 | 0.64 |
| 27 | LEPFRAPNPEIVIYQY | 168 | 0.63 |
| 28 | RELNKRTQDFWEVQLG | 78 | 0.60 |
| 29 | GSEVNIVTDSQYALGI | 490 | 0.58 |
| 30 | DVKQLTAVVQKIALES | 364 | 0.51 |

**Supplementary Table 4.** The B cell epitope within the integrase was identified via ABCPred based on its rank, sequence, position and score.

| **Rank** | **Sequence** | **Start position** | **Score** |
| --- | --- | --- | --- |
| 1 | AKIIRDYGKQMAGADC | 265 | 0.95 |
| 2 | EEHEKYHSNWRAMANE | 10 | 0.94 |
| 3 | YGKQMAGADCVAGRQD | 271 | 0.93 |
| 4 | PGIWQLDCTHLEGKII | 58 | 0.92 |
| 4 | CQLKGEAIHGQVNCSP | 43 | 0.92 |
| 5 | ASDIQTKELQNQILKI | 205 | 0.90 |
| 6 | VRVIHTDNGSNFTSNA | 110 | 0.89 |
| 7 | PKEIVACCDKCQLKGE | 33 | 0.88 |
| 7 | GERIIDIIASDIQTKE | 197 | 0.88 |
| 8 | CWWAGIQQEFGIPYNP | 130 | 0.86 |
| 9 | AIHGQVNCSPGIWQLD | 49 | 0.85 |
| 10 | AETGQETAYFLLKLAG | 91 | 0.83 |
| 10 | SNWRAMANEFNIPPVV | 17 | 0.83 |
| 11 | PQSQGVVESMNKELKK | 145 | 0.82 |
| 12 | EGAVVIQDNSDIKVVP | 246 | 0.80 |
| 12 | LKIQNFRVYYRDSRDP | 218 | 0.80 |
| 13 | LDGIDKAQEEHEKYHS | 2 | 0.79 |
| 13 | RGGIGGYSAGERIIDI | 188 | 0.79 |
| 14 | YRDSRDPIWKGPAKLL | 227 | 0.78 |
| 14 | KLAGRWPVRVIHTDNG | 103 | 0.78 |
| 15 | NGSNFTSNAVKAACWW | 117 | 0.77 |
| 16 | SGYIEAEVIPAETGQE | 81 | 0.74 |
| 17 | AKLLWKGEGAVVIQDN | 239 | 0.73 |
| 17 | LKKIIGQVREQAEHLK | 158 | 0.73 |
| 17 | QEFGIPYNPQSQGVVE | 137 | 0.73 |
| 18 | QDNSDIKVVPRRKAKI | 252 | 0.70 |
| 19 | EGKIILVAVHVASGYI | 69 | 0.68 |
| 20 | AEHLKTAVQMAVFIHN | 169 | 0.64 |

**Supplementary Table 5.** The B cell epitope within the envelope glycoprotein was identified via Bepipred based on its position, sequence, and length.

| **No.** | **Start** | **End** | **Peptide** | **Length** |
| --- | --- | --- | --- | --- |
| 1 | 5 | 11 | GIQRNCQ | 7 |
| 2 | 44 | 46 | WKD | 3 |
| 3 | 58 | 64 | KAYDTEV | 7 |
| 4 | 78 | 81 | PSPQ | 4 |
| 5 | 85 | 98 | LENVTENFNMWKND | 14 |
| 6 | 112 | 113 | DQ | 2 |
| 7 | 116 | 118 | KPC | 3 |
| 8 | 132 | 150 | AIKNNTKVTNNSINSANDE | 19 |
| 9 | 161 | 185 | ELRDKKRKAYALFYKLDIVPLNNGS | 25 |
| 10 | 196 | 208 | STITQACPKVSLD | 13 |
| 11 | 225 | 233 | CRDKTFTGT | 9 |
| 12 | 238 | 245 | NVSTVQCT | 8 |
| 13 | 261 | 265 | SIAEG | 5 |
| 14 | 273 | 278 | NLTNNA | 6 |
| 15 | 297 | 302 | SNNTRE | 6 |
| 16 | 317 | 322 | DIIGDI | 6 |
| 17 | 335 | 336 | NK | 2 |
| 18 | 347 | 365 | KHFPNKTIEFKPSSGGDLE | 19 |
| 19 | 389 | 400 | TKLELFNSSTNL | 12 |
| 20 | 409 | 418 | KQIINMWQGV | 10 |
| 21 | 420 | 429 | RAMYAPPIEG | 10 |
| 22 | 447 | 498 | AKEPHSTKEIFRPEGGDMRDNWRSELYKYKVVEIKPLGVAPTKPKRRVVERE | 52 |
| 23 | 548 | 562 | EAQQHMLQLTVWGIK | 15 |
| 24 | 575 | 581 | LRDQQLL | 7 |
| 25 | 597 | 614 | PWNSSWSNKSQEEIWDNM | 18 |
| 26 | 622 | 625 | EISN | 4 |
| 27 | 642 | 656 | DKNEKDLLALDKWEN | 15 |
| 28 | 697 | 739 | RQGYSPLSFQTLIPHPRGPDRLGGIEEEGGEQGRDRSIRLVNG | 43 |

**Supplementary Table 6.** The B cell epitope within the protease was identified via Bepipred based on its position, sequence, and length.

| **No.** | **Start** | **End** | **Peptide** | **Length** |
| --- | --- | --- | --- | --- |
| 1 | 5 | 8 | LWQR | 4 |
| 2 | 24 | 42 | LDTGADDTVLEEINLPGKW | 19 |
| 3 | 57 | 59 | RQY | 3 |
| 4 | 90 | 95 | LTQLGR | 6 |

**Supplementary Table 7.** The B cell epitope within the reverse transcriptase was identified via Bepipred based on its position, sequence, and length.

| **No.** | **Start** | **End** | **Peptide** | **Length** |
| --- | --- | --- | --- | --- |
| 1 | 16 | 35 | MDGPKVKQWPLTEEKIKALT | 20 |
| 2 | 46 | 55 | KISRIGPENP | 10 |
| 3 | 65 | 66 | KK | 2 |
| 4 | 68 | 68 | S | 1 |
| 5 | 81 | 91 | NKRTQDFWEVQ | 11 |
| 6 | 134 | 140 | STNNETP | 7 |
| 7 | 173 | 173 | A | 1 |
| 8 | 194 | 203 | EIGQHRAPIE | 10 |
| 9 | 214 | 233 | FTTPDKKHQKEPPFLWMGYE | 20 |
| 10 | 239 | 256 | WTVQPIQLPEKDSWTVND | 18 |
| 11 | 273 | 274 | GI | 2 |
| 12 | 285 | 292 | GAKALTDI | 8 |
| 13 | 308 | 308 | E | 1 |
| 14 | 346 | 346 | F | 1 |
| 15 | 355 | 364 | AKRGTAHTND | 10 |
| 16 | 394 | 435 | QKETWEAWWTDYWQATWIPEWEFVNTPPLVKLWYQLEKEPIA | 42 |
| 17 | 447 | 450 | NRET | 4 |
| 18 | 464 | 473 | QKIVSLTETT | 10 |
| 19 | 510 | 517 | PDKSESEI | 8 |
| 20 | 538 | 550 | AHKGIGGNEQVDK | 13 |

**Supplementary Table 8.** The B cell epitope within the integrase was identified via Bepipred based on its position, sequence, and length.

| **No.** | **Start** | **End** | **Peptide** | **Length** |
| --- | --- | --- | --- | --- |
| 1 | 5 | 31 | IDKAQEEHEKYHSNWRAMANEFNIPPV | 27 |
| 2 | 43 | 57 | CQLKGEAIHGQVNCS | 15 |
| 3 | 91 | 96 | AETGQE | 6 |
| 4 | 118 | 124 | GSNFTSN | 7 |
| 5 | 140 | 148 | GIPYNPQSQ | 9 |
| 6 | 160 | 160 | K | 1 |
| 7 | 162 | 172 | IGQVREQAEHL | 11 |
| 8 | 185 | 238 | FKRRGGIGGYSAGERIIDIIASDIQTKELQNQILKIQNFRVYYRDSRDPIWKGP | 54 |
| 9 | 256 | 285 | DIKVVPRRKAKIIRDYGKQMAGADCVAGRQ | 30 |

**Supplementary Table 9.** List of B cell epitopes from ABCpred server of envelop glycoprotein protease, reverse transcriptase, and integrase, which overlaps in BepiPred server with their antigen, allergen, and toxicity features.

| **Sl.No** | **Position** | **Peptide** | **Antigen** | **Allergen** | **Toxic** |
| --- | --- | --- | --- | --- | --- |
| **Envelope glycoprotein** | | | | | |
|  | 3-18 | VMGIQRNCQQWWIWGI | 0.3190 (No) | Yes | No |
|  | 36-51 | TVYYGVPVWKDASPTL | 0.3930 (No) | Yes | No |
|  | 56-71 | DAKAYDTEVHNVWGTF | 0.4435 (Yes) | Yes | No |
|  | **78-93** | **PSPQELGLENVTENFN** | **1.0049 (Yes)** | **No** | **No** |
|  | 102-117 | QMHQDIISLWDQGLKP | 0.0470 (Yes) | No | No |
|  | 108-123 | ISLWDQGLKPCVKLTP | 0.6352 (Yes) | Yes | No |
|  | 132-147 | AIKNNTKVTNNSINSA | 0.6492 (Yes) | No | No |
|  | 160-175 | TELRDKKRKAYALFYK | 0.8777 (Yes) | No | No |
|  | 195-210 | TSTITQACPKVSLDPI | 0.7936 (Yes) | Yes | No |
|  | 235-250 | PCHNVSTVQCTHGIKP | 0.7427 (Yes) | No | No |
|  | 250-265 | PVVSTQLLLNGSIAEG | 0.2215 (No) | Yes | No |
|  | 288-303 | SVEITCTRPSNNTRES | 1.4972 (Yes) | Yes | No |
|  | 315-330 | TGDIIGDIRQAHCNIS | 0.4510 (Yes) | Yes | No |
|  | 326-341 | HCNISEEKWNKTLQKV | 0.0098 (No) | No | No |
|  | 385-400 | LFNSTKLELFNSSTNL | 0.1886 (No) | No | No |
|  | 419-434 | GRAMYAPPIEGIIMCR | 0.4994 (Yes) | Yes | No |
|  | 453-468 | TKEIFRPEGGDMRDNW | 0.2465 (No) | Yes | No |
|  | 459-474 | PEGGDMRDNWRSELYK | 0.1539 (No) | Yes | No |
|  | 470-485 | SELYKYKVVEIKPLGV | 0.7067 (Yes) | Yes | No |
|  | 481-496 | KPLGVAPTKPKRRVVE | 0.9972 (Yes) | No | No |
|  | 544-559 | LKAIEAQQHMLQLTVW | 0.2818 (No) | Yes | No |
|  | 613-628 | NMTWMQWDREISNYTD | 0.7660 (Yes) | Yes | No |
|  | 620-635 | DREISNYTDIIYNLLE | 0.2275 (No) | Yes | No |
|  | 706-721 | QTLIPHPRGPDRLGGI | 0.5847 (Yes) | No | No |
|  | 722-737 | EEEGGEQGRDRSIRLV | 0.6331 (Yes) | Yes | No |
| **Protease** | | | | | |
|  | 3-18 | ITLWQRPLVTIKIGGQ | 0.4649 (Yes) | Yes | No |
|  | 24-39 | LDTGADDTVLEEINLP | 0.1973 (No) | No | No |
|  | 44-59 | PKMIGGIGGFIKVRQY | 0.5071 (Yes) | No | No |
|  | **54-69** | **IKVRQYDQIIIEICGK** | **0.5430 (Yes)** | **No** | **No** |
| **Reverse transcriptase** | | | | | |
|  | 19-34 | PKVKQWPLTEEKIKAL | 0.7642 (Yes) | Yes | No |
|  | 44-59 | EGKISRIGPENPYNTP | 0.5148 (Yes) | Yes | No |
|  | 53-68 | ENPYNTPVFAIKKKDS | 0.9149 (Yes) | No | No |
|  | 62-77 | AIKKKDSTKWRKLVDF | 0.4895 (Yes) | No | No |
|  | 68-83 | STKWRKLVDFRELNKR | 0.5827 (Yes) | No | No |
|  | 78-93 | RELNKRTQDFWEVQLG | 0.9605 (Yes) | No | No |
|  | 133-148 | PSTNNETPGIRYQYNV | 0.6855 (Yes) | No | No |
|  | 162-177 | SSMPQILEPFRAPNPE | -0.0777 (No) | No | No |
|  | 168-183 | LEPFRAPNPEIVIYQY | 0.2014 (No) | No | No |
|  | 192-207 | DLEIGQHRAPIEELRE | 0.6141 (Yes) | No | No |
|  | 241-256 | VQPIQLPEKDSWTVND | 0.8192 (Yes) | No | No |
|  | 267-282 | ASQIYPGIKVRQLCKL | 1.2976 (Yes) | Yes | No |
|  | 282-297 | LLRGAKALTDIVTLTE | 0.2196 (No) | No | No |
|  | 297-312 | EEAELELAENREILKE | 0.1340 (No) | Yes | No |
|  | 306-321 | NREILKEPVHGVFYDP | -0.5697 (No) | Yes | No |
|  | 336-351 | QWTFQFYQEPFKNLKT | 0.2750 (No) | No | No |
|  | **349-364** | **LKTGKFAKRGTAHTND** | **1.1808 (Yes)** | **No** | **No** |
|  | 355-370 | AKRGTAHTNDVKQLTA | 0.4571 (Yes) | No | No |
|  | 397-412 | TWEAWWTDYWQATWIP | 0.1327 (No) | No | No |
|  | 405-420 | YWQATWIPEWEFVNTP | 0.5241 (Yes) | Yes | No |
|  | 415-430 | EFVNTPPLVKLWYQLE | 0.1956 (No) | Yes | No |
|  | 444-459 | GAANRETKIGKAGYVT | 1.4064 (Yes) | Yes | No |
|  | 459-474 | TDRGRQKIVSLTETTN | 1.0082 (Yes) | No | No |
|  | 505-520 | IILAQPDKSESEIVNQ | 0.2802 (No) | Yes | No |
| **Integrase** | | | | | |
|  | 10-25 | EEHEKYHSNWRAMANE | 0.2934 (No) | Yes | No |
|  | 43-58 | CQLKGEAIHGQVNCSP | 0.7653 (Yes) | Yes | No |
|  | 91-106 | AETGQETAYFLLKLAG | 0.4856 (Yes) | Yes | No |
|  | 110-125 | VRVIHTDNGSNFTSNA | 0.3534 (No) | No | No |
|  | 117-132 | NGSNFTSNAVKAACWW | 0.5345 (Yes) | No | No |
|  | 137-152 | QEFGIPYNPQSQGVVE | 0.7360 (Yes) | Yes | No |
|  | 145-160 | PQSQGVVESMNKELKK | 0.2573 (No) | Yes | No |
|  | 158-173 | LKKIIGQVREQAEHLK | 0.0307 (No) | No | No |
|  | **188-203** | **RGGIGGYSAGERIIDI** | **0.8048 (Yes)** | **No** | **No** |
|  | 197-212 | GERIIDIIASDIQTKE | 0.6835 (Yes) | No | No |
|  | 205-220 | ASDIQTKELQNQILKI | 0.2985 (No) | No | No |
|  | 218-233 | LKIQNFRVYYRDSRDP | 0.7924 (Yes) | No | No |
|  | 265-280 | AKIIRDYGKQMAGADC | -0.5678 (No) | No | Yes |

**Supplementary Table 10.** Identified MHC-I epitope within envelope glycoprotein, rank, alleles, antigen, allergen, and toxic properties.

| **Sl.No** | **Position** | **Peptide** | **Rank** | **Alleles** | **Antigen** | **Allergen** | **Toxic** |
| --- | --- | --- | --- | --- | --- | --- | --- |
|  | 2-10 | KVMGIQRNC | 0.69 | HLA-A*30:01 | 0.2534 (No) | No | No |
|  | 5-13 | GIQRNCQQW | 0.54 | HLA-B*58:01  HLA-A*32:01  HLA-B*57:01 | -0.1804 (No) | Yes | No |
|  | 6-14 | IQRNCQQWW | 0.6 | HLA-B*57:01  HLA-B*58:01  HLA-A*32:01 | -0.5199 (No) | No | No |
|  | 8-16 | RNCQQWWIW | 0.5 | HLA-B*58:01  HLA-B*57:01  HLA-A*32:01 | -0.3750 (No) | No | Yes |
|  | 13-21 | WWIWGILGF | 0.47 | HLA-A*23:01  HLA-A*24:02 | 1.6139 (Yes) | No | No |
|  | 26-34 | ICNGMGNLW | 0.08 | HLA-B*58:01  HLA-B*57:01  HLA-B*53:01 | -0.4459 (No) | No | No |
|  | 29-37 | GMGNLWVTV | 0.46 | HLA-A*02:01  HLA-A*02:03 | -0.0390 (No) | Yes | No |
|  | 30-38 | MGNLWVTVY | 0.31 | HLA-B*35:01  HLA-A*30:02  HLA-B*15:01 | -0.3967 (No) | Yes | No |
|  | 31-39 | GNLWVTVYY | 0.46 | HLA-A*30:02 | -0.4341 (No) | Yes | No |
|  | 35-43 | VTVYYGVPV | 0.47 | HLA-A*68:02 | 0.3034 (No) | No | No |
|  | 36-44 | TVYYGVPVW | 0.02 | HLA-A*32:01  HLA-B*58:01  HLA-B*53:01  HLA-B*57:01  HLA-A*26:01  HLA-A*23:01  HLA-B*35:01 | -0.0750 (No) | No | No |
|  | 37-45 | VYYGVPVWK | 0.21 | HLA-A*30:01  HLA-A*31:01  HLA-A*33:01  HLA-A*03:01 | 0.3747 (No) | No | No |
|  | 43-51 | VWKDASPTL | 0.09 | HLA-A*24:02  HLA-A*23:01  HLA-B*08:01  HLA-A*30:01 | 0.1969 (No) | No | No |
|  | 46-54 | DASPTLFCA | 0.61 | HLA-A*68:02  HLA-B*51:01 | 1.0706 (Yes) | Yes | No |
|  | 50-58 | TLFCASDAK | 0.95 | HLA-A*11:01 | 1.1092 (Yes) | Yes | No |
|  | 52-60 | FCASDAKAY | 0.19 | HLA-B*35:01  HLA-A*01:01 | 0.8015 (Yes) | Yes | No |
|  | 56-64 | DAKAYDTEV | 0.08 | HLA-B*51:01 | 0.5307 (Yes) | No | No |
|  | 59-67 | AYDTEVHNV | 0.34 | HLA-A*24:02  HLA-A*23:01 | 0.4509 (Yes) | No | No |
|  | 60-68 | YDTEVHNVW | 0.15 | HLA-B*44:02  HLA-B*44:03  HLA-B*53:01  HLA-B*58:01  HLA-B*57:01 | 0.2940 (No) | No | No |
|  | 63-71 | EVHNVWGTF | 0.02 | HLA-A*26:01  HLA-B*35:01  HLA-B*53:01  HLA-A*68:02 | 0.5756 (Yes) | Yes | No |
|  | 66-74 | NVWGTFACV | 0.2 | HLA-A*02:06  HLA-A*02:01 | 0.0905 (No) | No | No |
|  | 75-83 | PTDPSPQEL | 0.27 | HLA-A*01:01 | 1.0359 (Yes) | Yes | No |
|  | 77-85 | DPSPQELGL | 0.26 | HLA-B*53:01  HLA-B*51:01  HLA-B*35:01  HLA-B*07:02 | 1.9488 (Yes) | No | No |
|  | 87-95 | NVTENFNMW | 0.09 | HLA-B*53:01  HLA-B*58:01  HLA-A*26:01  HLA-B*57:01  HLA-A*32:01 | 0.1018 (No) | Yes | No |
|  | 88-96 | VTENFNMWK | 0.42 | HLA-A*11:01 | -0.6031 (No) | Yes | No |
|  | 100-108 | VEQMHQDII | 0.44 | HLA-B*40:01 | 0.0046 (No) | No | No |
|  | 102-110 | QMHQDIISL | 0.13 | HLA-A*02:03  HLA-A*02:01  HLA-B*08:01  HLA-A*02:06  HLA-A*32:01  HLA-B*15:01 | 0.1217 (No) | No | No |
|  | 103-111 | MHQDIISLW | 0.15 | HLA-A*23:01  HLA-B*53:01  HLA-A*24:02  HLA-B*58:01  HLA-B*57:01  HLA-A*32:01  HLA-B*44:02  HLA-B*44:03 | -0.4016 (No) | Yes | No |
|  | 108-116 | ISLWDQGLK | 0.85 | HLA-A*11:01 | 0.0804 (No) | No | No |
|  | 109-117 | SLWDQGLKP | 0.98 | HLA-A*02:01 | 0.0915 (No) | Yes | No |
|  | 116-124 | KPCVKLTPL | 0.09 | HLA-B*07:02  HLA-B*08:01 | 1.5232 (Yes) | No | No |
|  | 120-128 | KLTPLCVTL | 0.07 | HLA-A*02:01  HLA-A*32:01  HLA-A*02:06  HLA-A*02:03 | 2.8224 (Yes) | Yes | No |
|  | 126-134 | VTLNCNAIK | 0.53 | HLA-A*11:01 | 0.5226 (Yes) | Yes | No |
|  | 131-139 | NAIKNNTKV | 0.18 | HLA-B*51:01  HLA-A*68:02 | 0.0734 (No) | No | No |
|  | 136-144 | NTKVTNNSI | 0.67 | HLA-A*68:02 | 0.8224 (Yes) | No | No |
|  | 154-162 | CSFNITTEL | 0.56 | HLA-A*68:02 | 0.6056 (Yes) | Yes | No |
|  | 155-163 | SFNITTELR | 0.13 | HLA-A*33:01  HLA-A*31:01 | 1.4757 (Yes) | Yes | No |
|  | 158-168 | ITTELRDKK | 0.55 | HLA-A*11:01  HLA-A*68:01 | 1.7832(Yes) | No | No |
|  | 161-169 | ELRDKKRKA | 0.52 | HLA-B*08:01 | 1.1452 (Yes) | No | No |
|  | 164-172 | DKKRKAYAL | 0.02 | HLA-B*08:01 | 1.0950 (Yes) | No | No |
|  | 166-174 | KRKAYALFY | 0.25 | HLA-A*30:02 | 1.1525 (Yes) | No | No |
|  | 167-175 | RKAYALFYK | 0.88 | HLA-A*30:01 | 0.5658 (Yes) | No | No |
|  | 168-176 | KAYALFYKL | 0.05 | HLA-A*32:01  HLA-B*58:01  HLA-B*57:01  HLA-A*02:06  HLA-B*51:01  HLA-A*23:01  HLA-A*30:01  HLA-A*24:02 | 0.2534 (No) | Yes | No |
|  | 170-178 | YALFYKLDI | 0.75 | HLA-B*51:01 | 0.5033 (Yes) | No | No |
|  | 171-179 | ALFYKLDIV | 0.07 | HLA-A*02:03  HLA-A*02:01  HLA-A*02:06 | 0.8914 (Yes) | No | No |
|  | 173-181 | FYKLDIVPL | 0.27 | HLA-A*24:02  HLA-A*23:01  HLA-B*08:01 | 1.9153 (Yes) | Yes | No |
|  | 180-188 | PLNNGSTDY | 0.68 | HLA-A*01:01 | 0.7241 (Yes) | No | No |
|  | 194-202 | NTSTITQAC | 0.66 | HLA-A*68:02 | 0.2485 (No) | Yes | No |
|  | 196-204 | STITQACPK | 0.06 | HLA-A*11:01  HLA-A*03:01  HLA-A*30:01  HLA-A*68:01 | 0.5544 (Yes) | Yes | No |
|  | 199-207 | TQACPKVSL | 0.59 | HLA-B*15:01  HLA-A*02:06  HLA-B*40:01 | 0.9237 (Yes) | No | No |
|  | 202-210 | CPKVSLDPI | 0.97 | HLA-B*51:01 | 1.1671 (Yes) | Yes | No |
|  | 204-212 | KVSLDPIPI | 0.61 | HLA-A*32:01 | 1.8201 (Yes) | Yes | No |
|  | 205-213 | VSLDPIPIH | 0.8 | HLA-A*30:02 | 1.8158 (Yes) | No | No |
|  | **206-214** | **SLDPIPIHY** | **0.01** | **HLA-A*30:02**  **HLA-A*01:01**  **HLA-B*15:01**  **HLA-A*32:01**  **HLA-B*35:01**  **HLA-A*26:01**  **HLA-A*11:01**  **HLA-A*02:06**  **HLA-B*53:01**  **HLA-A*03:01**  **HLA-A*02:01**  **HLA-B*58:01**  **HLA-B*44:02**  **HLA-B*44:03**  **HLA-A*23:01**  **HLA-B*57:01** | 2.0650 (Yes) | No | No |
|  | 208-216 | DPIPIHYCA | 0.55 | HLA-B*51:01  HLA-B*35:01  HLA-B*08:01 | 0.6812 (Yes) | Yes | No |
|  | 212-220 | IHYCAPAGY | 0.77 | HLA-A*30:02 | 0.5763 (Yes) | Yes | No |
|  | 218-226 | AGYAILKCR | 0.28 | HLA-A*31:01 | 1.0018 (Yes) | Yes | No |
|  | 222-230 | ILKCRDKTF |  | HLA-B*08:01  HLA-B*15:01 | 1.0491 (Yes) | Yes | No |
|  | 234-242 | GPCHNVSTV |  | HLA-B*51:01  HLA-B*07:02 | 0.3725 (No) | No | No |
|  | 240-248 | STVQCTHGI | 0.21 | HLA-A*68:02 | 0.5294 (Yes) | Yes | No |
|  | 244-252 | CTHGIKPVV | 0.61 | HLA-A*68:02 | 0.2653 (No) | Yes | No |
|  | 249-257 | KPVVSTQLL |  | HLA-B*07:02  HLA-B*53:01  HLA-B*51:01  HLA-B*35:01 | 0.0232 (No) | No | No |
|  | 263-271 | AEGETIIRF | 0.01 | HLA-B*44:02  HLA-B*44:03  HLA-B*40:01 | 0.0540 (No) | Yes | No |
|  | 266-274 | ETIIRFENL | 0.08 | HLA-A*68:02  HLA-B*08:01  HLA-A*26:01 | 0.7659 (Yes) | No | No |
|  | 277-285 | NAKIIIVQL | 0.05 | HLA-B*08:01  HLA-B*51:01  HLA-A*68:02  HLA-B*35:01 | 0.5112 (Yes) | No | No |
|  | 281-289 | IIVQLNESV | 0.42 | HLA-A*02:06  HLA-A*02:01 | 0.2278 (No) | Yes | No |
|  | 283-291 | VQLNESVEI | 0.19 | HLA-A*02:06  HLA-A*02:01 | 0.6307 (Yes) | Yes | No |
|  | 287-295 | ESVEITCTR | 0.01 | HLA-A*68:01  HLA-A*33:01  HLA-A*26:01 | 1.8204 (Yes) | No | No |
|  | 293-301 | CTRPSNNTR | 0.12 | HLA-A*31:01  HLA-A*33:01  HLA-A*30:01  HLA-A*68:01 | 1.1030 (Yes) | Yes | No |
|  | 295-303 | RPSNNTRES | 0.81 | HLA-B*07:02 | 0.9525 (Yes) | Yes | No |
|  | 304-312 | IRIGPGQTF | 0.36 | HLA-A*23:01  HLA-A*24:02 | 0.6292 (Yes) | No | No |
|  | 305-313 | RIGPGQTFY | 0.01 | HLA-A*30:02  HLA-B*15:01  HLA-A*01:01  HLA-A*32:01  HLA-A*03:01  HLA-A*11:01  HLA-A*30:01  HLA-B*58:01  HLA-A*26:01 | 0.2907 (No) | No | No |
|  | 317-325 | DIIGDIRQA | 0.28 | HLA-A*68:02  HLA-A*26:01 | 0.3407 (No) | Yes | No |
|  | 326-334 | HCNISEEKW | 0.09 | HLA-B*58:01  HLA-B*57:01  HLA-B*53:01 | 0.3730 (No) | Yes | No |
|  | 328-336 | NISEEKWNK | 0.45 | HLA-A*68:01  HLA-A*11:01 | -0.1656 (No) | No | No |
|  | 330-338 | SEEKWNKTL | 0.05 | HLA-B*40:01  HLA-B*44:02  HLA-B*44:03 | -0.2422 (No) | No | No |
|  | 333-341 | KWNKTLQKV | 0.4 | HLA-A*24:02  HLA-A*23:01  HLA-A*30:01 | -0.3908 (No) | No | No |
|  | 336-344 | KTLQKVKEK | 0.01 | HLA-A*30:01  HLA-A*03:01  HLA-A*11:01  HLA-A*31:01 | 0.4654 (Yes) | No | No |
|  | 337-345 | TLQKVKEKL | 0.56 | HLA-B*08:01  HLA-A*02:01  HLA-A*02:03 | 0.1812 (No) | No | No |
|  | 340-348 | KVKEKLQKH | 0.2 | HLA-A*30:01  HLA-A*30:02  HLA-A*31:01  HLA-A*03:01 | -0.2224 (No) | No | No |
|  | 344-352 | KLQKHFPNK | 0.01 | HLA-A*30:01  HLA-A*03:01  HLA-A*31:01  HLA-A*11:01 | -0.7804 (No) | Yes | No |
|  | 348-356 | HFPNKTIEF | 0.03 | HLA-A*24:02  HLA-A*23:01  HLA-B*08:01  HLA-A*26:01  HLA-B*35:01 | 1.2445 (Yes) | Yes | No |
|  | 349-357 | FPNKTIEFK | 0.89 | HLA-A*68:01 | 1.3363 (Yes) | Yes | No |
|  | 352-360 | KTIEFKPSS | 0.49 | HLA-A*30:01 | 1.6613 (Yes) | Yes | No |
|  | 363-371 | DLEITTHSF | 0.39 | HLA-B*35:01  HLA-B*08:01  HLA-A*26:01  HLA-B*53:01  HLA-A*01:01 | 0.9621 (Yes) | No | No |
|  | 369-377 | HSFNCGGEF | 0.73 | HLA-B*35:01  HLA-B*15:01  HLA-A*26:01  HLA-B*58:01 | -0.0264 (No) | Yes | No |
|  | 370-378 | SFNCGGEFF | 0.33 | HLA-A*24:02  HLA-A*23:01 | -0.3600 (No) | Yes | No |
|  | 371-379 | FNCGGEFFY | 0.62 | HLA-A*01:01  HLA-A*30:02 | -0.3785 (No) | Yes | No |
|  | 378-386 | FYCNTSNLF | 0.02 | HLA-A*24:02  HLA-A*23:01 | 0.0215 (No) | Yes | No |
|  | 381-389 | NTSNLFNST | 0.71 | HLA-A*68:02 | 0.2981 (No) | No | No |
|  | 382-390 | TSNLFNSTK | 0.13 | HLA-A*11:01  HLA-A*30:01  HLA-A*68:01  HLA-A*03:01 | 0.1936 (No) | No | No |
|  | 385-393 | LFNSTKLEL | 0.64 | HLA-B*08:01  HLA-A*23:01  HLA-A*24:02 | 0.6762 (Yes) | Yes | No |
|  | 392-400 | ELFNSSTNL | 0.18 | HLA-A*68:02  HLA-A*26:01  HLA-A*02:03  HLA-A*02:01 | -0.2373 (No) | No | No |
|  | 396-404 | SSTNLNITL | 0.62 | HLA-B*58:01 | 1.5233 (Yes) | No | No |
|  | 399-407 | NLNITLQCR | 0.34 | HLA-A*33:01 | 3.0344 (Yes) | Yes | No |
|  | 403-411 | TLQCRIKQI | 0.53 | HLA-B*08:01 | 1.1087 (Yes) | Yes | No |
|  | 407-415 | RIKQIINMW | 0.01 | HLA-A*32:01  HLA-B*57:01  HLA-B*58:01  HLA-B*53:01  HLA-A*26:01  HLA-A*23:01 | -0.3742 (No) | Yes | No |
|  | 410-418 | QIINMWQGV | 0.61 | HLA-A*68:02  HLA-A*02:06 | -0.2234 (No) | Yes | No |
|  | 412-420 | INMWQGVGR | 0.6 | HLA-A*33:01  HLA-A*31:01 | -0.2094 (No) | Yes | No |
|  | 415-423 | WQGVGRAMY | 0.18 | HLA-B*15:01  HLA-A*30:02 | 0.4728 (Yes) | Yes | No |
|  | 422-430 | MYAPPIEGI | 0.04 | HLA-A*24:02  HLA-A*23:01  HLA-A*68:02 | 0.2161 (No) | No | No |
|  | 423-431 | YAPPIEGII | 0.21 | HLA-B*51:01 | 0.0914 (No) | Yes | No |
|  | 424-423 | APPIEGIIM | 0.33 | HLA-B*07:02  HLA-B*35:01 | 0.2093 (No) | No | No |
|  | 434-442 | RSNITGLLL | 0.33 | HLA-B*58:01  HLA-A*32:01  HLA-A*30:01  HLA-B*57:01 | 0.5830 (Yes) | No | No |
|  | 436-444 | NITGLLLTR | 0.09 | HLA-A*33:01  HLA-A*68:01  HLA-A*31:01 | 1.0874 (Yes) | No | No |
|  | 446-454 | GAKEPHSTK | 0.01 | HLA-A*30:01  HLA-A*03:01  HLA-A*11:01  HLA-A*31:01 | 0.2289 (No) | No | No |
|  | 448-456 | KEPHSTKEI | 0.46 | HLA-B*40:01  HLA-B*44:02  HLA-B*44:03 | 0.0441 (No) | Yes | No |
|  | 449-457 | EPHSTKEIF | 0.07 | HLA-B*53:01  HLA-B*35:01  HLA-B*07:02  HLA-B*51:01  HLA-B*08:01 | -0.1958 (No) | No | No |
|  | 452-460 | STKEIFRPE | 0.64 | HLA-A*30:01 | -0.1568 (No) | Yes | No |
|  | 460-468 | EGGDMRDNW | 0.69 | HLA-B*53:01 | 1.1456 (Yes) | No | No |
|  | 465-473 | RDNWRSELY |  | HLA-A*01:01  HLA-A*30:02  HLA-B*44:02  HLA-B*44:03 | 0.4904 (Yes) | Yes | No |
|  | 467-475 | NWRSELYKY | 0.49 | HLA-A*30:02 | -0.7657 (No) | Yes | No |
|  | 470-478 | SELYKYKVV | 0.57 | HLA-B*44:02  HLA-B*44:03  HLA-B*40:01 | -0.3269 (No) | Yes | No |
|  | 472-480 | LYKYKVVEI | 0.18 | HLA-A*24:02  HLA-A*23:01  HLA-B*08:01 | 0.5548 (Yes) | Yes | No |
|  | 478-486 | VEIKPLGVA | 0.81 | HLA-B*40:01 | 2.3572 (Yes) | No | No |
|  | 483-491 | LGVAPTKPK | 0.88 | HLA-A*30:01 | 1.8311 (Yes) | Yes | No |
|  | 484-492 | GVAPTKPKR | 0.08 | HLA-A*31:01  HLA-A*68:01  HLA-A*11:01  HLA-A*03:01  HLA-A*33:01  HLA-A*30:01 | 1.6466 (Yes) | No | No |
|  | 486-494 | APTKPKRRV | 0.08 | HLA-B*07:02  HLA-B*51:01 | 0.8728 (Yes) | No | No |
|  | 489-497 | KPKRRVVER | 0.54 | HLA-A*33:01 | -0.6592 (No) | No | No |
|  | 495-503 | VEREKRAAL | 0.14 | HLA-B*08:01  HLA-B*40:01  HLA-B*07:02  HLA-B*44:02 | 1.2707 (Yes) | No | No |
|  | 500-508 | RAALGALFL | 0.86 | HLA-B*58:01 | 0.3810 (No) | No | No |
|  | 502-510 | ALGALFLGF | 0.4 | HLA-A*32:01 | 0.8224 (Yes) | No | No |
|  | 505-513 | ALFLGFLGA |  | HLA-A*02:03  HLA-A*02:01  HLA-A*02:06 | 0.7075 (Yes) | No | No |
|  | 510-518 | FLGAAGSTM |  | HLA-B*15:01  HLA-A*02:03  HLA-A*02:01 | 0.3728 (No) | No | No |
|  | 517-525 | TMGAASITL | 0.62 | HLA-A*02:01 | 1.2135 (Yes) | No | No |
|  | 519-527 | GAASITLTV | 0.2 | HLA-A*02:06  HLA-A*68:02  HLA-A*02:03  HLA-B*51:01  HLA-A*02:01 | 1.2124 (Yes) | No | No |
|  | 522-530 | SITLTVQAR | 0.31 | HLA-A*68:01  HLA-A*33:01  HLA-A*31:01 | 2.0331 (Yes) | No | No |
|  | 525-533 | LTVQARQLL | 0.83 | HLA-B*58:01  HLA-B*57:01  HLA-A*68:02 | 1.0386 (Yes) | No | No |
|  | 537-545 | VQQQSNLLK | 0.26 | HLA-A*11:01  HLA-A*30:01  HLA-A*03:01 | -0.1659 (No) | No | No |
|  | 538-546 | QQQSNLLKA | 0.7 | HLA-A*02:06 | -0.1143 (No) | No | No |
|  | 539-547 | QQSNLLKAI | 0.94 | HLA-A*02:06 | -0.3234 (No) | No | No |
|  | 545-553 | KAIEAQQHM | 0.18 | HLA-B*58:01  HLA-B*57:01  HLA-B*35:01  HLA-B*15:01  HLA-B*53:01  HLA-A*32:01  HLA-A*26:01  HLA-A*02:06 | 0.3624 (No) | No | No |
|  | 548-556 | EAQQHMLQL | 0.12 | HLA-B*08:01  HLA-A*26:01  HLA-B*51:01  HLA-A*68:02  HLA-B*53:01  HLA-B*35:01 | 0.1486 (No) | No | No |
|  | 550-558 | QQHMLQLTV | 0.47 | HLA-A*02:06  HLA-B*15:01 | 0.4987 (Yes) | Yes | No |
|  | 551-559 | QHMLQLTVW | 0.53 | HLA-A*23:01  HLA-A*24:02  HLA-B*44:02  HLA-B*44:03  HLA-B*53:01  HLA-B*58:01 | -0.0113 (No) | Yes | No |
|  | 553-561 | MLQLTVWGI | 0.89 | HLA-A*02:01 | 0.7117 (Yes) | No | No |
|  | 556-564 | LTVWGIKQL | 0.61 | HLA-A*68:02 | 0.7061 (Yes) | No | No |
|  | 559-567 | WGIKQLQTR | 0.99 | HLA-A*33:01 | 1.0045 (Yes) | Yes | No |
|  | 560-568 | GIKQLQTRV | 0.38 | HLA-A*02:03 | 0.0356 (No) | No | No |
|  | 562-570 | KQLQTRVLA | 0.65 | HLA-A*30:01  HLA-A*02:06 | -0.4751 (No) | No | No |
|  | 563-571 | QLQTRVLAI | 0.05 | HLA-B*08:01  HLA-A*02:03  HLA-A*32:01 | 0.7482 (Yes) | Yes | No |
|  | 565-573 | QTRVLAIER | 0.13 | HLA-A*33:01  HLA-A*31:01  HLA-A*68:01  HLA-A*30:01 | 0.5712 (Yes) | No | No |
|  | 567-575 | RVLAIERHL | 0.11 | HLA-A*32:01  HLA-A*30:01  HLA-A*02:06  HLA-B*57:01  HLA-B*58:01 | 0.1467 (No) | No | No |
|  | 568-576 | VLAIERHLR | 0.34 | HLA-A*31:01  HLA-A*33:01 | 0.7502 (Yes) | Yes | No |
|  | 572-580 | ERHLRDQQL | 0.15 | HLA-B*08:01 | 0.7534 (Yes) | No | No |
|  | 576-584 | RDQQLLGIW | 0.4 | HLA-B*44:02  HLA-B*44:03 | 0.5337 (Yes) | Yes | No |
|  | 578-586 | QQLLGIWGC | 1 | HLA-A*02:06 | -0.7447 (No) | No | No |
|  | 590-598 | LICTTAVPW | 0.66 | HLA-A*32:01  HLA-B*58:01  HLA-B*57:01  HLA-B*53:01 | 0.9388 (Yes) | Yes | No |
|  | 594-602 | TAVPWNSSW | 0.01 | HLA-B*58:01  HLA-B*53:01  HLA-B*57:01  HLA-A*32:01  HLA-B*35:01  HLA-A*26:01  HLA-B*51:01 | 0.8535 (Yes) | No | No |
|  | 603-611 | SNKSQEEIW | 0.6 | HLA-B*58:01  HLA-B*57:01 | 0.2255 (No) | Yes | No |
|  | 608-616 | EEIWDNMTW | 0.01 | HLA-B*44:02  HLA-B*44:03  HLA-B*53:01 | 0.0483 (No) | Yes | No |
|  | 609-617 | EIWDNMTWM | 0.11 | HLA-A*26:01  HLA-A*68:02 | 0.1185 (No) | No | No |
|  | 611-619 | WDNMTWMQW | 0.55 | HLA-B*44:02  HLA-B*44:03 | 1.0198 (Yes) | Yes | No |
|  | 613-621 | NMTWMQWDR | 0.57 | HLA-A*33:01 | 1.3366 (Yes) | Yes | No |
|  | 615-623 | TWMQWDREI | 0.43 | HLA-A*24:02  HLA-A*23:01 | 0.4897 (Yes) | No | No |
|  | 618-626 | QWDREISNY | 0.46 | HLA-A*30:02  HLA-A*01:01 | -0.1089 (No) | No | No |
|  | 621-629 | REISNYTDI | 0.66 | HLA-B*40:01  HLA-B*44:02  HLA-B*44:03 | 0.3440 (No) | Yes | No |
|  | 622-630 | EISNYTDII | 0.74 | HLA-A*68:02 | 0.4409 (Yes) | Yes | No |
|  | 623-631 | ISNYTDIIY | 0.12 | HLA-A*01:01  HLA-A*30:02  HLA-B*35:01  HLA-B*58:01  HLA-B*15:01  HLA-B*57:01 | 0.4657 (Yes) | Yes | No |
|  | 625-633 | NYTDIIYNL | 0.01 | HLA-A*24:02  HLA-A*23:01  HLA-A*33:01  HLA-A*68:02 | 0.8322 (Yes) | Yes | No |
|  | 626-634 | YTDIIYNLL | 0.17 | HLA-A*01:01  HLA-A*02:06  HLA-A*68:02  HLA-A*02:01  HLA-B*58:01 | 0.0023 (No) | No | No |
|  | 628-636 | DIIYNLLEV | 0.12 | HLA-A*68:02  HLA-A*26:01  HLA-B*51:01  HLA-A*02:06 | 0.2513 (No) | Yes | No |
|  | 635-643 | EVSQNQQDK | 0.57 | HLA-A*68:01 | 0.2466 (No) | Yes | No |
|  | 646-654 | KDLLALDKW | 0.39 | HLA-B*44:02  HLA-B*44:03  HLA-B*58:01 | -0.4289 (No) | No | No |
|  | 649-657 | LALDKWENL | 0.52 | HLA-B*08:01  HLA-B*51:01 | 0.0227 (No) | No | No |
|  | 650-658 | ALDKWENLW | 0.16 | HLA-A*32:01  HLA-B*58:01  HLA-B*57:01  HLA-A*01:01  HLA-B*53:01 | -0.6650 (No) | No | No |
|  | 652-660 | DKWENLWNW | 0.77 | HLA-B*53:01  HLA-B*44:03  HLA-B*44:02 | -0.2817 (No) | No | No |
|  | 653-661 | KWENLWNWF | 0.32 | HLA-A*24:02  HLA-A*23:01 | -0.0138 (No) | No | No |
|  | 658-666 | WNWFNITNW | 0.89 | HLA-B*58:01  HLA-B*57:01 | 1.7292 (Yes) | No | No |
|  | 659-667 | NWFNITNWL | 0.66 | HLA-A*23:01  HLA-A*24:02 | 0.6223 (Yes) | Yes | No |
|  | 660-668 | WFNITNWLW | 0.34 | HLA-A*23:01  HLA-A*24:02  HLA-B*58:01 | 0.9517 (Yes) | Yes | No |
|  | 662-670 | NITNWLWYI | 0.62 | HLA-A*02:06  HLA-A*68:02  HLA-A*02:01 | 0.5694 (Yes) | No | No |
|  | 663-671 | ITNWLWYIK | 0.56 | HLA-A*11:01 | 0.7426 (Yes) | Yes | No |
|  | 665-673 | NWLWYIKIF | 0.26 | HLA-A*23:01  HLA-A*24:02 | 0.4972 (Yes) | Yes | No |
|  | 667-675 | LWYIKIFIM | 0.66 | HLA-A*23:01 | -0.0162 (No) | No | No |
|  | 668-676 | WYIKIFIMI | 0.36 | HLA-A*23:01  HLA-A*24:02 | 0.2196 (No) | No | No |
|  | 675-683 | MIVGGVIGL | 0.15 | HLA-A*68:02  HLA-A*02:06  HLA-A*02:01  HLA-A*26:01  HLA-A*02:03 | 0.8571 (Yes) | No | No |
|  | 676-684 | IVGGVIGLR | 0.32 | HLA-A*68:01  HLA-A*31:01  HLA-A*33:01 | 1.7387 (Yes) | Yes | No |
|  | 679-687 | GVIGLRIIF | 0.51 | HLA-A*32:01  HLA-B*15:01 | 1.7561 (Yes) | No | No |
|  | 682-690 | GLRIIFAVL | 0.82 | HLA-B*08:01 | 0.5275 (Yes) | No | No |
|  | 684-692 | RIIFAVLSI | 0.1 | HLA-A*32:01  HLA-A*30:01 | 0.8307 (Yes) | No | No |
|  | 685-693 | IIFAVLSIV | 0.43 | HLA-A*02:03  HLA-A*02:06 | 1.0045 (Yes) | No | No |
|  | 687-695 | FAVLSIVNR | 0.15 | HLA-A*68:01  HLA-A*33:01  HLA-A*31:01 | 1.4679 (Yes) | Yes | No |
|  | 688-696 | AVLSIVNRV | 0.03 | HLA-A*02:06  HLA-A*02:03  HLA-A*02:01  HLA-A*68:02  HLA-A*32:01 | 0.9020 (Yes) | Yes | No |
|  | 689-697 | VLSIVNRVR | 0.48 | HLA-A*31:01 | 1.3061 (Yes) | No | No |
|  | 692-700 | IVNRVRQGY | 0.05 | HLA-A*30:02  HLA-B*15:01  HLA-A*26:01  HLA-A*32:01  HLA-A*01:01 | 0.9549 (Yes) | Yes | No |
|  | 695-703 | RVRQGYSPL | 0.04 | HLA-A*30:01  HLA-B*07:02  HLA-A*32:01  HLA-B*08:01  HLA-B*15:01 | 1.0684 (Yes) | Yes | No |
|  | 697-705 | RQGYSPLSF | 0.03 | HLA-B*15:01  HLA-A*32:01  HLA-A*30:02  HLA-A*24:02  HLA-A*23:01 | 1.7846 (Yes) | Yes | No |
|  | 700-708 | YSPLSFQTL | 0.46 | HLA-A*24:02  HLA-B*51:01  HLA-A*23:01  HLA-B*08:01 | 1.4447 (Yes) | No | No |
|  | 701-709 | SPLSFQTLI | 0.03 | HLA-B*51:01  HLA-B*53:01  HLA-B*07:02 | 0.7824 (Yes) | No | No |
|  | 705-713 | FQTLIPHPR | 0.37 | HLA-A*33:01  HLA-A*31:01 | 0.1900 (No) | Yes | No |
|  | 707-715 | TLIPHPRGP | 0.98 | HLA-A*02:03 | 0.2112 (No) | No | No |
|  | 711-719 | HPRGPDRLG | 0.88 | HLA-B*07:02 | 0.9022 (Yes) | No | No |
|  | 726-734 | GEQGRDRSI | 0.34 | HLA-B*44:02  HLA-B*40:01  HLA-B*44:03 | 0.9970 (Yes) | No | No |
|  | 728-736 | QGRDRSIRL | 0.11 | HLA-B*08:01 | 0.3243 (No) | No | No |
|  | 732-740 | RSIRLVNGF | 0.05 | HLA-A*32:01  HLA-B*57:01  HLA-B*58:01  HLA-B*15:01  HLA-A*30:02  HLA-A*23:01 | 0.4951 (Yes) | Yes | No |
|  | 733-741 | SIRLVNGFL | 0.8 | HLA-B*07:02  HLA-A*30:01 | 0.2745 (No) | No | No |
|  | 735-743 | RLVNGFLAI | 0.12 | HLA-A*32:01  HLA-A*02:03  HLA-A*02:06  HLA-A*02:01 | 0.0666 (No) | No | No |
|  | 736-744 | LVNGFLAIF | 0.38 | HLA-A*32:01  HLA-B*15:01  HLA-A*26:01 | -0.5357 (No) | No | No |
|  | 737-745 | VNGFLAIFW | 0.72 | HLA-B*58:01  HLA-B*57:01 | -0.4158 (No) | No | No |
|  | 741-749 | LAIFWDDLR | 0.91 | HLA-A*68:01 | 0.2152 ((No) | No | No |
|  | 743-751 | IFWDDLRSL | 0.27 | HLA-A*23:01  HLA-A*24:02 | 0.1159 (No) | Yes | No |
|  | 750-758 | SLCLFSYHR | 0.21 | HLA-A*31:01  HLA-A*33:01 | 0.3280 (No) | No | No |
|  | 752-760 | CLFSYHRLR | 0.2 | HLA-A*33:01  HLA-A*31:01 | 0.3897 (No) | Yes | No |
|  | 754-762 | FSYHRLRDL | 0.33 | HLA-B*08:01 | 0.9220 (Yes) | Yes | No |
|  | 755-763 | SYHRLRDLI | 0.23 | HLA-A*24:02  HLA-A*23:01 | 0.3394 (No) | No | No |
|  | 756-764 | YHRLRDLIL | 0.25 | HLA-B*08:01 | 0.6100 (Yes) | Yes | No |
|  | 758-766 | RLRDLILIA | 0.12 | HLA-A*30:01  HLA-A*02:03  HLA-A*02:01 | 1.4626 (Yes) | No | No |
|  | 764-772 | LIAARTVEL | 0.05 | HLA-B*08:01  HLA-A*02:03  HLA-B*07:02  HLA-A*02:06  HLA-A*02:01  HLA-A*68:02 | 1.3231 (Yes) | Yes | No |
|  | 765-773 | IAARTVELL | 0.51 | HLA-B*58:01  HLA-B*51:01 | 1.1701 (Yes) | Yes | No |
|  | 770–778 | VELLGRSSL | 0.06 | HLA-B*40:01  HLA-B*44:03  HLA-B*44:02 | 0.6553 (Yes) | Yes | No |
|  | 771-779 | ELLGRSSLK | 0.67 | HLA-A*03:01  HLA-A*68:01 | 0.8204 (Yes) | No | No |
|  | 775-783 | RSSLKGLQR | 0.08 | HLA-A*31:01  HLA-A*30:01  HLA-A*11:01  HLA-A*03:01 | 0.6302 (Yes) | Yes | No |
|  | 777-785 | SLKGLQRGW | 0.11 | HLA-A*32:01  HLA-B*57:01  HLA-B*58:01  HLA-B*44:02 | 0.8411 (Yes) | No | No |
|  | 780-788 | GLQRGWETL | 0.5 | HLA-A*02:01 | 0.0504 (No) | No | No |
|  | 781-789 | LQRGWETLK | 0.43 | HLA-A*30:01  HLA-A*03:01 | 0.4486 (Yes) | No | No |
|  | 782-790 | QRGWETLKY | 0.54 | HLA-A*30:02 | 0.4936 (Yes) | No | No |
|  | 786-794 | ETLKYLGSL | 0.14 | HLA-A*68:02  HLA-A*26:01 | -0.1841 (No) | No | No |
|  | 787-795 | TLKYLGSLV | 0.46 | HLA-A*02:03 | 0.1707 (No) | No | No |
|  | 789-787 | KYLGSLVQY | 0.01 | HLA-A*30:02  HLA-A*23:01  HLA-A*24:02  HLA-A*32:01  HLA-A*30:01 | 0.0896 (No) | No | No |
|  | 790-798 | YLGSLVQYW | 0.1 | HLA-A*32:01  HLA-B*58:01  HLA-B*57:01  HLA-B*53:01  HLA-A*23:01  HLA-A*24:02 | -0.0288 (No) | No | No |
|  | 795-803 | VQYWGLELK | 0.23 | HLA-A*11:01  HLA-A*03:01  HLA-A*30:01 | 1.0991 (Yes) | No | No |
|  | 796-804 | QYWGLELKK | 0.63 | HLA-A*33:01  HLA-A*31:01  HLA-A*30:01 | 1.4224 (Yes) | No | No |
|  | 799-807 | GLELKKSAI | 0.93 | HLA-B*08:01 | 1.5496 (Yes) | No | No |
|  | 801-809 | ELKKSAINL | 0.08 | HLA-B*08:01 | 0.6586 (Yes) | Yes | No |
|  | 808-816 | NLLNTTAIV | 0.68 | HLA-A*02:01  HLA-A*02:03 | 0.6280 (Yes) | No | No |
|  | 809-817 | LLNTTAIVV | 0.42 | HLA-A*02:03  HLA-A*02:01 | 0.6129 (Yes) | No | No |
|  | 819-827 | EGTDRFIEL | 0.11 | HLA-B*08:01  HLA-B*51:01  HLA-A*68:02 | 0.1136 (No) | Yes | No |
|  | 822-830 | DRFIELIQR | 0.74 | HLA-A*33:01 | 0.2223 (No) | No | No |
|  | 823-831 | RFIELIQRI | 0.04 | HLA-A*23:01  HLA-A*24:02  HLA-A*32:01 | 0.2781 (No) | No | No |
|  | 824-832 | FIELIQRIW | 0.24 | HLA-B*53:01  HLA-B*58:01  HLA-B*57:01  HLA-A*32:01 | -0.3544 (No) | Yes | No |
|  | 826-834 | ELIQRIWRA | 0.26 | HLA-A*02:03  HLA-A*02:06  HLA-A*02:01  HLA-B*08:01  HLA-A*68:02  HLA-A*26:01 | -0.9484 (No) | Yes | No |
|  | 827-835 | LIQRIWRAF | 0.44 | HLA-A*32:01 | -0.9573 (No) | Yes | No |
|  | 830-838 | RIWRAFCNI | 0.39 | HLA-A*32:01 | -0.9025 (No) | No | No |
|  | 833-841 | RAFCNIPRR | 0.14 | HLA-A*31:01  HLA-A*30:01  HLA-A*11:01  HLA-A*03:01 | 0.5218 (Yes) | Yes | No |
|  | 834-842 | AFCNIPRRI | 0.81 | HLA-A*23:01  HLA-A*24:02 | 0.3271 (No) | Yes | No |
|  | 838-846 | IPRRIRQGL | 0.01 | HLA-B*07:02  HLA-B*08:01 | 0.5220 (Yes) | No | No |
|  | 841-849 | RIRQGLEAA | 0.19 | HLA-A*30:01  HLA-A*02:03  HLA-B*07:02 | 0.6762 (Yes) | No | No |

**Supplementary Table 11.** Identified MHC-I epitope within protease, rank, alleles, antigen, allergen, and toxic properties.

| **Sl.No** | **Position** | **Peptide** | **Rank** | **Alleles** | **Antigen** | **Allergen** | **Toxic** |
| --- | --- | --- | --- | --- | --- | --- | --- |
|  | 4-12 | TLWQRPLVT | 0.57 | HLA-A*02:01  HLA-B*08:01  HLA-A*02:03 | -0.5405 (No) | Yes | No |
|  | 5-13 | LWQRPLVTI | 0.18 | HLA-A*24:02  HLA-A*23:01 | -0.4466 (No) | Yes | No |
|  | 6-14 | WQRPLVTIK | 0.28 | HLA-A*30:01 | 0.2095 (No) | Yes | No |
|  | 11-19 | VTIKIGGQL | 0.59 | HLA-A*68:02  HLA-A*32:01  HLA-B*57:01  HLA-A*26:01  HLA-B*58:01 | 0.9793(Yes) | No | No |
|  | 12-20 | TIKIGGQLK | 0.18 | HLA-A*30:01  HLA-A*03:01  HLA-A*11:01  HLA-A*68:01 | 1.1453 (Yes) | No | No |
|  | 30-38 | DTVLEEINL | 0.52 | HLA-A*68:02 | 0.6224 (Yes) | No | No |
|  | 34-42 | EEINLPGKW | 0.01 | HLA-B*44:02  HLA-B*44:03 | 0.4087 (Yes) | No | No |
|  | 35-43 | EINLPGKWK | 0.69 | HLA-A*68:01 | 0.5227 (Yes) | Yes | No |
|  | 38-46 | LPGKWKPKM | 0.2 | HLA-B*07:02  HLA-B*53:01  HLA-B*35:01  HLA-B*51:01 | 1.4858 (Yes) | No | No |
|  | 41-49 | KWKPKMIGG | 0.89 | HLA-A*30:01 | 1.5930 (Yes) | Yes | No |
|  | 45-53 | KMIGGIGGF | 0.07 | HLA-B*15:01  HLA-A*32:01  HLA-A*30:02  HLA-A*23:01  HLA-A*26:01 | -0.0000 (No) | Yes | No |
|  | 48-56 | GGIGGFIKV | 0.49 | HLA-A*02:06 | 0.2568 (No) | Yes | No |
|  | 49-57 | GIGGFIKVR | 0.72 | HLA-A*31:01 | 1.2773 (Yes) | Yes | No |
|  | 51-59 | GGFIKVRQY | 0.22 | HLA-A*30:02  HLA-B*15:01 | 0.9374 (Yes) | Yes | No |
|  | 55-63 | KVRQYDQII | 0.64 | HLA-A*30:01  HLA-A*32:01 | 0.8504 (Yes) | No | No |
|  | 58-66 | QYDQIIIEI | 0.07 | HLA-A*24:02  HLA-A*23:01  HLA-B*08:01 | 0.4902 (Yes) | No | No |
|  | 62-70 | IIIEICGKK | 0.88 | HLA-A*03:01 | -0.4375 (No) | Yes | No |
|  | 64-72 | IEICGKKAI | 0.46 | HLA-B*40:01  HLA-B*44:02  HLA-B*44:03 | -0.1306 (No) | Yes | No |
|  | 74-82 | TVLVGPTPV | 0.38 | HLA-A*02:06  HLA-A*68:02  HLA-A*02:01 | 0.5656 (Yes) | Yes | No |
|  | 76-84 | LVGPTPVNI | 0.34 | HLA-A*02:06  HLA-A*02:03  HLA-A*68:02  HLA-A*02:01  HLA-A*32:01  HLA-B*51:01 | 1.1283 (Yes) | Yes | No |
|  | 77-85 | VGPTPVNII | 0.96 | HLA-B*51:01 | 1.3851 (Yes) | Yes | No |
|  | 87-95 | RNMLTQLGR | 0.51 | HLA-A*31:01 | -0.2854 (No) | No | No |
|  | 89-97 | MLTQLGRTL | 0.64 | HLA-A*02:03  HLA-A*32:01  HLA-B*08:01 | 0.4186 (Yes) | No | No |
|  | **91-99** | **TQLGRTLNF** | **0.04** | **HLA-B*15:01**  **HLA-A*32:01**  **HLA-A*23:01**  **HLA-A*24:02**  **HLA-A*30:02**  **HLA-A*02:06**  **HLA-B*08:01**  **HLA-A*26:01** | **1.3043 (Yes)** | **No** | **No** |

**Supplementary Table 12.** Identified MHC-I epitope within reverse transcriptase, rank, alleles, antigen, allergen, and toxic properties.

| **Sl.No** | **Position** | **Peptide** | **Rank** | **Alleles** | **Antigen** | **Allergen** | **Toxic** |
| --- | --- | --- | --- | --- | --- | --- | --- |
|  | 3-11 | SPIETVPVK | 0.84 | HLA-B*35:01 | 0.7306 (Yes) | No | No |
|  | 6-14 | ETVPVKLKP | 0.61 | HLA-A*68:02 | 1.7456 (Yes) | No | No |
|  | 8-16 | VPVKLKPGM | 0.09 | HLA-B*07:02  HLA-B*35:01  HLA-B*53:01  HLA-B*51:01 | 1.7005 (Yes) | No | No |
|  | 13-21 | KPGMDGPKV | 0.64 | HLA-B*07:02 | 0.3484 (No) | No | No |
|  | 16-24 | MDGPKVKQW | 0.12 | HLA-B*44:02  HLA-B*44:03  HLA-B*58:01  HLA-B*57:01 | -0.2803 (No) | Yes | No |
|  | 18-26 | GPKVKQWPL | 0.03 | HLA-B*08:01  HLA-B*07:02 | 1.2470 (Yes) | Yes | No |
|  | 20-28 | KVKQWPLTE | 0.2 | HLA-A*30:01 | 0.8254 (Yes) | No | No |
|  | 22-30 | KQWPLTEEK | 0.16 | HLA-A*11:01  HLA-A*30:01  HLA-A*03:01  HLA-A*31:01  HLA-A*32:01 | 0.9772 (Yes) | Yes | No |
|  | 23-31 | QWPLTEEKI | 0.43 | HLA-A*24:02  HLA-A*23:01 | 0.7600 (Yes) | No | No |
|  | 26-34 | LTEEKIKAL | 0.25 | HLA-B*08:01 | 0.6429 ((Yes) | Yes | No |
|  | 28-36 | EEKIKALTA | 0.87 | HLA-B*08:01 | 0.2757 (No) | Yes | No |
|  | 39-47 | EEMEQEGKI | 0.22 | HLA-B*44:03  HLA-B*44:02  HLA-B*40:01 | 0.5129 (Yes) | No | No |
|  | 41-49 | MEQEGKISR | 0.49 | HLA-A*33:01 | 0.4297 (Yes) | No | No |
|  | 48-56 | SRIGPENPY | 0.57 | HLA-A*30:02  HLA-A*26:01 | 0.9059 (Yes) | No | No |
|  | 54-62 | NPYNTPVFA | 0.41 | HLA-B*51:01  HLA-B*35:01 | 0.1426 (No) | Yes | No |
|  | 55-63 | PYNTPVFAI | 0.16 | HLA-A*24:02  HLA-A*23:01 | -0.0120 (No) | Yes | No |
|  | 56-64 | YNTPVFAIK | 0.92 | HLA-A*68:01 | 0.5853 (Yes) | Yes | No |
|  | 57-65 | NTPVFAIKK | 0.21 | HLA-A*68:01  HLA-A*11:01 | 0.3657 (No) | No | No |
|  | 61-69 | FAIKKKDST | 0.92 | HLA-B*08:01 | 1.7076 (Yes) | No | No |
|  | 62-70 | AIKKKDSTK | 0.09 | HLA-A*30:01  HLA-A*03:01 | 0.3514 (No) | No | No |
|  | 70-78 | KWRKLVDFR | 0.25 | HLA-A*31:01  HLA-A*30:01 | 0.4644 (Yes) | No | No |
|  | 74-52 | LVDFRELNK | 0.39 | HLA-A*11:01  HLA-A*03:01 | 1.2932 (Yes) | No | No |
|  | 79-87 | ELNKRTQDF | 0.08 | HLA-B*08:01  HLA-A*26:01 | 1.2483 (Yes) | Yes | No |
|  | 80-88 | LNKRTQDFW | 0.4 | HLA-B*58:01  HLA-B*57:01 | 1.1757 (Yes) | No | No |
|  | 84-92 | TQDFWEVQL | 0.22 | HLA-A*02:06  HLA-A*02:01  HLA-B*40:01 | 0.7160 (Yes) | No | No |
|  | 90-98 | VQLGIPHPA | 0.28 | HLA-A*02:06 | 0.5082 (Yes) | No | No |
|  | 92-100 | LGIPHPAGL | 0.95 | HLA-B*08:01 | 0.4445 (Yes) | No | No |
|  | 93-101 | GIPHPAGLK | 0.31 | HLA-A*03:01  HLA-A*11:01 | 0.2177 (No) | No | No |
|  | 98-106 | AGLKKKKSV | 0.3 | HLA-B*08:01 | 1.6990 (Yes) | No | No |
|  | 100-108 | LKKKKSVTV | 0.12 | HLA-B*08:01 | 1.0493 (Yes) | Yes | No |
|  | 101-109 | KKKKSVTVL | 0.9 | HLA-B*08:01 | 0.9772 (Yes) | No | No |
|  | 107-115 | TVLDVGDAY | 0.06 | HLA-A*26:01  HLA-B*35:01  HLA-A*30:02  HLA-B*15:01  HLA-A*01:01  HLA-B*53:01 | 1.0832 (Yes) | Yes | No |
|  | 108-116 | VLDVGDAYF | 0.46 | HLA-A*01:01  HLA-A*02:06  HLA-A*02:01 | 1.1965 (Yes) | Yes | No |
|  | 110-118 | DVGDAYFSV | 0.05 | HLA-A*68:02  HLA-B*51:01  HLA-A*26:01 | 1.2097 (Yes) | No | No |
|  | 116-124 | FSVPLDEGF | 0.27 | HLA-B*58:01  HLA-B*53:01  HLA-B*57:01  HLA-B*35:01  HLA-A*26:01 | 0.1063 (No) | Yes | No |
|  | 117-125 | SVPLDEGFR | 0.81 | HLA-A*68:01 | 0.4714 (Yes) | No | No |
|  | 119-127 | PLDEGFRKY | 0.08 | HLA-A*01:01  HLA-A*30:02 | 0.1161 (No) | No | No |
|  | 122-130 | EGFRKYTAF | 0.06 | HLA-B*08:01  HLA-A*26:01  HLA-B*51:01 | -0.2776 (No) | Yes | No |
|  | 127-135 | YTAFTIPST | 0.32 | HLA-A*68:02 | 0.7424 (Yes) | No | No |
|  | 134-142 | STNNETPGI | 0.4 | HLA-A*68:02 | 0.6735 (Yes) | Yes | No |
|  | 136-144 | NNETPGIRY | 0.25 | HLA-A*01:01 | 0.4586 (Yes) | No | No |
|  | 138-146 | ETPGIRYQY | 0.01 | HLA-A*26:01  HLA-A*01:01  HLA-A*68:01  HLA-B*35:01  HLA-A*30:02  HLA-B*53:01  HLA-A*68:02  HLA-A*32:01  HLA-A*33:01 | 0.7690 (Yes) | No | No |
|  | 141-149 | GIRYQYNVL | 0.91 | HLA-B*07:02  HLA-B*08:01 | 1.1128 (Yes) | Yes | No |
|  | 145-153 | QYNVLPQGW | 0.07 | HLA-A*24:02  HLA-A*23:01  HLA-B*58:01  HLA-A*32:01  HLA-B*53:01  HLA-B*57:01 | 0.5708 (Yes) | Yes | No |
|  | 152-160 | GWKGSPPIF | 0.19 | HLA-A*23:01  HLA-A*24:02 | 0.5481 (Yes) | No | No |
|  | 156-164 | SPPIFQSSM | 0.09 | HLA-B*07:02  HLA-B*35:01  HLA-B*51:01  HLA-B*08:01  HLA-B*53:01 | 0.1769 (No) | No | No |
|  | 159-167 | IFQSSMPQI | 0.24 | HLA-A*24:02  HLA-A*23:01 | -0.5487 (No) | Yes | No |
|  | 160-168 | FQSSMPQIL | 0.26 | HLA-A*02:06  HLA-B*15:01  HLA-B*40:01  HLA-A*02:01  HLA-A*02:03 | -0.3852 (No) | Yes | No |
|  | 163-171 | SMPQILEPF | 0.2 | HLA-A*24:02  HLA-A*23:01  HLA-B*15:01  HLA-A*32:01  HLA-A*26:01 | -0.3719 (No) | No | No |
|  | 164-172 | MPQILEPFR | 0.83 | HLA-A*33:01 | -0.2298 (No) | Yes | No |
|  | 173-181 | APNPEIVIY | 0.01 | HLA-B*35:01  HLA-B*53:01  HLA-A*26:01  HLA-A*30:02  HLA-A*01:01  HLA-B*07:02  HLA-B*44:02  HLA-B*44:03  HLA-B*15:01  HLA-B*51:01 | 0.4587 (Yes) | Yes | No |
|  | 175-183 | NPEIVIYQY | 0.02 | HLA-B*35:01  HLA-B*53:01  HLA-A*01:01 | 0.6135 (Yes) | No | No |
|  | 176-184 | PEIVIYQYM | 0.99 | HLA-B*44:03 | 0.1683 (No) | No | No |
|  | 180-188 | IYQYMDDLY | 0.54 | HLA-A*24:02  HLA-A*23:01  HLA-A*30:02 | -0.3419 (No) | No | No |
|  | 181-189 | YQYMDDLYV | 0.08 | HLA-A*02:06  HLA-A*02:01  HLA-A*02:03 | 0.3369 (No) | No | No |
|  | 187-195 | LYVGSDLEI | 0.33 | HLA-A*24:02  HLA-A*23:01 | 0.7082 (Yes) | Yes | No |
|  | 200-208 | APIEELREH | 0.35 | HLA-B*35:01 | 0.2921 (No) | Yes | No |
|  | 202-210 | IEELREHLL | 0.15 | HLA-B*40:01  HLA-B*44:02  HLA-B*44:03 | -0.1598 (No) | Yes | No |
|  | 203-211 | EELREHLLK | 0.99 | HLA-B*44:03 | -1.3889 (No) | Yes | No |
|  | 204-212 | ELREHLLKW | 0.15 | HLA-A*26:01  HLA-A*32:01  HLA-B*53:01  HLA-B*57:01  HLA-B*58:01  HLA-B*44:02  HLA-B*08:01 | -1.1405 (No) | No | No |
|  | 206-214 | REHLLKWGF | 0.07 | HLA-B*44:03  HLA-B*44:02  HLA-B*40:01 | -0.2427 (No) | No | No |
|  | 208-216 | HLLKWGFTT | 0.91 | HLA-A*02:01 | 0.4947 (Yes) | No | No |
|  | 215-223 | TTPDKKHQK | 0.34 | HLA-A*11:01  HLA-A*68:01  HLA-A*30:01  HLA-A*03:01 | 1.3026 (Yes) | Yes | No |
|  | 221-229 | HQKEPPFLW | 0.02 | HLA-A*32:01  HLA-B*58:01  HLA-B*57:01  HLA-B*53:01  HLA-B*44:02  HLA-A*23:01  HLA-B*44:03  HLA-B*15:01  HLA-A*24:02  HLA-A*30:02 | 1.5310 (Yes) | Yes | No |
|  | 224-232 | EPPFLWMGY | 0.41 | HLA-B*35:01  HLA-A*26:01  HLA-B*53:01 | 1.1903 (Yes) | No | No |
|  | 226-234 | PFLWMGYEL | 0.43 | HLA-A*23:01  HLA-A*24:02 | 1.3418 (Yes) | Yes | No |
|  | 231-239 | GYELHPDKW | 0.37 | HLA-A*24:02  HLA-A*23:01 | -0.3063 (No) | Yes | No |
|  | 233-241 | ELHPDKWTV | 0.13 | HLA-A*68:02  HLA-A*02:01  HLA-A*02:03  HLA-A*02:06  HLA-B*08:01 | -0.2210 (No) | Yes | No |
|  | 235-243 | HPDKWTVQP | 0.97 | HLA-B*35:01  HLA-B*53:01 | 0.3054 (No) | No | No |
|  | 238-246 | KWTVQPIQL | 0.2 | HLA-A*24:02  HLA-A*23:01 | 1.4921 (Yes) | Yes | No |
|  | 241-249 | VQPIQLPEK | 0.89 | HLA-A*11:01  HLA-A*30:01 | 1.0388 (Yes) | No | No |
|  | 244-252 | IQLPEKDSW | 0.12 | HLA-B*58:01  HLA-B*57:01  HLA-A*32:01  HLA-B*53:01  HLA-A*23:01  HLA-B*44:02  HLA-B*44:03  HLA-A*24:02  HLA-B*15:01 | 0.7913 (Yes) | Yes | No |
|  | 246-254 | LPEKDSWTV | 0.18 | HLA-B*53:01  HLA-B*51:01  HLA-B*35:01 | 0.5140 (Yes) | Yes | No |
|  | 252-260 | WTVNDIQKL | 0.49 | HLA-A*68:02  HLA-A*26:01  HLA-A*02:06 | 0.4445 (Yes) | Yes | No |
|  | 253-261 | TVNDIQKLV | 0.02 | HLA-A*68:02  HLA-A*02:03  HLA-A*02:06  HLA-A*26:01 | -0.1819 (No) | Yes | No |
|  | 256-264 | DIQKLVGKL | 0.88 | HLA-A*26:01 | -0.8129 (No) | Yes | No |
|  | 259-267 | KLVGKLNWA | 0.1 | HLA-A*02:03  HLA-A*02:01  HLA-A*02:06 | 1.2411 (Yes) | Yes | No |
|  | 263-271 | KLNWASQIY | 0.03 | HLA-A*30:02  HLA-B*15:01  HLA-A*32:01  HLA-A*03:01  HLA-A*01:01 | 1.0520 (Yes) | Yes | No |
|  | 266-274 | WASQIYPGI | 0.61 | HLA-B*51:01  HLA-A*68:02 | 0.4444 (Yes) | Yes | No |
|  | 267-275 | ASQIYPGIK | 0.53 | HLA-A*11:01  HLA-A*30:01 | 0.8424 (Yes) | Yes | No |
|  | 268-276 | SQIYPGIKV | 0.09 | HLA-A*02:06  HLA-A*02:03  HLA-A*02:01  HLA-B*15:01  HLA-B*40:01 | 0.9748 (Yes) | Yes | No |
|  | 269-277 | QIYPGIKVR | 0.04 | HLA-A*31:01  HLA-A*68:01  HLA-A*33:01  HLA-A*03:01  HLA-A*11:01  HLA-A*30:01 | 2.1516 (Yes) | Yes | No |
|  | 271-279 | YPGIKVRQL | 0.01 | HLA-B*08:01  HLA-B*07:02  HLA-B*51:01  HLA-B*53:01  HLA-B*35:01 | 1.8876 (Yes) | Yes | No |
|  | 273-281 | GIKVRQLCK | 0.55 | HLA-A*30:01  HLA-A*03:01 | 2.0182 (Yes) | Yes | No |
|  | 275-283 | KVRQLCKLL | 0.35 | HLA-A*30:01 | 0.9168 (Yes) | Yes | No |
|  | 281-289 | KLLRGAKAL | 0.38 | HLA-A*32:01  HLA-A*02:01  HLA-A*02:03 | -0.2599 (No) | No | No |
|  | 287-295 | KALTDIVTL | 0.21 | HLA-A*02:06  HLA-B*58:01  HLA-A*32:01  HLA-B*57:01  HLA-A*02:01 | -0.1445 (No) | No | No |
|  | 288-296 | ALTDIVTLT | 0.28 | HLA-A*02:03  HLA-A*02:01  HLA-A*02:06 | 0.5414 (Yes) | Yes | No |
|  | 289-297 | LTDIVTLTE | 0.73 | HLA-A*01:01 | 0.5902 (Yes) | No | No |
|  | 291-299 | DIVTLTEEA | 0.9 | HLA-A*68:02 | 0.7238 (Yes) | Yes | No |
|  | 295-303 | LTEEAELEL | 0.54 | HLA-A*01:01 | 0.8639 (Yes) | No | No |
|  | 299-307 | AELELAENR | 0.9 | HLA-B*44:03 | 0.7332 (Yes) | Yes | No |
|  | 301-309 | LELAENREI | 0.32 | HLA-B*40:01  HLA-B*44:02  HLA-B*44:03 | 0.7399 (Yes) | Yes | No |
|  | 302-310 | ELAENREIL | 0.54 | HLA-A*68:02  HLA-B*08:01 | 0.0691 (No) | Yes | No |
|  | 304-312 | AENREILKE | 0.95 | HLA-B*44:03 | -0.5307 (No) | Yes | No |
|  | 307-315 | REILKEPVH | 0.95 | HLA-B*44:03  HLA-B*40:01 | -1.3657 (No) | No | No |
|  | 309-317 | ILKEPVHGV | 0.01 | HLA-A*02:03  HLA-A*02:01  HLA-A*02:06  HLA-B*08:01  HLA-A*68:02  HLA-A*32:01 | -0.6194 (No) | Yes | No |
|  | 311-319 | KEPVHGVFY | 0.2 | HLA-B*44:02  HLA-B*44:03  HLA-A*30:02 | 0.0863 (No) | Yes | No |
|  | 317-325 | VFYDPSKDL | 0.52 | HLA-A*23:01  HLA-A*24:02 | -0.4150 (No) | No | No |
|  | 318-326 | FYDPSKDLI | 0.17 | HLA-A*24:02  HLA-A*23:01  HLA-B*08:01 | -0.5751 (No) | No | No |
|  | 329-337 | IQKQGNDQW | 0.3 | HLA-B*58:01  HLA-B*15:01  HLA-B*57:01  HLA-A*32:01  HLA-B*44:02  HLA-B*44:03  HLA-B*53:01 | 0.3063 (No) | Yes | No |
|  | 331-339 | KQGNDQWTF | 0.15 | HLA-A*32:01  HLA-B*15:01  HLA-A*23:01  HLA-A*24:02  HLA-A*30:02  HLA-B*58:01 | 0.7187 (Yes) | Yes | No |
|  | 334-342 | NDQWTFQFY | 0.71 | HLA-A*01:01  HLA-B*44:03  HLA-B*44:02 | 0.7620 (Yes) | Yes | No |
|  | 338-346 | TFQFYQEPF | 0.32 | HLA-A*24:02  HLA-A*23:01 | 0.2823 (No) | Yes | No |
|  | 339-347 | FQFYQEPFK | 0.78 | HLA-A*11:01 | -0.0272 (No) | No | No |
|  | 341-49 | FYQEPFKNL | 0.03 | HLA-A*24:02  HLA-A*23:01  HLA-B*08:01 | -0.6019 (No) | No | No |
|  | 342-350 | YQEPFKNLK | 0.9 | HLA-A*11:01 | -0.0389 (No) | No | No |
|  | 348-356 | NLKTGKFAK | 0.73 | HLA-A*30:01  HLA-A*03:01 | 0.8258 (Yes) | Yes | No |
|  | 353-361 | KFAKRGTAH | 0.93 | HLA-A*30:02 | 0.9709 (Yes) | No | No |
|  | 358-366 | GTAHTNDVK | 0.34 | HLA-A*11:01 | 0.8242 (Yes) | Yes | No |
|  | 361-369 | HTNDVKQLT | 0.57 | HLA-A*68:02 | 0.2102 (No) | Yes | No |
|  | 364-372 | DVKQLTAVV | 0.29 | HLA-A*68:02  HLA-B*08:01  HLA-A*26:01  HLA-B*51:01 | 0.5280 (Yes) | No | No |
|  | 366-374 | KQLTAVVQK | 0.04 | HLA-A*03:01  HLA-A*30:01  HLA-A*11:01  HLA-A*31:01 | 0.5123 (Yes) | No | No |
|  | 367-375 | QLTAVVQKI | 0.26 | HLA-A*02:03  HLA-A*02:01  HLA-A*02:06  HLA-A*32:01 | 0.4610 (Yes) | Yes | No |
|  | 369-377 | TAVVQKIAL | 0.3 | HLA-B*08:01  HLA-B*35:01  HLA-B*51:01  HLA-B*07:02 | 0.2449 (No) | Yes | No |
|  | 372-380 | VQKIALESI | 0.73 | HLA-B*15:01 | 0.2432 (No) | Yes | No |
|  | 374-382 | KIALESIVI | 0.61 | HLA-A*32:01 | 0.4691 (Yes) | No | No |
|  | 375-383 | IALESIVIW | 0.01 | HLA-B*58:01  HLA-B*57:01  HLA-B*53:01  HLA-A*32:01  HLA-B*51:01  HLA-B*35:01  HLA-A*23:01 | 0.2585 (No) | No | No |
|  | 380-388 | IVIWGKTPK | 0.16 | HLA-A*11:01  HLA-A*03:01  HLA-A*30:01  HLA-A*68:01  HLA-A*31:01 | 0.3428 (No) | No | No |
|  | **381-389** | **VIWGKTPKF** | **0.02** | **HLA-A*32:01**  **HLA-A*23:01**  **HLA-A*24:02**  **HLA-B*15:01**  **HLA-A*26:01**  **HLA-A*30:02**  **HLA-B*58:01**  **HLA-B*57:01**  **HLA-B*08:01**  **HLA-B*53:01**  **HLA-A*02:06** | **0.4408 (Yes)** | **No** | **No** |
|  | 382-390 | IWGKTPKFR | 0.9 | HLA-A*31:01 | -0.4566 (No) | No | No |
|  | 383-391 | WGKTPKFRL | 0.9 | HLA-B*08:01 | -0.3437 (No) | Yes | No |
|  | 385-393 | KTPKFRLPI | 0.43 | HLA-A*32:01  HLA-A*30:01 | -1.0728 (No) | No | No |
|  | 388-396 | KFRLPIQKE | 0.47 | HLA-A*30:01 | -0.6356 (No) | Yes | No |
|  | 390-398 | RLPIQKETW | 0.07 | HLA-A*32:01  HLA-B*58:01  HLA-A*24:02  HLA-A*23:01  HLA-B*57:01 | -0.6583 (No) | Yes | No |
|  | 393-401 | IQKETWEAW | 0.16 | HLA-A*32:01  HLA-B*15:01  HLA-B*58:01  HLA-B*57:01  HLA-A*23:01  HLA-B*44:02  HLA-B*44:03  HLA-B*53:01  HLA-A*24:02 | 0.0969 (No) | No | No |
|  | 398-406 | WEAWWTDYW | 0.29 | HLA-B*44:02  HLA-B*44:03 | -0.5167 (No) | Yes | No |
|  | 402-410 | WTDYWQATW | 0.11 | HLA-B*58:01  HLA-B*57:01  HLA-B*53:01  HLA-A*32:01  HLA-A*01:01 | -0.0832 (No) | Yes | No |
|  | 406-414 | WQATWIPEW | 0.21 | HLA-A*32:01  HLA-B*58:01  HLA-B*53:01  HLA-B*57:01  HLA-A*23:01  HLA-B*44:03  HLA-B*44:02  HLA-A*24:02 | 0.3115 (No) | Yes | No |
|  | 408-416 | ATWIPEWEF | 0.05 | HLA-A*32:01  HLA-B*58:01  HLA-B*57:01  HLA-A*23:01  HLA-A*24:02  HLA-B*53:01 | 0.2164 (No) | No | No |
|  | 409-417 | TWIPEWEFV | 0.4 | HLA-A*24:02  HLA-A*23:01 | 0.2744 (No) | Yes | No |
|  | 414-422 | WEFVNTPPL | 0.11 | HLA-B*40:01 | 1.0825 (Yes) | Yes | No |
|  | 416-424 | FVNTPPLVK | 0.03 | HLA-A*03:01  HLA-A*11:01  HLA-A*30:01  HLA-A*68:01 | -0.0133 (No) | Yes | No |
|  | 418-426 | NTPPLVKLW | 0.07 | HLA-B*58:01  HLA-B*57:01  HLA-B*53:01  HLA-A*32:01  HLA-A*26:01  HLA-A*23:01  HLA-A*24:02  HLA-B*44:02 | -0.5587 (No) | Yes | No |
|  | 419-427 | TPPLVKLWY | 0.24 | HLA-B*35:01  HLA-B*53:01 | -0.3699 (No) | Yes | No |
|  | 427-435 | YQLEKEPIA | 0.18 | HLA-A*02:06  HLA-A*02:01 | 0.3955 (No) | No | No |
|  | 429-437 | LEKEPIAGV | 0.45 | HLA-B*40:01  HLA-B*44:02  HLA-B*44:03 | 0.8218 (Yes) | No | No |
|  | 432-440 | EPIAGVETF | 0.01 | HLA-B*53:01  HLA-B*35:01  HLA-B*51:01  HLA-A*26:01  HLA-B*07:02  HLA-B*08:01 | 0.0922 (No) | No | No |
|  | 433-441 | PIAGVETFY | 0.52 | HLA-A*30:02  HLA-A*01:01  HLA-A*26:01 | 0.3091 (No) | No | No |
|  | 438-446 | ETFYVDGAA | 0.28 | HLA-A*68:02 | -0.4588 (No) | Yes | No |
|  | 440-448 | FYVDGAANR | 0.09 | HLA-A*33:01  HLA-A*31:01 | -0.2736 (No) | No | No |
|  | 446-454 | ANRETKIGK | 0.26 | HLA-A*30:01 | 1.2863 (Yes) | No | No |
|  | 449-457 | ETKIGKAGY | 0.04 | HLA-A*26:01  HLA-A*01:01  HLA-A*30:02 | 1.5151 (Yes) | Yes | No |
|  | 457-465 | YVTDRGRQK | 0.7 | HLA-A*68:01  HLA-A*03:01  HLA-A*11:01  HLA-A*30:01 | 1.0962 (Yes) | No | No |
|  | 461-469 | RGRQKIVSL | 0.03 | HLA-B*08:01  HLA-A*30:01  HLA-B*07:02  HLA-A*32:01 | 1.0787 (Yes) | Yes | No |
|  | 463-471 | RQKIVSLTE | 0.97 | HLA-A*30:01 | 1.0858 (Yes) | No | No |
|  | 468-476 | SLTETTNQK | 0.16 | HLA-A*03:01  HLA-A*11:01  HLA-A*30:01 | 1.0581 (Yes) | No | No |
|  | 471-479 | ETTNQKTEL | 0.21 | HLA-A*68:02  HLA-A*26:01 | 0.8225 (Yes) | No | No |
|  | 474-482 | NQKTELQAI | 0.52 | HLA-B*08:01  HLA-B*15:01 | 0.8993 (Yes) | Yes | No |
|  | 476-484 | KTELQAIQL | 0.78 | HLA-A*32:01 | 1.0459 (Yes) | No | No |
|  | 477-485 | TELQAIQLA | 0.55 | HLA-B*44:03  HLA-B*44:02  HLA-B*40:01 | 1.1490 (Yes) | No | No |
|  | 478-486 | ELQAIQLAL | 0.34 | HLA-B*08:01  HLA-A*68:02 | 1.1607 (Yes) | No | No |
|  | 485-493 | ALQDSGSEV | 0.04 | HLA-A*02:03  HLA-A*02:01  HLA-A*02:06 | -0.0894 (No) | No | No |
|  | 488-496 | DSGSEVNIV | 0.89 | HLA-B*51:01 | 0.5007 (Yes) | Yes | No |
|  | 493-501 | VNIVTDSQY | 0.57 | HLA-A*30:02 | 0.5435 (Yes) | Yes | No |
|  | 495-503 | IVTDSQYAL | 0.34 | HLA-A*02:06  HLA-A*68:02  HLA-A*02:01  HLA-A*32:01  HLA-B*35:01 | 0.6930 (Yes) | Yes | No |
|  | 499-507 | SQYALGIIL | 0.2 | HLA-B*15:01  HLA-A*02:06  HLA-A*32:01  HLA-B*40:01 | 0.6180 (Yes) | No | No |
|  | 504-512 | GIILAQPDK | 0.84 | HLA-A*11:01 | 0.4037 (Yes) | Yes | No |
|  | 509-517 | QPDKSESEI | 0.29 | HLA-B*53:01  HLA-B*07:02  HLA-B*51:01  HLA-B*35:01 | 0.1636 (No) | No | No |
|  | 513-521 | SESEIVNQI | 0.01 | HLA-B*44:02  HLA-B*44:03  HLA-B*40:01 | 0.0816 (No) | Yes | No |
|  | 517-525 | IVNQIIEQL | 0.11 | HLA-A*02:06  HLA-A*32:01  HLA-A*68:02  HLA-A*02:03  HLA-A*02:01  HLA-B*58:01  HLA-B*53:01 | -0.6867 (No) | No | No |
|  | 520-528 | QIIEQLISK | 0.02 | HLA-A*11:01  HLA-A*03:01  HLA-A*68:01  HLA-A*30:01 | -0.7865 (No) | Yes | No |
|  | 524-532 | QLISKERVY | 0.04 | HLA-B*15:01  HLA-A*30:02  HLA-A*26:01 | 0.5160 (Yes) | Yes | No |
|  | 530-538 | RVYLSWVPA | 0.16 | HLA-A*30:01 | 1.0546 (Yes) | Yes | No |
|  | 531-539 | VYLSWVPAH | 0.65 | HLA-A*30:02 | 1.5887 (Yes) | Yes | No |
|  | 532-540 | YLSWVPAHK | 0.13 | HLA-A*03:01  HLA-A*11:01  HLA-A*68:01  HLA-A*30:01 | 1.4085 (Yes) | Yes | No |
|  | 534-542 | SWVPAHKGI | 0.59 | HLA-A*24:02  HLA-A*23:01 | 1.0662 (Yes) | No | No |
|  | 550-558 | KLVSSGIRK | 0.01 | HLA-A*03:01  HLA-A*11:01  HLA-A*30:01 | 0.4233 (Yes) | No | No |
|  | 551-559 | LVSSGIRKV | 0.22 | HLA-A*02:03  HLA-A*02:06  HLA-A*68:02 | 0.3300 (No) | Yes | No |
|  | 552-560 | VSSGIRKVL | 0.85 | HLA-B*07:02  HLA-B*58:01  HLA-B*57:01 | -0.1897 (No) | Yes | No |

**Supplementary Table 13.** Identified MHC-I epitope within integrase, rank, alleles, antigen, allergen, and toxic properties.

| **Sl.No** | **Position** | **Peptide** | **Rank** | **Alleles** | **Antigen** | **Allergen** | **Toxic** |
| --- | --- | --- | --- | --- | --- | --- | --- |
|  | 7-15 | KAQEEHEKY | 0.1 | HLA-A*30:02  HLA-B*58:01  HLA-A*01:01  HLA-B*35:01  HLA-B*15:01  HLA-B*57:01  HLA-B*53:01 | 0.4242 (Yes) | No | No |
|  | 14-22 | KYHSNWRAM | 0.23 | HLA-A*24:02  HLA-A*23:01  HLA-A*30:01 | 0.4299 (Yes) | No | No |
|  | 18-26 | NWRAMANEF | 0.39 | HLA-A*24:02  HLA-A*23:01 | 0.0976 (No) | No | No |
|  | 20-28 | RAMANEFNI | 0.68 | HLA-A*32:01  HLA-B*58:01 | 0.0218 (No) | No | No |
|  | 24-32 | NEFNIPPVV | 0.11 | HLA-B*40:01  HLA-B*44:03  HLA-B*44:02 | 1.2608 (Yes) | Yes | No |
|  | 26-34 | FNIPPVVPK | 0.29 | HLA-A*68:01  HLA-A*11:01  HLA-A*30:01  HLA-A*33:01  HLA-A*03:01 | 1.1610 (Yes) | Yes | No |
|  | 28-36 | IPPVVPKEI | 0.01 | HLA-B*51:01  HLA-B*53:01  HLA-B*07:02 | 0.8306 (Yes) | Yes | No |
|  | 32-40 | VPKEIVACC | 0.66 | HLA-B*51:01 | -0.0034 (No) | Yes | Yes |
|  | 48-56 | EAIHGQVNC | 0.84 | HLA-A*68:02 | 0.4816 (Yes) | Yes | No |
|  | 52-60 | GQVNCSPGI | 0.81 | HLA-A*02:06 | 1.1564 (Yes) | Yes | No |
|  | 53-61 | QVNCSPGIW | 0.63 | HLA-B*58:01  HLA-B*57:01  HLA-B*53:01 | 1.0547 (Yes) | Yes | No |
|  | 55-63 | NCSPGIWQL | 0.81 | HLA-A*68:02 | 0.5364 (Yes) | Yes | No |
|  | 60-68 | IWQLDCTHL | 0.65 | HLA-A*24:02  HLA-A*23:01 | 1.2211 (Yes) | No | No |
|  | 67-75 | HLEGKIILV | 0.09 | HLA-A*02:03  HLA-A*02:01  HLA-A*02:06  HLA-B*08:01 | 0.4599 (Yes) | Yes | No |
|  | 69-77 | EGKIILVAV | 0.43 | HLA-B*08:01  HLA-B*51:01  HLA-A*68:02 | 0.5643 (Yes) | No | No |
|  | 71-79 | KIILVAVHV | 0.28 | HLA-A*02:06  HLA-A*02:01  HLA-A*32:01  HLA-A*02:03 | 0.5522 (Yes) | No | No |
|  | **75-83** | **VAVHVASGY** | **0.16** | **HLA-A*30:02**  **HLA-B*35:01**  **HLA-A*26:01**  **HLA-B*15:01**  **HLA-A*01:01**  **HLA-B*53:01**  **HLA-B*58:01** | **0.5921 (Yes)** | **No** | **No** |
|  | 78-86 | HVASGYIEA | 0.15 | HLA-A*68:02  HLA-A*02:06  HLA-A*02:03 | 0.6180 (Yes) | No | No |
|  | 81-89 | SGYIEAEVI | 0.75 | HLA-B*51:01 | 0.3498 (No) | No | No |
|  | 87-95 | EVIPAETGQ | 0.89 | HLA-A*26:01 | 1.0110 (Yes) | Yes | No |
|  | 89-97 | IPAETGQET | 0.48 | HLA-B*35:01  HLA-B*07:02 | 0.7765 (Yes) | Yes | No |
|  | 91-99 | AETGQETAY | 0.03 | HLA-B*44:02  HLA-B*44:03  HLA-B*40:01 | 0.8177 (Yes) | Yes | No |
|  | 92-100 | ETGQETAYF | 0.08 | HLA-A*26:01 | 0.6537 (Yes) | Yes | No |
|  | 96-104 | ETAYFLLKL | 0.01 | HLA-A*68:02  HLA-A*26:01 | 0.5625 (Yes) | No | No |
|  | 99-107 | YFLLKLAGR | 0.1 | HLA-A*33:01  HLA-A*31:01 | 0.5979 (Yes) | Yes | No |
|  | 100-108 | FLLKLAGRW | 0.38 | HLA-A*32:01  HLA-B*58:01  HLA-B*57:01  HLA-B*53:01 | 0.3960 (No) | Yes | No |
|  | 103-111 | KLAGRWPVR | 0.05 | HLA-A*31:01  HLA-A*03:01  HLA-A*30:01  HLA-A*33:01 | 0.5860 (Yes) | No | No |
|  | 113-121 | IHTDNGSNF | 0.82 | HLA-A*23:01  HLA-A*24:02 | 0.0361 (No) | No | No |
|  | 114-122 | HTDNGSNFT | 0.86 | HLA-A*01:01 | 0.4588 (Yes) | Yes | No |
|  | 119-127 | SNFTSNAVK | 0.91 | HLA-A*30:01 | 0.2265 (No) | No | No |
|  | 121-129 | FTSNAVKAA | 0.66 | HLA-A*68:02 | 0.3159 (No) | Yes | No |
|  | 123-131 | SNAVKAACW | 0.89 | HLA-B*58:01 | 1.0384 (Yes) | No | No |
|  | 124-132 | NAVKAACWW | 0.29 | HLA-B*53:01  HLA-B*58:01  HLA-B*57:01 | 0.9507 (Yes) | No | No |
|  | 131-139 | WWAGIQQEF | 0.12 | HLA-A*23:01  HLA-A*24:02 | 0.0905 (No) | Yes | No |
|  | 135-143 | IQQEFGIPY | 0.02 | HLA-B*15:01  HLA-A*30:02  HLA-A*32:01  HLA-B*35:01 | 0.5273 (Yes) | No | No |
|  | 137-145 | QEFGIPYNP | 0.62 | HLA-B*44:03  HLA-B*40:01  HLA-B*44:02 | 0.5653 (Yes) | No | No |
|  | 142-150 | PYNPQSQGV | 0.55 | HLA-A*24:02  HLA-A*23:01 | 1.0162 (Yes) | Yes | No |
|  | 146-154 | QSQGVVESM | 0.64 | HLA-B*35:01  HLA-A*26:01  HLA-A*68:02  HLA-B*58:01 | 0.2832 (No) | Yes | No |
|  | 152-160 | ESMNKELKK | 0.28 | HLA-A*68:01  HLA-A*11:01 | -0.3368 (No) | No | No |
|  | 153-161 | SMNKELKKI | 0.23 | HLA-A*02:03  HLA-A*32:01 | -0.2286 (No) | No | No |
|  | 157-165 | ELKKIIGQV | 0.4 | HLA-B*08:01  HLA-A*02:03  HLA-A*68:02  HLA-A*26:01 | -0.9096 (No) | Yes | No |
|  | 163-171 | GQVREQAEH | 0.88 | HLA-B*15:01 | 0.7093 (Yes) | No | No |
|  | 164-172 | QVREQAEHL | 0.59 | HLA-B*08:01  HLA-A*68:02  HLA-B*07:02 | 0.7500 (Yes) | Yes | No |
|  | 167-175 | EQAEHLKTA | 0.72 | HLA-A*02:03  HLA-A*02:06 | 0.3578 (No) | Yes | No |
|  | 171-179 | HLKTAVQMA | 0.34 | HLA-A*02:03 | 0.6954 (Yes) | No | No |
|  | 173-181 | KTAVQMAVF | 0.11 | HLA-A*32:01  HLA-B*57:01  HLA-B*58:01  HLA-B*15:01  HLA-A*30:02 | 0.1923 (No) | Yes | No |
|  | 174-182 | TAVQMAVFI | 0.31 | HLA-B*51:01 | 0.3539 (No) | No | No |
|  | 177-185 | QMAVFIHNF | 0.07 | HLA-A*32:01  HLA-A*23:01  HLA-A*24:02  HLA-B*15:01  HLA-A*26:01  HLA-B*58:01 | -0.3472 (No) | No | No |
|  | 178-186 | MAVFIHNFK | 0.12 | HLA-A*68:01  HLA-A*11:01  HLA-A*30:01  HLA-A*33:01 | -0.0861 (No) | Yes | No |
|  | 179-187 | AVFIHNFKR | 0.01 | HLA-A*31:01  HLA-A*11:01  HLA-A*68:01  HLA-A*33:01  HLA-A*03:01  HLA-A*30:01 | -0.2629 (No) | No | No |
|  | 180-188 | VFIHNFKRR | 0.26 | HLA-A*33:01  HLA-A*31:01 | -0.0015 (No) | Yes | No |
|  | 186-194 | KRRGGIGGY | 0.71 | HLA-A*30:02 | 1.9931 (Yes) | No | No |
|  | 203-211 | IIASDIQTK | 0.15 | HLA-A*11:01  HLA-A*03:01  HLA-A*68:01 | 0.7446 (Yes) | Yes | No |
|  | 205-213 | ASDIQTKEL | 0.74 | HLA-A*01:01 | 1.0735 (Yes) | No | No |
|  | 209-217 | QTKELQNQI | 0.17 | HLA-A*68:02  HLA-A*32:01  HLA-A*30:01  HLA-B*57:01 | 0.2207 (No) | Yes | No |
|  | 215-223 | NQILKIQNF | 0.1 | HLA-B*15:01  HLA-B*08:01  HLA-A*23:01  HLA-A*24:02  HLA-A*32:01  HLA-A*26:01  HLA-B*44:02  HLA-B*44:03 | 0.3071 (No) | Yes | No |
|  | 216-224 | QILKIQNFR | 0.09 | HLA-A*31:01  HLA-A*33:01  HLA-A*68:01  HLA-A*11:01 | 0.0761 (No) | Yes | No |
|  | 217-225 | ILKIQNFRV | 0.42 | HLA-A*02:03  HLA-A*02:01 | 0.2117 (No) | Yes | No |
|  | 218-226 | LKIQNFRVY | 0.73 | HLA-B*15:01  HLA-A*30:02 | 0.2200 (No) | Yes | No |
|  | 219-227 | KIQNFRVYY | 0.02 | HLA-A*30:02  HLA-A*32:01  HLA-A*30:01  HLA-B*15:01  HLA-A*03:01  HLA-A*11:01  HLA-A*31:01  HLA-A*01:01 | -0.1548 (No) | No | No |
|  | 220-228 | IQNFRVYYR | 0.03 | HLA-A*31:01  HLA-A*33:01 | 0.4244 (Yes) | No | No |
|  | 226-234 | YYRDSRDPI | 0.7 | HLA-A*24:02  HLA-A*23:01 | 0.2840 (No) | Yes | No |
|  | 234-242 | IWKGPAKLL | 0.35 | HLA-A*23:01  HLA-A*24:02 | -1.0497 (No) | Yes | No |
|  | 235-243 | WKGPAKLLW | 0.86 | HLA-B*58:01 | -0.3898 (No) | Yes | No |
|  | 236-244 | KGPAKLLWK | 0.85 | HLA-A*30:01 | -0.0247 (No) | Yes | No |
|  | 240-248 | KLLWKGEGA | 1 | HLA-A*02:03 | 0.6217 (Yes) | Yes | No |
|  | 241-249 | LLWKGEGAV | 0.44 | HLA-A*02:01  HLA-A*02:03 | 0.3312 (No) | Yes | No |
|  | 250-258 | VIQDNSDIK | 0.8 | HLA-A*11:01 | 0.3190 (No) | Yes | No |
|  | 251-259 | IQDNSDIKV | 0.33 | HLA-A*02:06  HLA-A*02:01 | 0.5041 (Yes) | Yes | No |
|  | 254-262 | NSDIKVVPR | 0.29 | HLA-A*33:01  HLA-A*68:01 | 1.2728 (Yes) | Yes | No |
|  | 256-264 | DIKVVPRRK | 0.54 | HLA-A*33:01  HLA-A*68:01 | 1.9621 (Yes) | No | No |
|  | 258-266 | KVVPRRKAK | 0.02 | HLA-A*30:01  HLA-A*03:01  HLA-A*31:01  HLA-A*11:01 | 1.1585 (Yes) | No | No |
|  | 260-268 | VPRRKAKII | 0.11 | HLA-B*08:01  HLA-B*07:02  HLA-B*51:01 | 1.1692 (Yes) | Yes | No |
|  | 263-271 | RKAKIIRDY | 0.44 | HLA-A*30:02 | -0.8462 (No) | Yes | No |
|  | 267-275 | IIRDYGKQM | 0.74 | HLA-B*07:02  HLA-B*15:01  HLA-B*08:01 | -0.9624 (No) | Yes | No |
|  | 273-281 | KQMAGADCV | 0.59 | HLA-A*02:06 | 0.2632 (No) | No | No |

Supplementary Table 14. Identified MHC-II epitope within envelop glycoprotein, rank, alleles, antigen, allergen, and toxic properties.

| **Sl.No** | **Position** | **Peptide** | **Rank** | **Alleles** | **Antigen** | **Allergen** | **Toxic** |
| --- | --- | --- | --- | --- | --- | --- | --- |
|  | 30-44 | MGNLWVTVYYGVPVW | 2.9 | HLA-DRB1*15:01 | -0.1824 (No) | No | No |
|  | 35-49 | VTVYYGVPVWKDASP | 1.5 | HLA-DRB1*07:01  HLA-DRB3*01:01  HLA-DRB3*02:02  HLA-DRB5*01:01 | 0.3790  (No) | Yes | No |
|  | 40-54 | GVPVWKDASPTLFCA | 2.3 | HLA-DRB1*03:01  HLA-DRB3*01:01 | 0.5731  (Yes) | No | No |
|  | 49-63 | PTLFCASDAKAYDTE | 4.1 | HLA-DRB1*07:01  HLA-DRB3*01:01  HLA-DRB3*02:02  HLA-DRB5*01:01 | 0.7943  (Yes) | Yes | No |
|  | 56-70 | DAKAYDTEVHNVWGT | 13 | HLA-DRB3*01:01 | 0.4356  (Yes) | No | No |
|  | 68-82 | WGTFACVPTDPSPQE | 1.4 | HLA-DRB1*07:01  HLA-DRB3*01:01  HLA-DRB3*02:02  HLA-DRB4*01:01  HLA-DRB5*01:01 | 0.6515  (Yes) | No | No |
|  | 92-106 | FNMWKNDMVEQMHQD | 0.64 | HLA-DRB1*03:01  HLA-DRB3*01:01  HLA-DRB3*02:02 | -0.4677  (No) | No | No |
|  | 100-114 | VEQMHQDIISLWDQG | 5.3 | HLA-DRB1*03:01  HLA-DRB3*01:01 | -0.0042  (No) | No | No |
|  | 105-119 | QDIISLWDQGLKPCV | 8.6 | HLA-DRB1*15:01 | -0.1820  (No) | Yes | No |
|  | 131-145 | NAIKNNTKVTNNSIN | 6 | HLA-DRB1*03:01  HLA-DRB3*02:02 | 0.4261  (Yes) | No | No |
|  | 136-150 | NTKVTNNSINSANDE | 0.62 | HLA-DRB1*03:01  HLA-DRB3*01:01  HLA-DRB3*02:02  HLA-DRB4*01:01 | 0.6844  (Yes) | No | No |
|  | 141-155 | NNSINSANDEMKNCS | 18 | HLA-DRB5*01:01 | 0.1808  (No) | Yes | No |
|  | 155-169 | SFNITTELRDKKRKA | 3.2 | HLA-DRB1*03:01  HLA-DRB1*07:01  HLA-DRB4*01:01 | 1.2910  (Yes) | No | No |
|  | 171-185 | ALFYKLDIVPLNNGS | 0.58 | HLA-DRB1*03:01  HLA-DRB3*01:01  HLA-DRB3*02:02  HLA-DRB4*01:01  HLA-DRB5*01:01 | 1.2264  (Yes) | Yes | No |
|  | 187-201 | DYRLINCNTSTITQA | 2.1 | HLA-DRB3*02:02 | 0.1991  (No) | No | No |
|  | 203-217 | PKVSLDPIPIHYCAP | 8 | HLA-DRB1*03:01  HLA-DRB3*01:01 | 0.7152  (Yes) | Yes | No |
|  | 208-222 | DPIPIHYCAPAGYAI | 13 | HLA-DRB1*03:01  HLA-DRB3*01:01  HLA-DRB5*01:01 | 0.8677  (Yes) | Yes | No |
|  | 214-228 | YCAPAGYAILKCRDK | 8.3 | HLA-DRB5*01:01 | 1.2248  (Yes) | No | No |
|  | 227-241 | DKTFTGTGPCHNVST | 15 | HLA-DRB1*07:01  HLA-DRB3*02:02 | 0.4105  (Yes) | Yes | No |
|  | 245-259 | THGIKPVVSTQLLLN | 0.48 | HLA-DRB1*07:01  HLA-DRB1*15:01  HLA-DRB3*01:01  HLA-DRB3*02:02  HLA-DRB5*01:01 | 0.0619  (No) | No | No |
|  | 253-267 | STQLLLNGSIAEGET | 1.3 | HLA-DRB1*03:01  HLA-DRB3*01:01  HLA-DRB3*02:02 | 0.0812  (No) | Yes | No |
|  | 259-273 | NGSIAEGETIIRFEN | 5.7 | HLA-DRB1*03:01  HLA-DRB5*01:01 | 0.4968  (Yes) | Yes | No |
|  | 265-279 | GETIIRFENLTNNAK | 0.25 | HLA-DRB1*03:01  HLA-DRB1*07:01  HLA-DRB1*15:01  HLA-DRB3*02:02  HLA-DRB4*01:01  HLA-DRB5*01:01 | 0.6413 (Yes) | Yes | No |
|  | 270-284 | RFENLTNNAKIIIVQ | 6.2 | HLA-DRB1*03:01  HLA-DRB3*02:02  HLA-DRB5*01:01 | 0.5499  (Yes). | Yes | No |
|  | 278-292 | AKIIIVQLNESVEIT | 2.6 | HLA-DRB1*03:01  HLA-DRB1*07:01  HLA-DRB1*15:01  HLA-DRB3*01:01  HLA-DRB3*02:02  HLA-DRB4*01:01 | 0.6585  (Yes) | No | No |
|  | 286-300 | NESVEITCTRPSNNT | 17 | HLA-DRB1*07:01 | 1.1805  (Yes) | Yes | No |
|  | 301-315 | RESIRIGPGQTFYAT | 4.3 | HLA-DRB1*03:01  HLA-DRB1*07:01  HLA-DRB1*15:01  HLA-DRB3*01:01  HLA-DRB3*02:02  HLA-DRB4*01:01  HLA-DRB5*01:01 | 0.6858  (Yes) | Yes | No |
|  | 309-323 | GQTFYATGDIIGDIR | 3.4 | HLA-DRB1*07:01  HLA-DRB3*01:01  HLA-DRB3*02:02  HLA-DRB4*01:01  HLA-DRB5*01:01 | 0.1076  (No) | No | No |
|  | 315-329 | TGDIIGDIRQAHCNI | 0.12 | HLA-DRB1*03:01  HLA-DRB3*01:01 | 0.3327  (No) | Yes | No |
|  | 320-334 | GDIRQAHCNISEEKW | 5.8 | HLA-DRB1*07:01  HLA-DRB3*02:02  HLA-DRB4*01:01 | 0.4511  (Yes) | Yes | No |
|  | 329-343 | ISEEKWNKTLQKVKE | 13 | HLA-DRB3*02:02  HLA-DRB5*01:01 | 0.0314  (No) | No | No |
|  | 334-348 | WNKTLQKVKEKLQKH | 12 | HLA-DRB1*15:01  HLA-DRB5*01:01 | -0.3261  (No) | No | No |
|  | 345-359 | LQKHFPNKTIEFKPS | 15 | HLA-DRB3*01:01  HLA-DRB3*02:02 | 0.6355  (Yes) | No | No |
|  | **351-365** | **NKTIEFKPSSGGDLE** | **0.96** | **HLA-DRB1*07:01**  **HLA-DRB1*15:01**  **HLA-DRB3*01:01**  **HLA-DRB3*02:02**  **HLA-DRB4*01:01**  **HLA-DRB5*01:01** | **1.3159  (Yes)** | **No** | **No** |
|  | 375-389 | GEFFYCNTSNLFNST | 0.51 | HLA-DRB1*07:01  HLA-DRB3*01:01  HLA-DRB3*02:02  HLA-DRB5*01:01 | -0.1198  (No) | Yes | No |
|  | 382-396 | TSNLFNSTKLELFNS | 7 | HLA-DRB1*03:01  HLA-DRB1*07:01  HLA-DRB3*01:01 | 0.1726  (No) | No | No |
|  | 390-404 | KLELFNSSTNLNITL | 2.4 | HLA-DRB1*07:01  HLA-DRB3*01:01  HLA-DRB3*02:02 | 0.7482  (Yes) | No | No |
|  | 409-423 | KQIINMWQGVGRAMY | 2.6 | HLA-DRB1*15:01  HLA-DRB5*01:01 | -0.1544  (No) | Yes | No |
|  | 426-440 | PIEGIIMCRSNITGL | 13 | HLA-DRB1*15:01  HLA-DRB3*02:02 | 0.4760  (Yes) | Yes | No |
|  | 434-448 | RSNITGLLLTRDGAK | 8.6 | HLA-DRB4*01:01 | 0.4535  (Yes) | No | No |
|  | 439-453 | GLLLTRDGAKEPHST | 16 | HLA-DRB1*03:01  HLA-DRB3*01:01 | 0.3419  (No) | No | No |
|  | 453-467 | TKEIFRPEGGDMRDN | 1.4 | HLA-DRB1*03:01  HLA-DRB3*01:01  HLA-DRB3*02:02  HLA-DRB4*01:01  HLA-DRB5*01:01 | 0.1409  (No) | No | No |
|  | 461-475 | GGDMRDNWRSELYKY | 11 | HLA-DRB1*03:01 | 0.1046 (No) | Yes | No |
|  | 468-482 | WRSELYKYKVVEIKP | 0.29 | HLA-DRB1*07:01  HLA-DRB1*15:01  HLA-DRB3*02:02  HLA-DRB5*01:01 | 0.2075  (No) | Yes | No |
|  | 474-488 | KYKVVEIKPLGVAPT | 1.8 | HLA-DRB1*03:01  HLA-DRB1*07:01  HLA-DRB1*15:01  HLA-DRB3*01:01  HLA-DRB3*02:02  HLA-DRB4*01:01  HLA-DRB5*01:01 | 1.4294  (Yes) | Yes | No |
|  | 482-496 | PLGVAPTKPKRRVVE | 1.9 | HLA-DRB1*03:01  HLA-DRB3*02:02  HLA-DRB5*01:01 | 1.1003  (Yes) | No | No |
|  | 491-505 | KRRVVEREKRAALGA | 2.8 | HLA-DRB1*03:01  HLA-DRB4*01:01 | 0.7603  (Yes) | No | No |
|  | 503-517 | LGALFLGFLGAAGST | 16 | HLA-DRB1*15:01  HLA-DRB5*01:01 | 0.6966  (Yes) | No | No |
|  | 521-535 | ASITLTVQARQLLSG | 7.3 | HLA-DRB1*03:01  HLA-DRB4*01:01 | 1.0835  (Yes) | No | No |
|  | 532-546 | LLSGIVQQQSNLLKA | 0.62 | HLA-DRB1*03:01  HLA-DRB1*07:01  HLA-DRB1*15:01  HLA-DRB3*01:01  HLA-DRB3*02:02  HLA-DRB4*01:01  HLA-DRB5*01:01 | 0.1464  (No) | No | No |
|  | 543-557 | LLKAIEAQQHMLQLT | 4.3 | HLA-DRB1*03:01  HLA-DRB4*01:01 | 0.3442  (No) | Yes | No |
|  | 553-567 | MLQLTVWGIKQLQTR | 12 | HLA-DRB1*15:01 | 0.7729  (Yes) | No | No |
|  | 558-572 | VWGIKQLQTRVLAIE | 2.3 | HLA-DRB1*07:01  HLA-DRB1*15:01  HLA-DRB5*01:01 | 0.5995  (Yes) | No | No |
|  | 565-579 | QTRVLAIERHLRDQQ | 1.6 | HLA-DRB1*03:01  HLA-DRB1*15:01  HLA-DRB3*01:01  HLA-DRB3*02:02  HLA-DRB4*01:01  HLA-DRB5*01:01 | 0.5709  (Yes) | Yes | No |
|  | 571-585 | IERHLRDQQLLGIWG | 3.7 | HLA-DRB4*01:01 | 0.3112  (No) | Yes | No |
|  | 587-601 | SGKLICTTAVPWNSS | 9.4 | HLA-DRB1*07:01  HLA-DRB3*02:02 | 0.5305  (Yes) | Yes | No |
|  | 595-609 | AVPWNSSWSNKSQEE | 2.6 | HLA-DRB1*07:01  HLA-DRB3*01:01  HLA-DRB3*02:02  HLA-DRB5*01:01 | 0.8972  (Yes) | No | No |
|  | 607-621 | QEEIWDNMTWMQWDR | 7.3 | HLA-DRB3*02:02 | 0.4209  (Yes) | Yes | No |
|  | 614-628 | MTWMQWDREISNYTD | 14 | HLA-DRB1*03:01  HLA-DRB3*01:01 | 0.5477  (Yes) | Yes | No |
|  | 619-633 | WDREISNYTDIIYNL | 1.3 | HLA-DRB1*15:01  HLA-DRB3*01:01 | 0.2429  (No) | No | No |
|  | 626-640 | YTDIIYNLLEVSQNQ | 3 | HLA-DRB1*03:01  HLA-DRB3*02:02  HLA-DRB4*01:01 | 0.3475  (No) | Yes | No |
|  | 631-645 | YNLLEVSQNQQDKNE | 7.2 | HLA-DRB1*03:01  HLA-DRB1*07:01  HLA-DRB3*01:01  HLA-DRB3*02:02  HLA-DRB4*01:01 | 0.5765  (Yes) | Yes | No |
|  | 645-659 | EKDLLALDKWENLWN | 19 | HLA-DRB1*03:01  HLA-DRB3*01:01 | -0.1795  (No) | No | No |
|  | 682-696 | GLRIIFAVLSIVNRV | 12 | HLA-DRB5*01:01 | 1.1076  (Yes) | No | No |
|  | 687-701 | FAVLSIVNRVRQGYS | 8.1 | HLA-DRB1*03:01  HLA-DRB5*01:01 | 1.1076  (Yes) | No | No |
|  | 702-716 | PLSFQTLIPHPRGPD | 0.01 | HLA-DRB1*03:01  HLA-DRB1*07:01  HLA-DRB1*15:01  HLA-DRB3*01:01  HLA-DRB3*02:02  HLA-DRB4*01:01  HLA-DRB5*01:01 | 0.6613  (Yes) | Yes | No |
|  | 718-732 | LGGIEEEGGEQGRDR | 12 | HLA-DRB4*01:01 | 0.8137  (Yes) | Yes | No |
|  | 725-739 | GGEQGRDRSIRLVNG | 9.2 | HLA-DRB3*01:01 | 0.5974  (Yes) | No | No |
|  | 730-744 | RDRSIRLVNGFLAIF | 0.81 | HLA-DRB1*07:01  HLA-DRB1*15:01  HLA-DRB5*01:01 | 0.1814  (No) | No | No |
|  | 737-751 | VNGFLAIFWDDLRSL | 14 | HLA-DRB1*15:01 | -0.1658  (No) | Yes | No |
|  | 755-769 | SYHRLRDLILIAART | 11 | HLA-DRB4*01:01 | 0.9806  (Yes) | No | No |
|  | 760-774 | RDLILIAARTVELLG | 5.6 | HLA-DRB1*03:01  HLA-DRB1*07:01  HLA-DRB1*15:01 | 0.9707  (Yes) | Yes | No |
|  | 766-780 | AARTVELLGRSSLKG | 17 | HLA-DRB1*03:01 | 1.0095  (Yes) | No | No |
|  | 785-799 | WETLKYLGSLVQYWG | 18 | HLA-DRB1*15:01 | -0.2928  (No) | No | No |
|  | 791-805 | LGSLVQYWGLELKKS | 0.53 | HLA-DRB1*15:01 | 0.7713  (Yes) | No | No |
|  | 799-813 | GLELKKSAINLLNTT | 5.8 | HLA-DRB1*07:01  HLA-DRB3*02:02  HLA-DRB4*01:01 | 1.2367  (Yes) | No | No |
|  | 804-818 | KSAINLLNTTAIVVG | 1.9 | HLA-DRB1*07:01  HLA-DRB1*15:01  HLA-DRB3*01:01  HLA-DRB3*02:02  HLA-DRB4*01:01  HLA-DRB5*01:01 | 0.8452  (Yes) | Yes | No |
|  | 812-826 | TTAIVVGEGTDRFIE | 3.5 | HLA-DRB1*03:01  HLA-DRB3*01:01  HLA-DRB4*01:01  HLA-DRB5*01:01 | 0.3210  (No) | No | No |
|  | 821-835 | TDRFIELIQRIWRAF | 8.6 | HLA-DRB1*03:01 | -0.4820  (No) | Yes | No |
|  | 829-843 | QRIWRAFCNIPRRIR | 1.8 | HLA-DRB1*15:01  HLA-DRB3*02:02  HLA-DRB5*01:01 | -0.2828  (No) | Yes | No |

Supplementary Table 15. Identified MHC-II epitope within protease, rank, alleles, antigen, allergen, and toxic properties.

| **Sl.No** | **Position** | **Peptide** | **Rank** | **Alleles** | **Antigen** | **Allergen** | **Toxic** |
| --- | --- | --- | --- | --- | --- | --- | --- |
|  | 1-15 | PQITLWQRPLVTIKI | 11 | HLA-DRB1*15:01 | 0.3406 (No) | Yes | No |
|  | 7-21 | QRPLVTIKIGGQLKE | 3.2 | HLA-DRB1*15:01  HLA-DRB4*01:01  HLA-DRB5*01:01 | 0.7925 (Yes) | Yes | No |
|  | 12-26 | TIKIGGQLKEALLDT | 15 | HLA-DRB4*01:01 | 0.6381 (Yes) | Yes | No |
|  | 18-32 | QLKEALLDTGADDTV | 4.8 | HLA-DRB4*01:01 | 0.2053 (No) | No | No |
|  | 29-43 | DDTVLEEINLPGKWK | 4.4 | HLA-DRB1*03:01  HLA-DRB1*15:01  HLA-DRB3*02:02  HLA-DRB4*01:01  HLA-DRB5*01:01 | 0.3280 (No) | Yes | No |
|  | **42-56** | **WKPKMIGGIGGFIKV** | **13** | **HLA-DRB5*01:01** | **0.6796 (Yes)** | **No** | **No** |
|  | 47-61 | IGGIGGFIKVRQYDQ | 11 | HLA-DRB1*15:01 | 0.9722 (Yes) | Yes | No |
|  | 52-66 | GFIKVRQYDQIIIEI | 2.8 | HLA-DRB1*15:01 | 0.9734 (Yes) | Yes | No |
|  | 61-75 | QIIIEICGKKAIGTV | 8.9 | HLA-DRB1*03:01 | -0.2708 (No) | Yes | No |
|  | 68-82 | GKKAIGTVLVGPTPV | 20 | HLA-DRB4*01:01 | 0.2512 (No) | Yes | No |
|  | 73-87 | GTVLVGPTPVNIIGR | 3.4 | HLA-DRB1*07:01  HLA-DRB3*02:02 | 0.4446 (Yes) | Yes | No |
|  | 78-92 | GPTPVNIIGRNMLTQ | 6.7 | HLA-DRB1*03:01  HLA-DRB1*15:01 | -0.0042 (No) | Yes | No |

Supplementary Table 16. Identified MHC-II epitope within reverse transcriptase, rank, alleles, antigen, allergen, and toxic properties.

| **Sl.No** | **Position** | **Peptide** | **Rank** | **Alleles** | **Antigen** | **Allergen** | **Toxic** |
| --- | --- | --- | --- | --- | --- | --- | --- |
|  | 1-15 | PISPIETVPVKLKPG | 0.8 | HLA-DRB1*03:01  HLA-DRB1*07:01  HLA-DRB1*15:01  HLA-DRB4*01:01  HLA-DRB5*01:01 | 1.3995  (Yes) | Yes | No |
|  | 7-21 | TVPVKLKPGMDGPKV | 17 | HLA-DRB1*15:01  HLA-DRB5*01:01 | 0.8673  (Yes) | No | No |
|  | 17-31 | DGPKVKQWPLTEEKI | 15 | HLA-DRB1*15:01  HLA-DRB4*01:01 | 0.4707  (Yes) | Yes | No |
|  | 28-42 | EEKIKALTAICEEME | 14 | HLA-DRB1*15:01  HLA-DRB4*01:01  HLA-DRB5*01:01 | 0.4701  (Yes) | Yes | No |
|  | 39-53 | EEMEQEGKISRIGPE | 2.9 | HLA-DRB1*03:01 | 0.8604  (Yes) | No | No |
|  | 56-70 | YNTPVFAIKKKDSTK | 14 | HLA-DRB1*03:01  HLA-DRB4*01:01  HLA-DRB5*01:01 | 0.6146  (Yes) | No | No |
|  | 67-81 | DSTKWRKLVDFRELN | 16 | HLA-DRB5*01:01 | 0.5522  (Yes) | No | No |
|  | 72-86 | RKLVDFRELNKRTQD | 13 | HLA-DRB1*15:01  HLA-DRB4*01:01  HLA-DRB5*01:01 | 0.6841  (Yes) | No | No |
|  | 83-97 | RTQDFWEVQLGIPHP | 19 | HLA-DRB5*01:01 | 0.9740  (Yes) | No | No |
|  | 97-111 | PAGLKKKKSVTVLDV | 4.1 | HLA-DRB1*07:01  HLA-DRB1*15:01  HLA-DRB5*01:01 | 1.3684  (Yes) | No | No |
|  | 104-118 | KSVTVLDVGDAYFSV | 5.3 | HLA-DRB1*03:01  HLA-DRB3*01:01  HLA-DRB4*01:01 | 1.1753  (Yes) | Yes | No |
|  | 110-124 | DVGDAYFSVPLDEGF | 3.2 | HLA-DRB4*01:01 | 0.6006  (Yes) | No | No |
|  | 121-135 | DEGFRKYTAFTIPST | 0.3 | HLA-DRB1*07:01  HLA-DRB1*15:01  HLA-DRB3*01:01  HLA-DRB3*02:02  HLA-DRB5*01:01 | 0.1705  (No) | No | No |
|  | 127-141 | YTAFTIPSTNNETPG | 16 | HLA-DRB3*02:02 | 0.6239  (Yes) | No | No |
|  | 138-152 | ETPGIRYQYNVLPQG | 0.99 | HLA-DRB1*15:01  HLA-DRB3*02:02  HLA-DRB4*01:01  HLA-DRB5*01:01 | 0.6347  (Yes) | No | No |
|  | 143-157 | RYQYNVLPQGWKGSP | 1.8 | HLA-DRB3*02:02  HLA-DRB5*01:01 | 0.5988  (Yes) | No | No |
|  | 149-163 | LPQGWKGSPPIFQSS | 5.5 | HLA-DRB1*07:01  HLA-DRB3*02:02  HLA-DRB5*01:01 | 0.1398  (No) | No | No |
|  | 156-170 | SPPIFQSSMPQILEP | 0.32 | HLA-DRB1*07:01  HLA-DRB3*01:01  HLA-DRB3*02:02  HLA-DRB5*01:01 | -0.0557  (No) | Yes | No |
|  | 163-177 | SMPQILEPFRAPNPE | 6.4 | HLA-DRB1*15:01 | -0.0851  (No) | No | No |
|  | 168-182 | LEPFRAPNPEIVIYQ | 7.3 | HLA-DRB1*07:01  HLA-DRB3*01:01  HLA-DRB3*02:02 | 0.1673  (No) | No | No |
|  | 177-191 | EIVIYQYMDDLYVGS | 4.5 | HLA-DRB1*07:01  HLA-DRB1*15:01  HLA-DRB3*01:01  HLA-DRB4*01:01  HLA-DRB5*01:01 | 0.4304  (Yes) | No | No |
|  | 186-200 | DLYVGSDLEIGQHRA | 14 | HLA-DRB1*03:01  HLA-DRB4*01:01 | 0.8290  (Yes) | Yes | No |
|  | 197-211 | QHRAPIEELREHLLK | 4.5 | HLA-DRB1*15:01 | -0.4720  (No) | Yes | No |
|  | 212-226 | WGFTTPDKKHQKEPP | 6.5 | HLA-DRB1*03:01 | 1.3190  (Yes) | Yes | No |
|  | 224-238 | EPPFLWMGYELHPDK | 19 | HLA-DRB5*01:01 | 0.7855  (Yes) | Yes | No |
|  | 231-245 | GYELHPDKWTVQPIQ | 11 | HLA-DRB1*03:01  HLA-DRB3*01:01 | 0.4598  (Yes) | No | No |
|  | 236-250 | PDKWTVQPIQLPEKD | 1.7 | HLA-DRB3*01:01  HLA-DRB3*02:02  HLA-DRB4*01:01  HLA-DRB5*01:01 | 0.7475  (Yes) | Yes | No |
|  | 249-263 | KDSWTVNDIQKLVGK | 0.1 | HLA-DRB1*03:01  HLA-DRB1*07:01  HLA-DRB3*01:01  HLA-DRB3*02:02  HLA-DRB4*01:01  HLA-DRB5*01:01 | 0.0413  (No) | No | No |
|  | 257-271 | IQKLVGKLNWASQIY | 12 | HLA-DRB1*15:01 | 0.2302  (No) | Yes | No |
|  | 267-281 | ASQIYPGIKVRQLCK | 16 | HLA-DRB1*03:01 | 1.3145  (Yes) | Yes | No |
|  | 273-287 | GIKVRQLCKLLRGAK | 8.9 | HLA-DRB1*03:01 | 0.7897  (Yes) | No | No |
|  | 279-293 | LCKLLRGAKALTDIV | 17 | HLA-DRB1*07:01 | -0.3243  (No) | No | No |
|  | 285-299 | GAKALTDIVTLTEEA | 16 | HLA-DRB3*01:01 | 0.3781  (No) | Yes | No |
|  | 290-304 | TDIVTLTEEAELELA | 7.8 | HLA-DRB1*07:01 | 0.8583  (Yes) | No | No |
|  | 299-313 | AELELAENREILKEP | 6.2 | HLA-DRB1*03:01  HLA-DRB1*07:01  HLA-DRB1*15:01  HLA-DRB3*02:02  HLA-DRB5*01:01 | -0.0327  (No) | Yes | No |
|  | 306-320 | NREILKEPVHGVFYD | 3 | HLA-DRB1*03:01  HLA-DRB1*07:01  HLA-DRB1*15:01  HLA-DRB3*01:01  HLA-DRB3*02:02  HLA-DRB4*01:01  HLA-DRB5*01:01 | -0.5868  (No) | Yes | No |
|  | 314-328 | VHGVFYDPSKDLIAE | 0.54 | HLA-DRB1*03:01  HLA-DRB1*07:01  HLA-DRB1*15:01  HLA-DRB3*01:01  HLA-DRB3*02:02 | -0.4336  (No) | Yes | No |
|  | 322-336 | SKDLIAEIQKQGNDQ | 3.3 | HLA-DRB1*03:01  HLA-DRB1*15:01  HLA-DRB3*02:02  HLA-DRB4*01:01  HLA-DRB5*01:01 | 0.2226  (No) | No | No |
|  | 327-341 | AEIQKQGNDQWTFQF | 3.3 | HLA-DRB4*01:01 | 0.6864  (Yes) | Yes | No |
|  | 338-352 | TFQFYQEPFKNLKTG | 18 | HLA-DRB3*01:01 | 0.5019  (Yes) | No | No |
|  | **343-357** | **QEPFKNLKTGKFAKR** | **0.38** | **HLA-DRB1*07:01**  **HLA-DRB1*15:01**  **HLA-DRB3*01:01**  **HLA-DRB3*02:02**  **HLA-DRB4*01:01**  **HLA-DRB5*01:01** | **0.7494  (Yes)** | **No** | **No** |
|  | 358-372 | GTAHTNDVKQLTAVV | 13 | HLA-DRB1*03:01 | 0.4777  (Yes) | No | No |
|  | 365-379 | VKQLTAVVQKIALES | 18 | HLA-DRB1*15:01  HLA-DRB4*01:01 | 0.2977  (No) | Yes | No |
|  | 378-392 | ESIVIWGKTPKFRLP | 12 | HLA-DRB1*03:01  HLA-DRB1*07:01  HLA-DRB1*15:01  HLA-DRB3*01:01  HLA-DRB3*02:02  HLA-DRB5*01:01 | -0.2622  (No) | Yes | No |
|  | 411-425 | IPEWEFVNTPPLVKL | 1.2 | HLA-DRB1*07:01  HLA-DRB1*15:01  HLA-DRB3*01:01  HLA-DRB3*02:02  HLA-DRB5*01:01 | 0.5314  (Yes) | No | No |
|  | 423-437 | VKLWYQLEKEPIAGV | 8.4 | HLA-DRB1*07:01  HLA-DRB3*01:01  HLA-DRB3*02:02  HLA-DRB5*01:01 | 0.3019  (No) | No | No |
|  | 431-445 | KEPIAGVETFYVDGA | 0.48 | HLA-DRB1*03:01  HLA-DRB1*07:01  HLA-DRB1*15:01  HLA-DRB3*01:01  HLA-DRB3*02:02  HLA-DRB4*01:01  HLA-DRB5*01:01 | -0.0163  (No) | No | No |
|  | 437-451 | VETFYVDGAANRETK | 0.16 | HLA-DRB1*03:01  HLA-DRB1*07:01  HLA-DRB3*01:01  HLA-DRB3*02:02  HLA-DRB4*01:01  HLA-DRB5*01:01 | 0.1192  (No) | Yes | No |
|  | 454-468 | KAGYVTDRGRQKIVS | 0.32 | HLA-DRB1*03:01  HLA-DRB3*01:01  HLA-DRB3*02:02  HLA-DRB5*01:01 | 1.0049  (Yes) | No | No |
|  | 464-478 | QKIVSLTETTNQKTE | 7.5 | HLA-DRB1*07:01  HLA-DRB1*15:01  HLA-DRB3*02:02  HLA-DRB4*01:01  HLA-DRB5*01:01 | 0.9200  (Yes) | No | No |
|  | 469-483 | LTETTNQKTELQAIQ | 9 | HLA-DRB4*01:01 | 0.8284  (Yes) | No | No |
|  | 476-490 | KTELQAIQLALQDSG | 1.5 | HLA-DRB4*01:01 | 0.8752  (Yes) | Yes | No |
|  | 481-495 | AIQLALQDSGSEVNI | 9.6 | HLA-DRB3*01:01  HLA-DRB4*01:01 | 0.6623  (Yes) | No | No |
|  | 491-505 | SEVNIVTDSQYALGI | 0.41 | HLA-DRB1*03:01  HLA-DRB1*15:01  HLA-DRB3*01:01  HLA-DRB3*02:02  HLA-DRB4*01:01 | 0.7300  (Yes) | Yes | No |
|  | 502-516 | ALGIILAQPDKSESE | 0.06 | HLA-DRB1*03:01  HLA-DRB1*15:01  HLA-DRB3*02:02  HLA-DRB4*01:01  HLA-DRB5*01:01 | 0.5598  (Yes) | No | No |
|  | 514-528 | ESEIVNQIIEQLISK | 0.46 | HLA-DRB1*03:01  HLA-DRB1*07:01  HLA-DRB1*15:01  HLA-DRB3*01:01  HLA-DRB3*02:02  HLA-DRB4*01:01 | -0.4304  (No) | No | No |
|  | 519-533 | NQIIEQLISKERVYL | 1.1 | HLA-DRB1*15:01  HLA-DRB4*01:01  HLA-DRB5*01:01 | -0.2334  (No) | No | No |
|  | 528-542 | KERVYLSWVPAHKGI | 0.64 | HLA-DRB1*07:01  HLA-DRB1*15:01  HLA-DRB3*02:02  HLA-DRB4*01:01  HLA-DRB5*01:01 | 1.1709  (Yes) | Yes | No |
|  | 533-547 | LSWVPAHKGIGGNEQ | 20 | HLA-DRB5*01:01 | 1.2859  (Yes) | Yes | No |
|  | 539-553 | HKGIGGNEQVDKLVS | 12 | HLA-DRB1*03:01 | 0.1745  (No) | Yes | No |
|  | 546-560 | EQVDKLVSSGIRKVL | 8.6 | HLA-DRB1*07:01 | -0.2270  (No) | No | No |

Supplementary Table 17. Identified MHC-II epitope within integrase, rank, alleles, antigen, allergen, and toxic properties.

| **Sl.No** | **Position** | **Peptide** | **Rank** | **Alleles** | **Antigen** | **Allergen** | **Toxic** |
| --- | --- | --- | --- | --- | --- | --- | --- |
|  | 12-26 | HEKYHSNWRAMANEF | 2.8 | HLA-DRB1*03:01  HLA-DRB3*01:01  HLA-DRB3*02:02 | 0.2104 (No) | Yes | No |
|  | 17-31 | SNWRAMANEFNIPPV | 8.2 | HLA-DRB3*02:02  HLA-DRB5*01:01 | 0.6476 (Yes) | No | No |
|  | 23-37 | ANEFNIPPVVPKEIV | 9.1 | HLA-DRB3*02:02  HLA-DRB5*01:01 | 0.6454 (Yes) | Yes | No |
|  | 41-55 | DKCQLKGEAIHGQVN | 14 | HLA-DRB4*01:01 | 0.3929 (No) | Yes | No |
|  | 58-72 | PGIWQLDCTHLEGKI | 1.7 | HLA-DRB1*03:01  HLA-DRB3*01:01  HLA-DRB3*02:02 | 0.8023 (Yes) | No | No |
|  | 69-83 | EGKIILVAVHVASGY | 4 | HLA-DRB1*07:01  HLA-DRB1*15:01  HLA-DRB3*02:02  HLA-DRB4*01:01  HLA-DRB5*01:01 | 0.5749 (Yes) | No | No |
|  | 74-88 | LVAVHVASGYIEAEV | 16 | HLA-DRB1*07:01 | 0.6722 (Yes) | No | No |
|  | 81-95 | SGYIEAEVIPAETGQ | 4.1 | HLA-DRB1*03:01  HLA-DRB1*07:01  HLA-DRB3*01:01  HLA-DRB3*02:02  HLA-DRB4*01:01  HLA-DRB5*01:01 | 0.7613 (Yes) | Yes | No |
|  | 93-107 | TGQETAYFLLKLAGR | 19 | HLA-DRB5*01:01 | 0.7121 (Yes) | Yes | No |
|  | 98-112 | AYFLLKLAGRWPVRV | 20 | HLA-DRB1*03:01 | 0.5626 (Yes) | Yes | No |
|  | 105-119 | AGRWPVRVIHTDNGS | 0.14 | HLA-DRB4*01:01 | 0.4951 (Yes) | Yes | No |
|  | 110-124 | VRVIHTDNGSNFTSN | 0.14 | HLA-DRB1*03:01  HLA-DRB3*01:01  HLA-DRB3*02:02 | 0.3414 (No) | Yes | No |
|  | 117-131 | NGSNFTSNAVKAACW | 0.99 | HLA-DRB3*02:02 | 0.6193 (Yes) | No | No |
|  | 132-146 | WAGIQQEFGIPYNPQ | 6.7 | HLA-DRB1*03:01  HLA-DRB1*15:01  HLA-DRB3*01:01  HLA-DRB5*01:01 | 0.6756 (Yes) | No | No |
|  | 138-152 | EFGIPYNPQSQGVVE | 19 | HLA-DRB3*01:01  HLA-DRB3*02:02 | 0.7839 (Yes) | No | No |
|  | 147-161 | SQGVVESMNKELKKI | 4.5 | HLA-DRB1*03:01  HLA-DRB1*07:01  HLA-DRB1*15:01  HLA-DRB3*02:02  HLA-DRB5*01:01 | 0.1130 (No) | No | No |
|  | 152-166 | ESMNKELKKIIGQVR | 7.8 | HLA-DRB5*01:01 | -0.3100 (No) | Yes | No |
|  | 157-171 | ELKKIIGQVREQAEH | 1.7 | HLA-DRB1*03:01  HLA-DRB1*15:01  HLA-DRB3*02:02  HLA-DRB4*01:01  HLA-DRB5*01:01 | 0.1049 (No) | No | No |
|  | 162-176 | IGQVREQAEHLKTAV | 3.2 | HLA-DRB4*01:01  HLA-DRB5*01:01 | 0.5843 (Yes) | No | No |
|  | 178-192 | MAVFIHNFKRRGGIG | 7 | HLA-DRB1*03:01  HLA-DRB1*15:01  HLA-DRB3*02:02  HLA-DRB5*01:01 | 0.7114 (Yes) | Yes | No |
|  | 188-202 | RGGIGGYSAGERIID | 2.2 | HLA-DRB1*15:01  HLA-DRB5*01:01 | 0.5217 (Yes) | No | No |
|  | 196-210 | AGERIIDIIASDIQT | 0.76 | HLA-DRB1*03:01  HLA-DRB1*07:01  HLA-DRB1*15:01  HLA-DRB3*01:01  HLA-DRB3*02:02  HLA-DRB4*01:01  HLA-DRB5*01:01 | 0.2671 (No) | No | No |
|  | 201-215 | IDIIASDIQTKELQN | 1.4 | HLA-DRB1*03:01  HLA-DRB3*01:01  HLA-DRB4*01:01 | 0.8096 (Yes) | Yes | No |
|  | 208-222 | IQTKELQNQILKIQN | 5 | HLA-DRB3*02:02  HLA-DRB4*01:01 | 0.4935 (Yes) | No | No |
|  | 213-227 | LQNQILKIQNFRVYY | 0.13 | HLA-DRB1*07:01  HLA-DRB1*15:01  HLA-DRB3*01:01  HLA-DRB3*02:02  HLA-DRB4*01:01  HLA-DRB5*01:01 | -0.0305 (No) | No | No |
|  | 218-232 | LKIQNFRVYYRDSRD | 4.4 | HLA-DRB1*03:01  HLA-DRB1*15:01  HLA-DRB5*01:01 | 0.7125 (Yes) | No | No |
|  | 223-237 | FRVYYRDSRDPIWKG | 0.99 | HLA-DRB1*03:01  HLA-DRB3*01:01  HLA-DRB3*02:02 | 0.4101 (Yes) | No | No |
|  | 231-245 | RDPIWKGPAKLLWKG | 6.9 | HLA-DRB1*07:01  HLA-DRB1*15:01  HLA-DRB3*02:02  HLA-DRB5*01:01 | -0.0978 (No) | Yes | No |
|  | 240-254 | KLLWKGEGAVVIQDN | 2.8 | HLA-DRB1*07:01  HLA-DRB3*01:01  HLA-DRB3*02:02  HLA-DRB5*01:01 | 0.5305 (Yes) | Yes | No |
|  | 247-261 | GAVVIQDNSDIKVVP | 1.3 | HLA-DRB1*03:01  HLA-DRB1*07:01  HLA-DRB1*15:01  HLA-DRB3*01:01  HLA-DRB3*02:02  HLA-DRB4*01:01 | 0.9620 (Yes) | Yes | No |
|  | **253-267** | **DNSDIKVVPRRKAKI** | **4.2** | **HLA-DRB1*03:01**  **HLA-DRB1*15:01**  **HLA-DRB3*02:02**  **HLA-DRB4*01:01**  **HLA-DRB5*01:01** | **1.2710 (Yes)** | **No** | **No** |
|  | 264-278 | KAKIIRDYGKQMAGA | 0.27 | HLA-DRB1*03:01  HLA-DRB1*07:01  HLA-DRB1*15:01  HLA-DRB3*01:01  HLA-DRB3*02:02  HLA-DRB4*01:01  HLA-DRB5*01:01 | -0.8522 (No) | No | No |

**Supplementary Table 18.** List of mapped mutated B cell epitope of each target along with their reference position, antigen, allergen and toxicity features.

| Position | epitope | A.Pos | R.Pos | V.Amino acid | M.Epitope | Antigen | Allergen | Toxic |
| --- | --- | --- | --- | --- | --- | --- | --- | --- |
| **Envelope glycoprotein** | | | | | | | | |
| 78-93 | PSPQELGLENVTENFN | S79 | 89 | N/T | PNPQELGLENVTENFN | 1.1426 (Yes) | No | No |
|  |  |  |  |  | PTPQELGLENVTENFN | 1.0129 (Yes) | No | No |
|  |  | Q81 | 91 | Y/H/R | PSPYELGLENVTENFN | 1.0133 (Yes) | No | No |
|  |  |  |  |  | PSPHELGLENVTENFN | 0.8509 (Yes) | No | No |
|  |  |  |  |  | PSPRELGLENVTENFN | 1.0051 (Yes) | No | No |
|  |  | L83 | 93 | Y/F/V/I/M | PSPQEYGLENVTENFN | 0.7312 (Yes) | No | No |
|  |  |  |  |  | PSPQEFGLENVTENFN | 0.9152 (Yes) | No | No |
|  |  |  |  |  | PSPQEVGLENVTENFN | 0.9424 (Yes) | Yes | No |
|  |  |  |  |  | PSPQEIGLENVTENFN | 1.0267 (Yes) | No | No |
|  |  |  |  |  | PSPQEMGLENVTENFN | 0.8445 (Yes) | Yes | No |
|  |  | G84 | 94 | N/S/V/A/F/E/K/I/P | PSPQELNLENVTENFN | 1.0220 (Yes) | No | No |
|  |  |  |  |  | PSPQELSLENVTENFN | 0.9286 (Yes) | No | No |
|  |  |  |  |  | PSPQELVLENVTENFN | 0.4230 (Yes) | No | No |
|  |  |  |  |  | PSPQELALENVTENFN | 0.7238 (Yes) | No | No |
|  |  |  |  |  | PSPQELFLENVTENFN | 0.1634 (No) | No | No |
|  |  |  |  |  | PSPQELELENVTENFN | 1.0074 (Yes) | No | No |
|  |  |  |  |  | PSPQELKLENVTENFN | 0.9181 (Yes) | No | No |
|  |  |  |  |  | PSPQELILENVTENFN | 0.2316 (No) | No | No |
|  |  |  |  |  | PSPQELPLENVTENFN | 0.5865 (Yes) | No | No |
|  |  | L85 | 95 | M/I | PSPQELGMENVTENFN | 0.9327 (Yes) | No | No |
|  |  |  |  |  | PSPQELGIENVTENFN | 1.0073 (Yes) | No | No |
|  |  | E86 | 96 | P/G/K/V/Q/T/H | PSPQELGLPNVTENFN | 0.9462 (Yes) | No | No |
|  |  |  |  |  | **PSPQELGLGNVTENFN** | **1.4187 (Yes)** | **No** | **No** |
|  |  |  |  |  | PSPQELGLKNVTENFN | 1.0523 (Yes) | No | No |
|  |  |  |  |  | PSPQELGLVNVTENFN | 1.3492 (Yes) | No | No |
|  |  |  |  |  | PSPQELGLQNVTENFN | 1.0493 (Yes) | No | No |
|  |  |  |  |  | PSPQELGLTNVTENFN | 1.2673 (Yes) | No | No |
|  |  |  |  |  | PSPQELGLHNVTENFN | 0.8724 (Yes) | No | No |
|  |  | T89 | 99 | S | PSPQELGLENVSENFN | 1.0771 (Yes) | No | No |
|  |  | E90 | 100 | D | PSPQELGLENVTDNFN | 0.9929 (Yes) | No | No |
|  |  | N91 | 101 | D/K/Y/G/S/T/R | PSPQELGLENVTEDFN | 1.0419 (Yes) | No | No |
|  |  |  |  |  | PSPQELGLENVTEKFN | 0.9501 (Yes) | No | No |
|  |  |  |  |  | PSPQELGLENVTEYFN | 0.8309 (Yes) | No | No |
|  |  |  |  |  | PSPQELGLENVTEGFN | 1.1182 (Yes) | No | No |
|  |  |  |  |  | PSPQELGLENVTESFN | 1.0302 (Yes) | No | No |
|  |  |  |  |  | PSPQELGLENVTETFN | 0.9859 (Yes) | No | No |
|  |  |  |  |  | PSPQELGLENVTERFN | 0.9236 (Yes) | No | No |
|  |  | N93 | 103 | D | PSPQELGLENVTENFD | 0.9998 (Yes) | No | No |
| **Protease** | | | | | | | | |
| 54-69 | IKVRQYDQIIIEICGK | R57 | 57 | K | IKVKQYDQIIIEICGK | 0.5572 (Yes) | Yes | No |
|  |  | Q58 | 58 | E | IKVREYDQIIIEICGK | 0.6094 (Yes) | No | No |
|  |  | D60 | 60 | N/E | IKVRQYNQIIIEICGK | 0.4176 (Yes) | Yes | No |
|  |  |  |  |  | IKVRQYEQIIIEICGK | 0.3471 (No) | No | No |
|  |  | Q61 | 61 | N/E/S | IKVRQYDNIIIEICGK | 0.6045 (Yes) | Yes | No |
|  |  |  |  |  | IKVRQYDEIIIEICGK | 0.5258 (Yes) | Yes | No |
|  |  |  |  |  | IKVRQYDSIIIEICGK | 0.5608 (Yes) | Yes | No |
|  |  | I62 | 62 | V | IKVRQYDQVIIEICGK | 0.6010 (Yes) | No | No |
|  |  | I63 | 63 | T/P/A/L/C/S/D | IKVRQYDQITIEICGK | 0.7111 (Yes) | Yes | No |
|  |  |  |  |  | IKVRQYDQIPIEICGK | 0.9010 (Yes) | Yes | No |
|  |  |  |  |  | IKVRQYDQIAIEICGK | 0.7968 (Yes) | Yes | No |
|  |  |  |  |  | IKVRQYDQILIEICGK | 0.5510 (Yes) | Yes | No |
|  |  |  |  |  | **IKVRQYDQICIEICGK** | **1.1378 (Yes)** | **No** | **No** |
|  |  |  |  |  | IKVRQYDQISIEICGK | 0.9031 (Yes) | Yes | No |
|  |  |  |  |  | IKVRQYDQIDIEICGK | 1.0858 (Yes) | Yes | No |
|  |  | I64 | 64 | V/M | IKVRQYDQIIVEICGK | 0.5228 (Yes) | Yes | No |
|  |  |  |  |  | IKVRQYDQIIMEICGK | 0.5115 (Yes) | No | No |
|  |  | E65 | 65 | D/G | IKVRQYDQIIIDICGK | 0.4203 (Yes) | No | No |
|  |  |  |  |  | IKVRQYDQIIIGICGK | 1.0225 (Yes) | Yes | No |
|  |  | I66 | 66 | V | IKVRQYDQIIIEVCGK | 0.4557 (Yes) | No | No |
|  |  | C67 | 67 | E/Q/F/S/G | IKVRQYDQIIIEIEGK | 1.1810 (Yes) | Yes | No |
|  |  |  |  |  | IKVRQYDQIIIEIQGK | 1.2364 (Yes) | Yes | No |
|  |  |  |  |  | IKVRQYDQIIIEIFGK | 0.5156 (Yes) | Yes | No |
|  |  |  |  |  | IKVRQYDQIIIEISGK | 1.0597 (Yes) | Yes | No |
|  |  |  |  |  | IKVRQYDQIIIEIGGK | 1.1764 (Yes) | Yes | No |
|  |  | K69 | 69 | R/Q/H/Y | IKVRQYDQIIIEICGR | 0.5595 (Yes) | Yes | No |
|  |  |  |  |  | IKVRQYDQIIIEICGQ | 0.3580 (No) | Yes | No |
|  |  |  |  |  | IKVRQYDQIIIEICGH | 0.1174 (No) | No | No |
|  |  |  |  |  | IKVRQYDQIIIEICGY | 0.2450 (No) | Yes | No |
| **Reverse transcriptase** | | | | | | | | |
| 349-364 | LKTGKFAKRGTAHTND | F354 | 354 | Y | **LKTGKYAKRGTAHTND** | **1.1961 (Yes)** | **No** | **No** |
|  |  | A355 | 355 | T | LKTGKFTKRGTAHTND | 1.2156 (Yes) | Yes | No |
|  |  | K356 | 356 | R | LKTGKFARRGTAHTND | 1.1907 (Yes) | No | No |
|  |  | R357 | 357 | Q/M/K/T/V/I | LKTGKFAKQGTAHTND | 0.9435 (Yes) | No | No |
|  |  |  |  |  | LKTGKFAKMGTAHTND | 0.9645 (Yes) | No | Yes |
|  |  |  |  |  | LKTGKFAKKGTAHTND | 1.1457 (Yes) | No | No |
|  |  |  |  |  | LKTGKFAKTGTAHTND | 0.9895 (Yes) | No | No |
|  |  |  |  |  | LKTGKFAKVGTAHTND | 1.0548 (Yes) | No | No |
|  |  |  |  |  | LKTGKFAKIGTAHTND | 1.0682 (Yes) | No | No |
|  |  | G358 | 358 | K/R | LKTGKFAKRKTAHTND | 0.7950 (Yes) | No | No |
|  |  |  |  |  | LKTGKFAKRRTAHTND | 0.7367 (Yes) | Yes | No |
|  |  | T359 | 359 | A/S/G | LKTGKFAKRGAAHTND | 1.1165 (Yes) | No | No |
|  |  |  |  |  | LKTGKFAKRGSAHTND | 1.0936 (Yes) | No | No |
|  |  |  |  |  | LKTGKFAKRGGAHTND | 1.1427 (Yes) | No | No |
|  |  | A360 | 360 | S/T | LKTGKFAKRGTSHTND | 1.1877 (Yes) | Yes | No |
|  |  |  |  |  | LKTGKFAKRGTTHTND | 1.1834 (Yes) | Yes | No |
|  |  |  |  |  |  |  |  |  |
| **Integrase** | | | | | | | | |
| 188-203 | RGGIGGYSAGERIIDI | R188 | 188 | K | KGGIGGYSAGERIIDI | 0.7712 (Yes) | No | No |
|  |  | G189 | 189 | R | RRGIGGYSAGERIIDI | 0.4615 (Yes) | No | No |
|  |  | T195 | 195 | T | RGGIGGYTAGERIIDI | 0.8323 (Yes) | No | No |
|  |  | R197 | 197 | R | **RGGIGGYSARERIIDI** | **1.5032 (Yes)** | **No** | **No** |
|  |  | I200 | 200 | L/T | RGGIGGYSAGERLIDI | 0.8055 (Yes) | No | No |
|  |  |  |  |  | RGGIGGYSAGERTIDI | 1.0723 (Yes) | No | No |
|  |  | I201 | 201 | V | RGGIGGYSAGERIVDI | 0.8486 (Yes) | No | No |
|  |  | D202 | 202 | G | RGGIGGYSAGERIIGI | 0.5806 (Yes) | No | No |
|  |  | I203 | 203 | M | RGGIGGYSAGERIIDM | 0.6878 (Yes) | No | No |

**Supplementary Table 19.** List of mapped mutated MHC-I and II envelop glycoprotein epitope along with their reference position, antigen, allergen and toxicity features.

| Position | epitope | Abs. p | Rel. p | V.Amino acid | M.Epitope | Antigen | Allergen | Toxic |
| --- | --- | --- | --- | --- | --- | --- | --- | --- |
| **MHC-I** | | | | | | | | |
| 206-214 | SLDPIPIHY | S206 | 237 | N/T | **NLDPIPIHY** | **2.4487 (Yes)** | **No** | **No** |
|  |  |  |  |  | TLDPIPIHY | 1.9528 (Yes) | No | No |
|  |  | L207 | 238 | F/W | SFDPIPIHY | 2.2279 (Yes) | Yes | No |
|  |  |  |  |  | SWDPIPIHY | 2.2977 (Yes) | Yes | No |
|  |  | D208 | 239 | Q/E | SLQPIPIHY | 1.9155 (Yes) | No | No |
|  |  |  |  |  | SLEPIPIHY | 2.0264 (Yes) | No | No |
|  |  | I212 | 243 | M | SLDPIPMHY | 1.8067 (Yes) | No | No |
|  |  | Y214 | 245 | F | SLDPIPIHF | 2.2814 (Yes) | No | No |
| **MHC-II** | | | | | | | | |
| 351-365 | NKTIEFKPSSGGDLE | N351 | 390 | Q/K/F | QKTIEFKPSSGGDLE | 1.2999 (Yes) | No | No |
|  |  |  |  |  | KKTIEFKPSSGGDLE | 1.2956 (Yes) | No | No |
|  |  |  |  |  | FKTIEFKPSSGGDLE | 1.3326 (Yes) | No | No |
|  |  | K352 | 391 | T/N/R/S/A/G | NTTIEFKPSSGGDLE | 1.5342 (Yes) | No | No |
|  |  |  |  |  | NNTIEFKPSSGGDLE | 1.2552 (Yes) | No | No |
|  |  |  |  |  | NRTIEFKPSSGGDLE | 1.2765 (Yes) | No | No |
|  |  |  |  |  | NSTIEFKPSSGGDLE | 1.4401 (Yes) | No | No |
|  |  |  |  |  | NATIEFKPSSGGDLE | 1.5553 (Yes) | No | No |
|  |  |  |  |  | NGTIEFKPSSGGDLE | 1.5616 (Yes) | No | No |
|  |  | T353 | 395 | N/S/K/I/A/D | NKNIEFKPSSGGDLE | 1.5516 (Yes) | No | No |
|  |  |  |  |  | NKSIEFKPSSGGDLE | 1.3882 (Yes) | No | No |
|  |  |  |  |  | NKKIEFKPSSGGDLE | 1.5768 (Yes) | No | No |
|  |  |  |  |  | NKIIEFKPSSGGDLE | 0.8316 (Yes) | No | No |
|  |  |  |  |  | NKAIEFKPSSGGDLE | 1.2225 (Yes) | No | No |
|  |  |  |  |  | NKDIEFKPSSGGDLE | 1.5611 (Yes) | Yes | No |
|  |  | I354 | 396 | M/F/V/T | NKTMEFKPSSGGDLE | 1.1610 (Yes) | No | No |
|  |  |  |  |  | NKTFEFKPSSGGDLE | 1.2559 (Yes) | No | No |
|  |  |  |  |  | NKTVEFKPSSGGDLE | 1.2330 (Yes) | No | No |
|  |  |  |  |  | NKTTEFKPSSGGDLE | 1.0158 (Yes) | No | No |
|  |  | E355 | 397 | T/I/R/K/V/A/N/D/S/G/F | NKTITFKPSSGGDLE | 0.9263 (Yes) | No | No |
|  |  |  |  |  | NKTIIFKPSSGGDLE | 0.3356 (No) | No | No |
|  |  |  |  |  | NKTIRFKPSSGGDLE | 1.0551 (Yes) | No | No |
|  |  |  |  |  | NKTIKFKPSSGGDLE | 1.0594 (Yes) | No | No |
|  |  |  |  |  | NKTIVFKPSSGGDLE | 0.5081 (Yes) | No | No |
|  |  |  |  |  | NKTIAFKPSSGGDLE | 0.9494 (Yes) | No | No |
|  |  |  |  |  | NKTINFKPSSGGDLE | 1.4179 (Yes) | No | No |
|  |  |  |  |  | NKTIDFKPSSGGDLE | 1.5825 (Yes) | No | No |
|  |  |  |  |  | NKTISFKPSSGGDLE | 1.2090 (Yes) | No | No |
|  |  |  |  |  | NKTIGFKPSSGGDLE | 1.1699 (Yes) | No | No |
|  |  |  |  |  | NKTIFFKPSSGGDLE | 0.4357 (Yes) | No | No |
|  |  | F356 | 398 | A/L/K | NKTIEAKPSSGGDLE | 0.8739 (Yes) | No | No |
|  |  |  |  |  | NKTIELKPSSGGDLE | 1.2125 (Yes) | No | No |
|  |  |  |  |  | NKTIEKKPSSGGDLE | 0.6272 (Yes) | No | No |
|  |  | K357 | 399 | N/S/R/Q/T/D/A/P | NKTIEFNPSSGGDLE | 1.3180 (Yes) | No | No |
|  |  |  |  |  | NKTIEFSPSSGGDLE | 1.2913 (Yes) | No | No |
|  |  |  |  |  | NKTIEFRPSSGGDLE | 1.3027 (Yes) | No | No |
|  |  |  |  |  | NKTIEFQPSSGGDLE | 1.2798 (Yes) | No | No |
|  |  |  |  |  | NKTIEFTPSSGGDLE | 1.2460 (Yes) | No | No |
|  |  |  |  |  | NKTIEFDPSSGGDLE | 1.3404 (Yes) | No | No |
|  |  |  |  |  | NKTIEFAPSSGGDLE | 1.1820 (Yes) | No | No |
|  |  |  |  |  | NKTIEFPPSSGGDLE | 1.0536 (Yes) | No | No |
|  |  | P358 | 400 | H/R/E/D/S/Q/K/N | NKTIEFKHSSGGDLE | 1.1926 (Yes) | Yes | No |
|  |  |  |  |  | NKTIEFKRSSGGDLE | 0.8213 (Yes) | Yes | No |
|  |  |  |  |  | NKTIEFKESSGGDLE | 1.0550 (Yes) | No | No |
|  |  |  |  |  | NKTIEFKDSSGGDLE | 1.2679 (Yes) | No | No |
|  |  |  |  |  | NKTIEFKSSSGGDLE | 1.0793 (Yes) | Yes | No |
|  |  |  |  |  | NKTIEFKQSSGGDLE | 0.9801 (Yes) | Yes | No |
|  |  |  |  |  | NKTIEFKKSSGGDLE | 0.8227 (Yes) | Yes | No |
|  |  |  |  |  | NKTIEFKNSSGGDLE | 1.1528 (Yes) | No | No |
|  |  | S359 | 401 | T/R/K/P/H/N | NKTIEFKPTSGGDLE | 1.5856 (Yes) | No | No |
|  |  |  |  |  | NKTIEFKPRSGGDLE | 1.6848 (Yes) | Yes | No |
|  |  |  |  |  | **NKTIEFKPKSGGDLE** | **1.6779 (Yes)** | **No** | **No** |
|  |  |  |  |  | NKTIEFKPPSGGDLE | 1.0935 (Yes) | No | No |
|  |  |  |  |  | NKTIEFKPHSGGDLE | 1.1537 (Yes) | No | No |
|  |  |  |  |  | NKTIEFKPNSGGDLE | 1.1824 (Yes) | No | No |
|  |  | S360 | 402 | N/A/P | NKTIEFKPSNGGDLE | 1.4096 (Yes) | No | No |
|  |  |  |  |  | NKTIEFKPSAGGDLE | 1.2447 (Yes) | No | No |
|  |  |  |  |  | NKTIEFKPSPGGDLE | 1.2425 (Yes) | No | No |
|  |  | G361 | 403 | E | NKTIEFKPSSEGDLE | 1.3662 (Yes) | Yes | No |
|  |  | L364 | 406 | A/I/P/M/V | NKTIEFKPSSGGDAE | 1.1915 (Yes) | No | No |
|  |  |  |  |  | NKTIEFKPSSGGDIE | 1.3187 (Yes) | No | No |
|  |  |  |  |  | NKTIEFKPSSGGDPE | 1.2023 (Yes) | No | No |
|  |  |  |  |  | NKTIEFKPSSGGDME | 1.2702 (Yes) | No | No |
|  |  |  |  |  | NKTIEFKPSSGGDVE | 1.2760 (Yes) | No | No |
|  |  | E365 | 407 | Q | NKTIEFKPSSGGDLQ | 1.2974 (Yes) | No | No |

**Supplementary Table 20.** List of mapped mutated MHC-I and II protease epitopes along with their reference position, antigen, allergen and toxicity features.

| Position | epitope | Abs. p | Rel. p | V.Amino acid | M.Epi | Antigen | Allergen | Toxic |
| --- | --- | --- | --- | --- | --- | --- | --- | --- |
| **MHC-I** | | | | | | | | |
| 91-99 | TQLGRTLNF | Q92 | 92 | G | TGLGRTLNF | 1.1084(Yes) | No | No |
|  |  | L93 | 93 | I/M | **TQIGRTLNF** | **1.3254(Yes)** | **No** | **No** |
|  |  |  |  |  | TQMGRTLNF | 1.2781(Yes) | No | No |
|  |  | 95 | 95 | C | TQLGCTLNF | 1.0833(Yes) | Yes | No |
|  |  | 98 | 98 | H | TQLGRTLHF | 1.2660(Yes) | No | No |
|  |  | 99 | 99 | L | TQLGRTLNL | 1.0407(Yes) | No | No |
| **MHC II** | | | | | | | | |
| 42-56 | WKPKMIGGIGGFIKV | K43 | 43 | T | WTPKMIGGIGGFIKV | 0.3930 (No) | No | No |
|  |  | M46 | 46 | I | **WKPKIIGGIGGFIKV** | **0.5336(Yes)** | **No** | **No** |

**Supplementary Table 21.** List of mapped mutated MHC-I and II reverse transcriptase epitopes along with their reference position, antigen, allergen and toxicity features.

| Position | epitope | Abs. p | Rel. p | V.Amino acid | M.Epi | Antigen | Allergen | Toxic |
| --- | --- | --- | --- | --- | --- | --- | --- | --- |
| **MHC-I** | | | | | | | | |
| 381-389 | VIWGKTPKF | V381 | 381 | I | IIWGKTPKF | 0.5096 (Yes) | No | No |
|  |  | T386 | 387 | L/I/S/V | VIWGKLPKF | -0.1759(No) | No | No |
|  |  |  |  |  | VIWGKIPKF | -0.3028(No) | Yes | No |
|  |  |  |  |  | **VIWGKSPKF** | **0.5451 (Yes)** | **No** | **No** |
|  |  |  |  |  | VIWGKVPKF | -0.1548(No) | Yes | No |
| **MHC-II** | | | | | | | | |
| 343-357 | QEPFKNLKTGKFAKR | E344 | 344 | N/D | QNPFKNLKTGKFAKR | 0.7481 (Yes) | No | No |
|  |  |  |  |  | QDPFKNLKTGKFAKR | 0.7360 (Yes) | No | No |
|  |  | P345 | 345 | E/L/Q | **QEEFKNLKTGKFAKR** | **0.9871 (Yes)** | **No** | **No** |
|  |  |  |  |  | QELFKNLKTGKFAKR | 0.4226 (Yes) | No | No |
|  |  |  |  |  | QEQFKNLKTGKFAKR | 0.8824 (Yes) | No | No |
|  |  | F346 | 346 | Y/H | QEPYKNLKTGKFAKR | 0.7783 (Yes) | No | Yes |
|  |  |  |  |  | QEPHKNLKTGKFAKR | 0.8209 (Yes) | No | No |
|  |  | F354 | 354 | Y | QEPFKNLKTGKYAKR | 0.7861 (Yes) | No | No |
|  |  | A355 | 355 | T | QEPFKNLKTGKFAKR | 0.7494 (Yes) | No | No |
|  |  | K356 | 356 | R | QEPFKNLKTGKFARR | 0.7700 (Yes) | No | No |
|  |  | R357 | 357 | Q/M/K/T/V/I | QEPFKNLKTGKFAKQ | 0.5028 (Yes) | No | No |
|  |  |  |  |  | QEPFKNLKTGKFAKM | 0.4130 (Yes) | No | No |
|  |  |  |  |  | QEPFKNLKTGKFAKK | 0.6967 (Yes) | No | No |
|  |  |  |  |  | QEPFKNLKTGKFAKT | 0.4596 (Yes) | No | No |
|  |  |  |  |  | QEPFKNLKTGKFAKV | 0.4334 (Yes) | No | No |
|  |  |  |  |  | QEPFKNLKTGKFAKI | 0.4287 (Yes) | Yes | No |

**Supplementary Table 22.** List of mapped mutated MHC-I and II integrase epitopes along with their reference position, antigen, allergen and toxicity features.

| Position | epitope | Abs. p | Rel. p | V.Amino acid | M.Epitope | Antigen | Allergen | Toxic |
| --- | --- | --- | --- | --- | --- | --- | --- | --- |
| **MHC -I** | | | | | | | | |
| 75-83 | VAVHVASGY | G82 | 82 | D/E | VAVHVASDY | 0.2008 (No) | No | No |
|  |  |  |  |  | VAVHVASEY | 0.4220 (Yes) | No | No |
|  |  | Y83 | 83 | F | **VAVHVASGF** | **0.5744 (Yes)** | **No** | **No** |
| **MHC-II** | | | | | | | | |
| 253-267 | DNSDIKVVPRRKAKI | N254 | 254 | K | DKSDIKVVPRRKAKI | 1.3237 (Yes) | Yes | No |
|  |  | S255 | 255 | G/N | DNGDIKVVPRRKAKI | 1.1683 (Yes) | No | No |
|  |  |  |  |  | **DNNDIKVVPRRKAKI** | **1.2852 (Yes)** | **No** | **No** |
|  |  | D256 | 256 | E | DNSEIKVVPRRKAKI | 1.1532 (Yes) | No | No |
|  |  | V259 | 259 | I | DNSDIKIVPRRKAKI | 1.4711 (Yes) | Yes | No |
|  |  | V260 | 260 | V | DNSDIKVIPRRKAKI | 1.0684 (Yes) | No | No |
|  |  | K264 | 264 | E | DNSDIKVVPRREAKI | 1.1573 (Yes) | Yes | No |
|  |  | A265 | 265 | V | DNSDIKVVPRRKVKI | 1.1722 (Yes) | No | No |
|  |  | I267 | 267 | V | DNSDIKVVPRRKAKV | 1.2140 (Yes) | Yes | No |

Supplementary Table 23. List of vaccine construct along with antigen and allerg activity.

| Sl.No. | Vaccine construct | Antigen | Allergen |
| --- | --- | --- | --- |
|  | V1 | 0.6899 (Yes) | No |
|  | V2 | 0.7657 (Yes) | No |
|  | V3 | 0.7386 (Yes) | No |
|  | V4 | 0.7514 (Yes) | No |
|  | V5 | 0.7392 (Yes) | No |
|  | V6 | 0.7390 (Yes) | No |

Supplementary Table 24. Computed antigen, allergen, physicochemical and solubility properties of the non-mutated vaccine.

| **Sl.No** | **Properties** | **Non-Mutated vaccine** |
| --- | --- | --- |
|  | Antigen | 0.7657  ( Probable ANTIGEN ) |
|  | Allergen | Non-allergen |
|  | Residue count | 276 |
|  | Molecular weight | 30008.89 |
|  | Theoretical pI | 10.15 |
|  | Formula | C_1345_H_2158_N_402_O_362_S_8_ |
|  | Estimated half-life | 30 hours (mammalian reticulocytes, in vitro).  >20 hours (yeast, in vivo).  >10 hours (Escherichia coli, in vivo). |
|  | Instability index | 31.52 |
|  | Aliphatic index | 70.43 |
|  | Grand average of hydropathicity (GRAVY) | -0.557 |
|  | Solubility | 0.654 (Higher than scaled solubility) |

**Supplementary Table 25.** list of enhance non-mutated vaccine model with their attributes.

| **Model** | **GDT-HA** | **RMSD** | **MolProbity** | **Clash score** | **Poor rotamers** | **Rama favored** |
| --- | --- | --- | --- | --- | --- | --- |
| Initial | 1.0000 | 0.000 | 1.463 | 1.7 | 0.0 | 90.5 |
| MODEL 1 | 0.9855 | 0.328 | 2.244 | 14.1 | 1.4 | 92.3 |
| MODEL 2 | 0.9846 | 0.333 | 2.129 | 14.1 | 0.5 | 92.3 |
| **MODEL 3** | **0.9810** | **0.328** | **2.050** | **11.1** | **0.5** | **92.0** |
| MODEL 4 | 0.9810 | 0.334 | 2.109 | 13.4 | 0.9 | 92.3 |
| MODEL 5 | 0.9774 | 0.343 | 2.155 | 14.1 | 0.5 | 91.6 |

**Supplementary Table 26.** List of obtained discontinuous epitope within the mutated vaccine.

| Sl.No | Residues | Number of residues | Score |
| --- | --- | --- | --- |
|  | A:G1, A:I2, A:I3, A:N4, A:T5, A:L6, A:Q7, A:K8, A:Y9, A:Y10, A:C11, A:R12, A:V13, A:R14, A:G15, A:G16, A:C18, A:A19, A:V20, A:L21, A:S22, A:C23, A:L24, A:P25, A:K26, A:E27, A:E28, A:Q29, A:I30, A:G31, A:K32, A:C33, A:S34, A:T35, A:R36, A:G37, A:R38, A:K39, A:C40, A:C41, A:R42, A:R43, A:E46 | 43 | 0.815 |
|  | A:P186, A:F187, A:K188, A:N189, A:L190, A:K191, A:T192, A:G193, A:K194, A:F195, A:A196, A:K197 | 12 | 0.754 |
|  | A:E98, A:I99, A:C100, A:G101, A:K102, A:K103, A:A116, A:H117, A:T118, A:N119, A:D120, A:K121, A:K122, A:R123, A:G124, A:G125, A:I126, A:G127, A:G128, A:P200, A:G201, A:P202, A:G203, A:D204, A:N205, A:S206, A:D207, A:I208, A:K209 | 29 | 0.709 |
|  | A:G139, A:P140, A:G141, A:P142, A:G143, A:N144, A:K145, A:R267, A:R270, A:H271, A:H272, A:H273, A:H274, A:H275, A:H276 | 15 | 0.69 |
|  | A:S222, A:L223, A:D224, A:P225, A:I226 | 5 | 0.683 |
|  | A:A63, A:E64, A:A65, A:A66, A:A67, A:K68, A:P69, A:S70, A:P71, A:Q72, A:E73, A:L74, A:G75, A:L76, A:E77 | 15 | 0.658 |
|  | A:Y92, A:D93, A:Q94, A:I95, A:I96, A:I97, A:K104, A:L105, A:K106, A:T107, A:G108, A:K109, A:R113, A:V210, A:V211, A:P212, A:R213, A:R214, A:K215, A:A216 | 20 | 0.618 |

**Supplementary Table 27.** List of docked models with their respective details.

| Cluster | Members | Representative | Weighted Score |
| --- | --- | --- | --- |
| 0 | 55 | Center | -987.7 |
|  |  | Lowest Energy | -1026.8 |
| 1 | 46 | Center | -830.1 |
|  |  | Lowest Energy | -907.3 |
| 2 | 46 | Center | -1031.1 |
|  |  | Lowest Energy | -1031.1 |
| 3 | 42 | Center | -947.2 |
|  |  | Lowest Energy | -1001.8 |
| 4 | 42 | Center | -1021.4 |
|  |  | Lowest Energy | -1021.4 |
| 5 | 35 | Center | -887.4 |
|  |  | Lowest Energy | -888.2 |
| **6** | **30** | **Center** | **-930.3** |
|  |  | **Lowest Energy** | **-1120.2** |
| 7 | 30 | Center | -889.3 |
|  |  | Lowest Energy | -910.9 |
| 8 | 30 | Center | -844.5 |
|  |  | Lowest Energy | -997.0 |
| 9 | 28 | Center | -841.8 |
|  |  | Lowest Energy | -973.8 |
| 10 | 24 | Center | -819.8 |
|  |  | Lowest Energy | -984.9 |
| 11 | 24 | Center | -950.6 |
|  |  | Lowest Energy | -1018.4 |
| 12 | 22 | Center | -892.5 |
|  |  | Lowest Energy | -897.2 |
| 13 | 22 | Center | -879.6 |
|  |  | Lowest Energy | -879.6 |
| 14 | 21 | Center | -822.2 |
|  |  | Lowest Energy | -903.6 |
| 15 | 20 | Center | -871.1 |
|  |  | Lowest Energy | -887.5 |
| 16 | 18 | Center | -904.2 |
|  |  | Lowest Energy | -992.7 |
| 17 | 17 | Center | -813.1 |
|  |  | Lowest Energy | -909.1 |
| 18 | 17 | Center | -951.6 |
|  |  | Lowest Energy | -951.6 |
| 19 | 16 | Center | -832.9 |
|  |  | Lowest Energy | -983.1 |
| 20 | 16 | Center | -826.0 |
|  |  | Lowest Energy | -943.2 |
| 21 | 15 | Center | -804.0 |
|  |  | Lowest Energy | -924.7 |
| 22 | 14 | Center | -804.3 |
|  |  | Lowest Energy | -951.6 |
| 23 | 14 | Center | -906.3 |
|  |  | Lowest Energy | -911.1 |
| 24 | 14 | Center | -870.8 |
|  |  | Lowest Energy | -913.8 |
| 25 | 14 | Center | -843.4 |
|  |  | Lowest Energy | -843.4 |
| 26 | 13 | Center | -853.8 |
|  |  | Lowest Energy | -897.0 |
| 27 | 13 | Center | -804.2 |
|  |  | Lowest Energy | -1035.6 |
| 28 | 13 | Center | -932.2 |
|  |  | Lowest Energy | -932.2 |
| 29 | 12 | Center | -833.2 |
|  |  | Lowest Energy | -895.9 |

**Supplementary Table 28.** List of docked mutated with TLR3 model with their respective details.

| Cluster | Members | Representative | Weighted Score |
| --- | --- | --- | --- |
| 0 | 96 | Center | -920.9 |
|  |  | Lowest Energy | -1049.3 |
| 1 | 40 | Center | -1034.8 |
|  |  | Lowest Energy | -1034.8 |
| 2 | 38 | Center | -882.6 |
|  |  | Lowest Energy | -1127.9 |
| 3 | 36 | Center | -949.4 |
|  |  | Lowest Energy | -952.0 |
| 4 | 31 | Center | -848.5 |
|  |  | Lowest Energy | -1047.1 |
| 5 | 31 | Center | -951.4 |
|  |  | Lowest Energy | -1115.2 |
| 6 | 29 | Center | -982.0 |
|  |  | Lowest Energy | -982.0 |
| **7** | **28** | **Center** | **-1039.6** |
|  |  | **Lowest Energy** | **-1275.4** |
| 8 | 25 | Center | -905.0 |
|  |  | Lowest Energy | -967.4 |
| 9 | 24 | Center | -955.9 |
|  |  | Lowest Energy | -1042.3 |
| 10 | 24 | Center | -871.3 |
|  |  | Lowest Energy | -945.2 |
| 11 | 21 | Center | -936.0 |
|  |  | Lowest Energy | -1009.3 |
| 12 | 21 | Center | -911.5 |
|  |  | Lowest Energy | -1008.2 |
| 13 | 21 | Center | -931.9 |
|  |  | Lowest Energy | -931.9 |
| 14 | 20 | Center | -937.8 |
|  |  | Lowest Energy | -957.1 |
| 15 | 18 | Center | -880.8 |
|  |  | Lowest Energy | -926.7 |
| 16 | 18 | Center | -1052.4 |
|  |  | Lowest Energy | -1052.4 |
| 17 | 16 | Center | -1116.1 |
|  |  | Lowest Energy | -1123.9 |
| 18 | 16 | Center | -880.0 |
|  |  | Lowest Energy | -972.7 |
| 19 | 15 | Center | -962.5 |
|  |  | Lowest Energy | -962.5 |
| 20 | 14 | Center | -960.2 |
|  |  | Lowest Energy | -981.9 |
| 21 | 13 | Center | -902.8 |
|  |  | Lowest Energy | -925.0 |
| 22 | 13 | Center | -889.6 |
|  |  | Lowest Energy | -1066.6 |
| 23 | 13 | Center | -945.3 |
|  |  | Lowest Energy | -945.3 |
| 24 | 12 | Center | -842.8 |
|  |  | Lowest Energy | -959.3 |
| 25 | 12 | Center | -910.6 |
|  |  | Lowest Energy | -929.2 |
| 26 | 12 | Center | -1001.4 |
|  |  | Lowest Energy | -1014.2 |
| 27 | 12 | Center | -958.2 |
|  |  | Lowest Energy | -1053.7 |
| 28 | 12 | Center | -900.5 |
|  |  | Lowest Energy | -951.2 |
| 29 | 11 | Center | -976.3 |
|  |  | Lowest Energy | -976.3 |


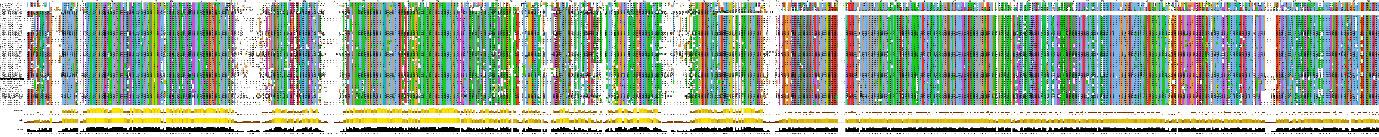


**Supplementary Figure 1.** The sequence alignment illustration of envelop glycoprotein.


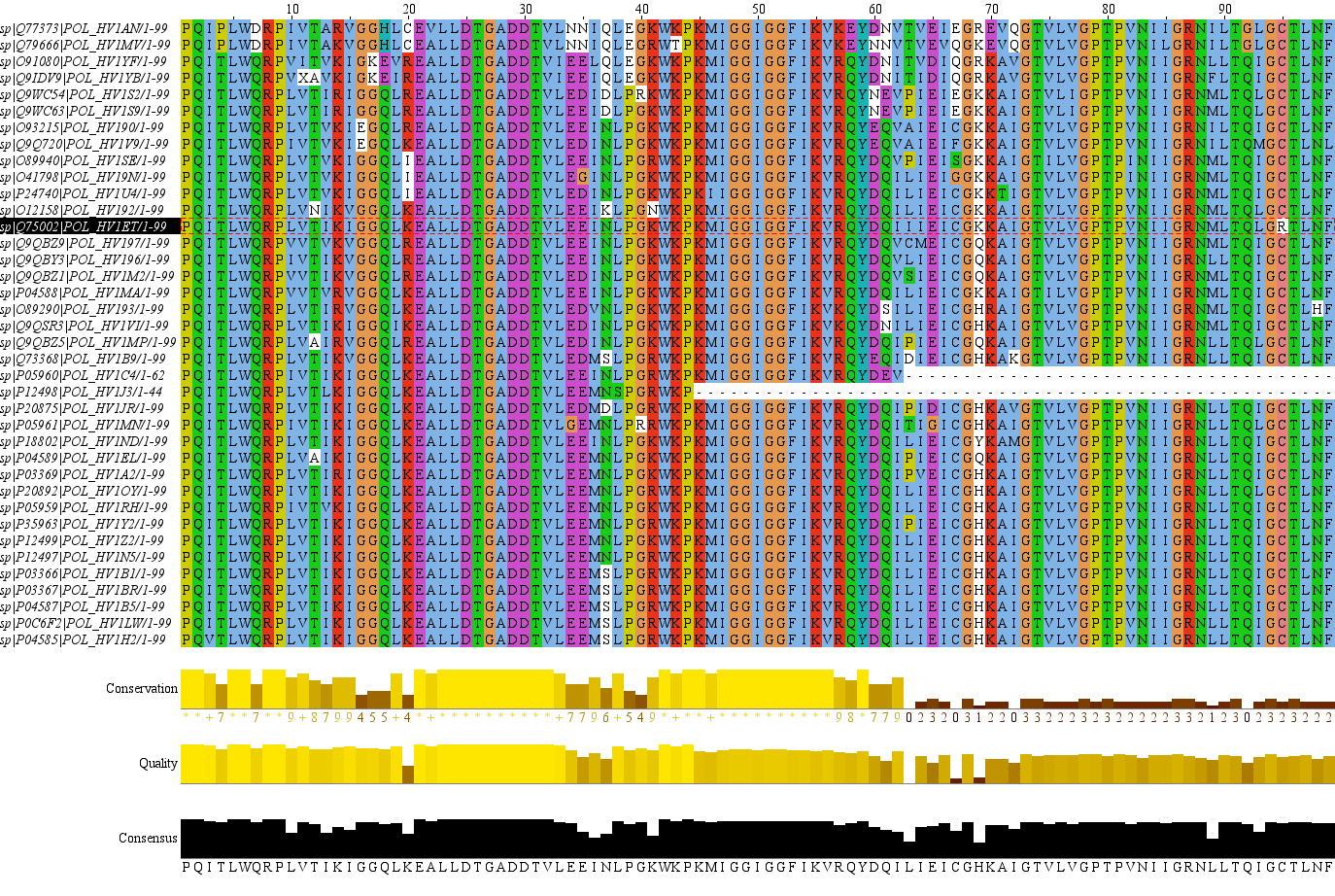


**Supplementary Figure 2.** The sequence alignment illustration of protease.


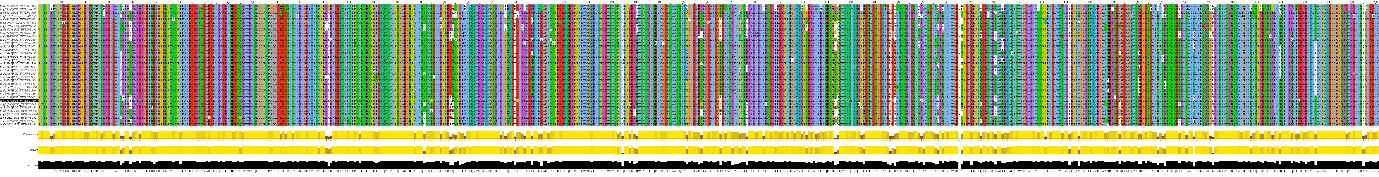


**Supplementary Figure 3.** The sequence alignment illustration of reverse transcriptase.


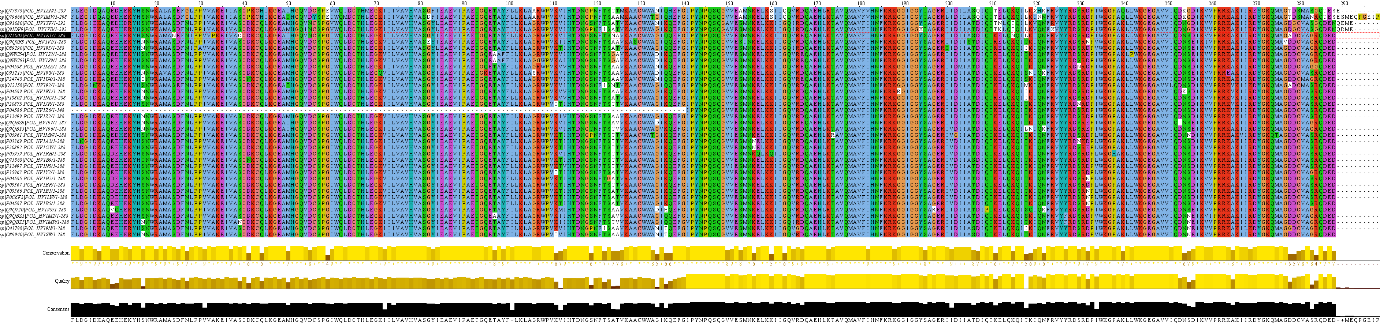


**Supplementary Figure 4.** The sequence alignment illustration of integrase.


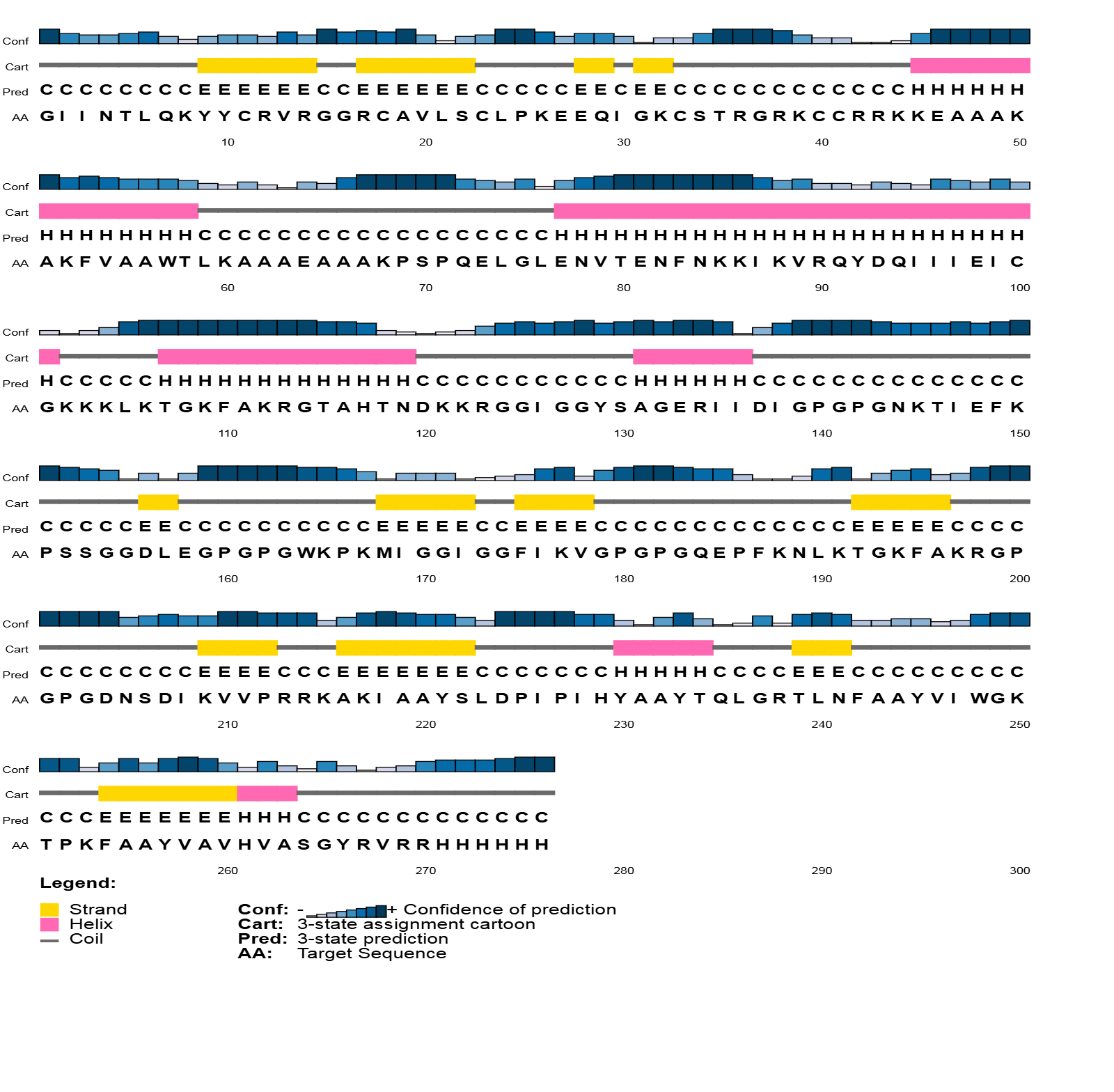


**Supplementary Figure 5.** Illustration of secondary composition based on their attributes of the non-mutated vaccine.


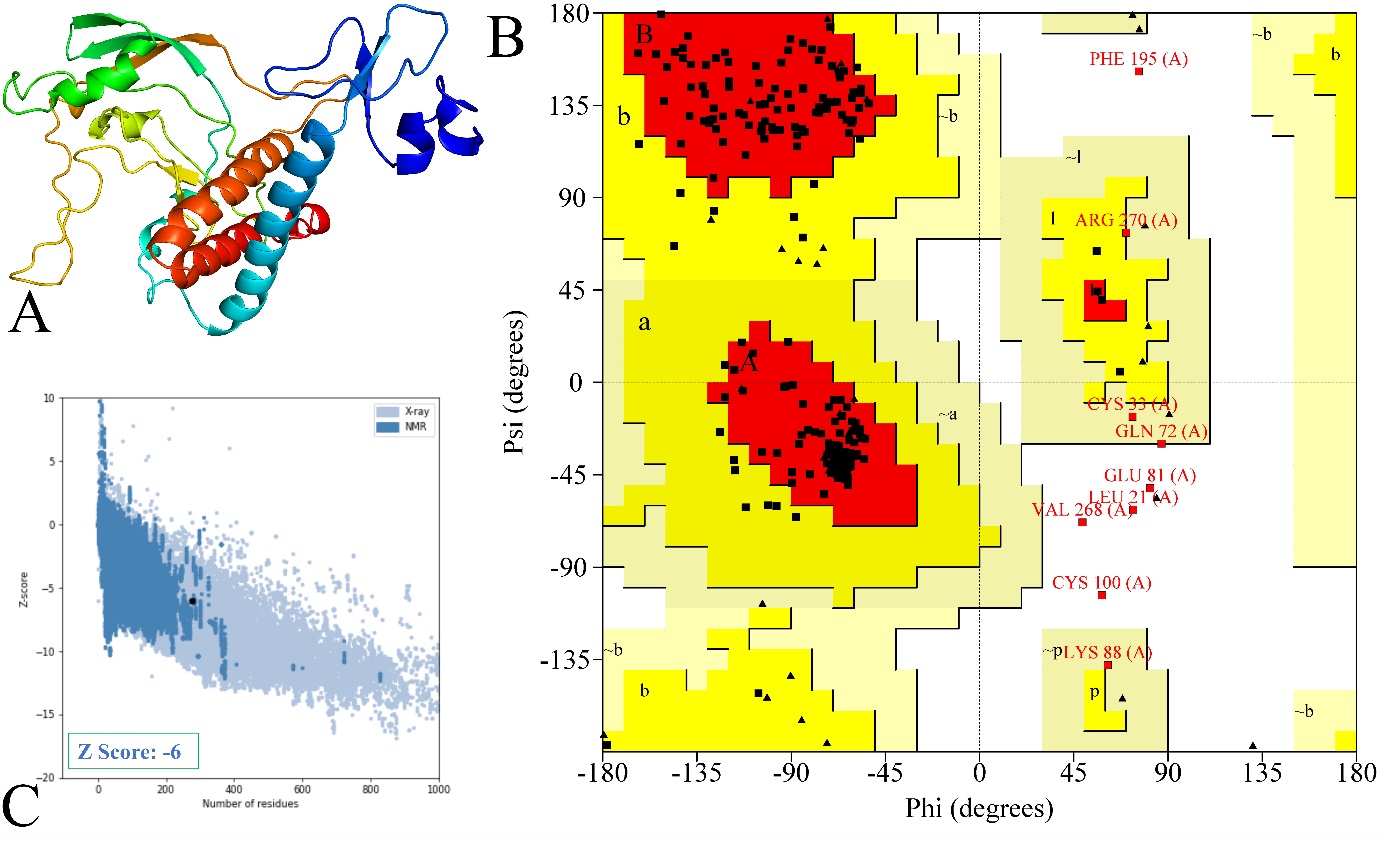


**Supplementary Figure 6.** Illustration and modelled non-mutated vaccine and their quality assessments. (A) Designed vaccine model, (B) Residue representation in various regions, (C) Quality evaluation via Z-score value.


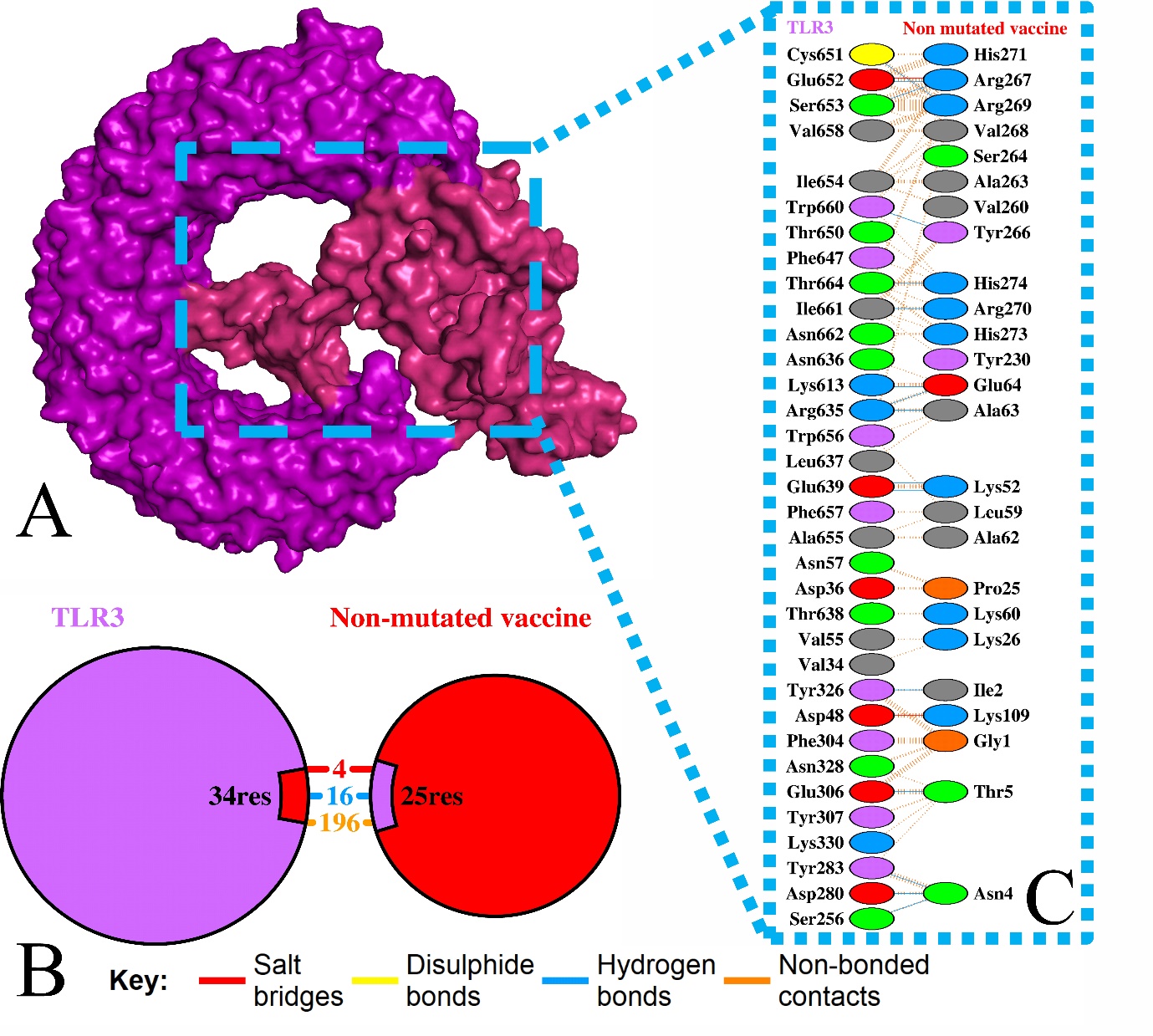


**Supplementary Figure 7.** Illustration of TLR3 with non-mutated vaccine. (A) surface interaction, (B) interface residue connection, and (C) Residual interaction.


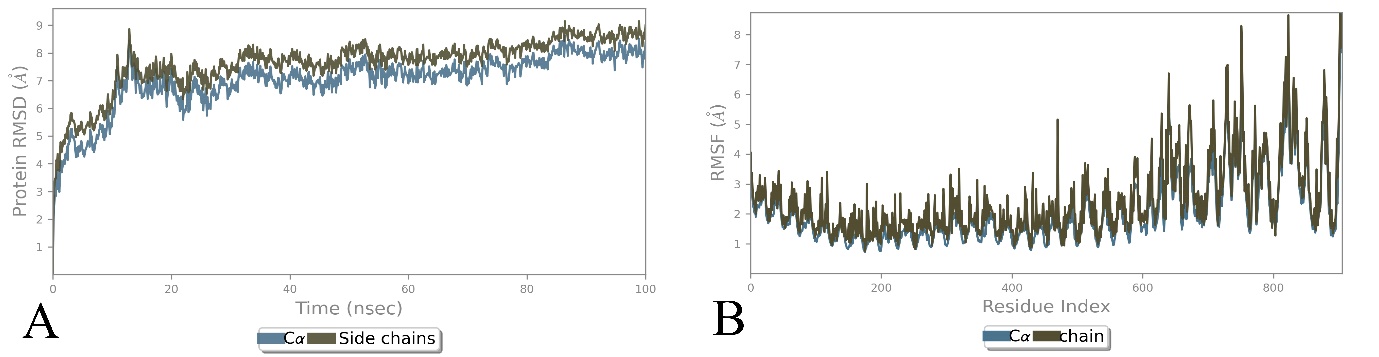


**Supplementary Figure 8.** Illustration of simulation-based investigation of the docked complex (Non-mutated vaccine with TLR3). (A) The RMSD-based trajectories analysis of complex, and (B) RMSF-based trajectories analysis of complex.


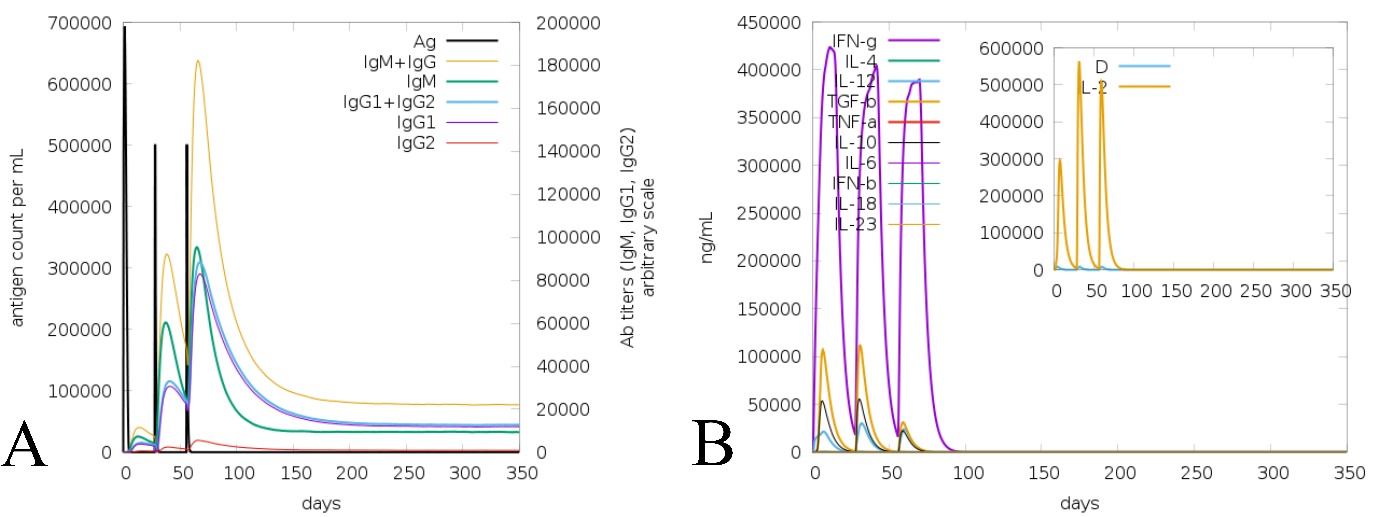


**Supplementary Figure 9.** Illustration of immune activity response of the non-mutated vaccine considering injection steps. (A) Vaccine-assisted antigen and antibody level  (B) Generated cytokine and interleukins level


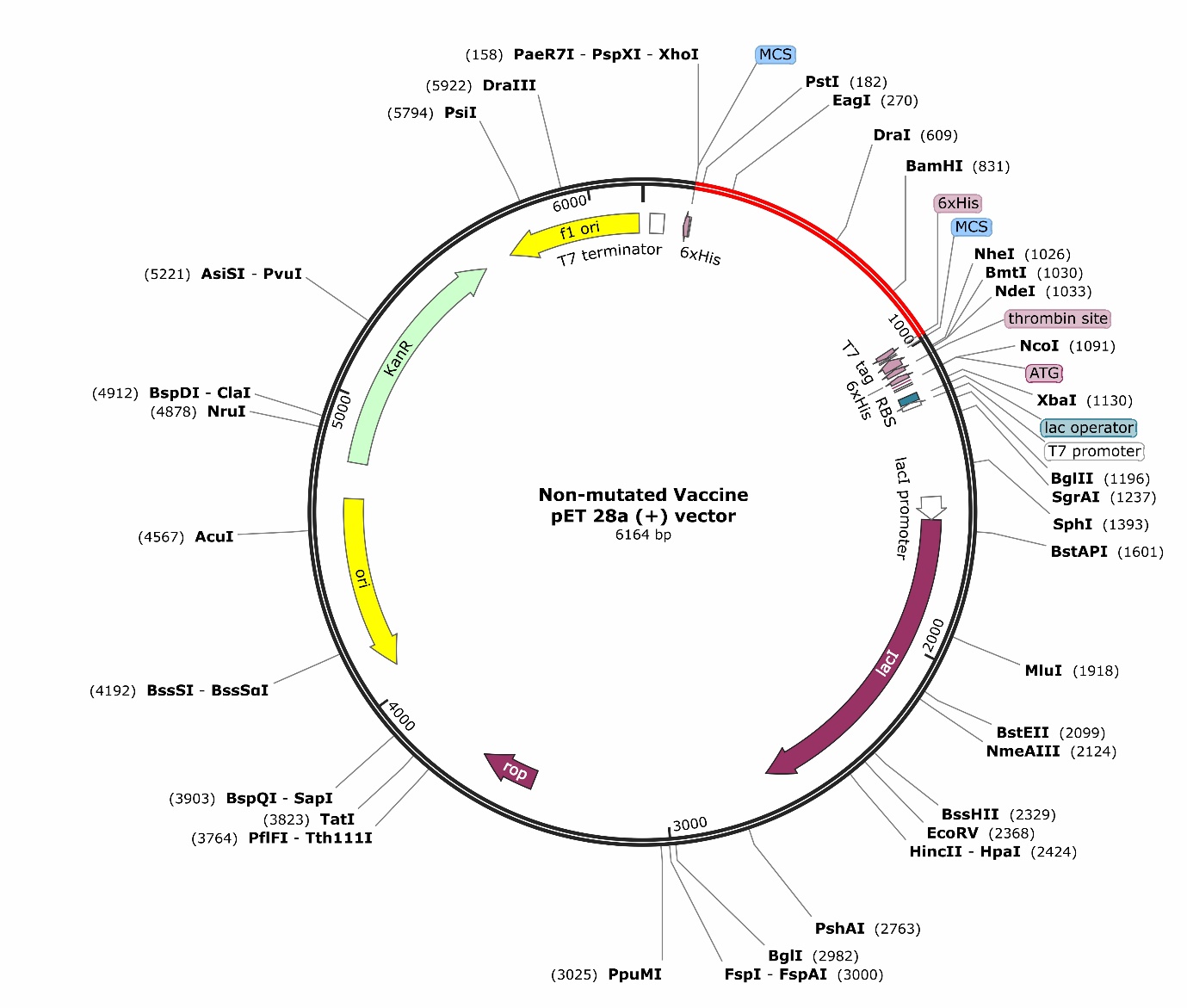


**Supplementary Figure 10.** Illustration of incorporated non-mutated vaccine in the pET28a(+).
